# Current status of global conservation and characterisation of wild and cultivated Brassicaceae genetic resources

**DOI:** 10.1093/gigascience/giae050

**Published:** 2024-08-07

**Authors:** Elena Castillo-Lorenzo, Elinor Breman, Pablo Gómez Barreiro, Juan Viruel

**Affiliations:** Royal Botanic Gardens, Kew, Wakehurst, Partnerships department, Ardingly, Haywards Heath, West Sussex, RH17 6TN, UK; Royal Botanic Gardens, Kew, Wakehurst, Partnerships department, Ardingly, Haywards Heath, West Sussex, RH17 6TN, UK; Royal Botanic Gardens, Kew, Wakehurst, Science Operations, Ardingly, Haywards Heath, West Sussex RH17 6TN, UK; Royal Botanic Gardens, Kew, Richmond, Research department, Surrey, TW9 3AE, UK

**Keywords:** crop wild relatives, cross-compatible, phylogenetic distances, plant conservation, cultivated Brassicaceae, breeding

## Abstract

**Background:**

The economic importance of the globally distributed Brassicaceae family resides in the large diversity of crops within the family and the substantial variety of agronomic and functional traits they possess. We reviewed the current classifications of crop wild relatives (CWRs) in the Brassicaceae family with the aim of identifying new potential cross-compatible species from a total of 1,242 species using phylogenetic approaches.

**Results:**

In general, cross-compatibility data between wild species and crops, as well as phenotype and genotype characterisation data, were available for major crops but very limited for minor crops, restricting the identification of new potential CWRs. Around 70% of wild Brassicaceae did not have genetic sequence data available in public repositories, and only 40% had chromosome counts published. Using phylogenetic distances, we propose 103 new potential CWRs for this family, which we recommend as priorities for cross-compatibility tests with crops and for phenotypic characterisation, including 71 newly identified CWRs for 10 minor crops. From the total species used in this study, more than half had no records of being in *ex situ* conservation, and 80% were not assessed for their conservation status or were data deficient (IUCN Red List Assessments).

**Conclusions:**

Great efforts are needed on *ex situ* conservation to have accessible material for characterising and evaluating the species for future breeding programmes. We identified the Mediterranean region as one key conservation area for wild Brassicaceae species, with great numbers of endemic and threatened species. Conservation assessments are urgently needed to evaluate most of these wild Brassicaceae.

## Introduction

Improving crops to face biotic and abiotic stresses, as well as enhance their nutritional value, is essential for ensuring global food security [1]. Ongoing biodiversity loss or decline can have a detrimental effect on future food security, and natural diversity provides resources to overcome challenges to food production such as environmental changes, pests, and diseases or limited land availability [2]. Crop wild relatives (CWRs) hold a wealth of genetic diversity that can be used to improve and help adapt traditional crops to succeed under environmental changes, making them of paramount importance for research and conservation [3, 4]. For example, CWRs have recently been used to transfer key traits to common crops in breeding programmes, such as tolerance and resilience to diseases and abiotic stresses such as salt or drought conditions [5] (and references therein). Previous studies have focused on major crops such as pulses, cereals, and forages, as well as their respective CWRs [6], and relatively little work has been done on oil crops, vegetables or fruits, and minor crops, such as those found in the Brassicaceae family. To date, this family has more than 300 accepted genera and around 4,000 species [7, 8], and it possesses a wide array of genetic diversity. It owes its economic importance to the widespread use of edible root crops, vegetables, and oilseeds [9].

Due to the importance of the family, several phylogenetic studies have aimed to unravel the systematics within Brassicaceae, and several taxonomic circumscriptions have been proposed to divide the family in tribes and to recognise relationships between species [10–14]. In a revision of the family, Al-Shehbaz et al. [15] divided Brassicaceae into 25 tribes but highlighted that further revisions might be required due to the large number of species (400) and genera (100) not yet sequenced. The tribe Brassiceae, formed by 8 clades, is the most studied because it includes the *Brassica* complex or U’s triangle [16, 17], which is formed by 6 globally important species of the genus that share 3 core genomes, termed A, B, and C, that have evolved independently [18]. These studies are useful to understand the phylogenetic relationships between wild and cultivated species, but also to estimate cross-compatibility between them to enable future breeding [19, 20]. However, further sampling efforts and sequence data are still required.

### Classification of CWRs

Transfer of genes between CWRs and crops can be challenging because of reproductive barriers between each pair of species [21]. Thus, knowing the level of cross-compatibility between CWRs and their respective crop(s) is key to transferring desirable traits using traditional breeding approaches. There are 3 different methods to classify CWRs, each aiming to identify the cross-compatibility of a CWR to its respective crop. The most important and accurate classification was proposed by Harlan and de Wet [22], which relies on actual crossing data between a crop and a wild species. They classified CWRs in gene pools (GPs): GP1 corresponds with cross-compatible individuals of the same species as the crop, GP2 represents a successful cross-pollination between a CWR and a crop, and GP3 is generally not compatible or results in sterile hybrids. However, producing this type of data is challenging, requiring living samples and investment. Thus, other classifications have been proposed when these resources are lacking. The taxon group (TG) classification [23] aims to estimate evolutionary relatedness based on taxonomic relationships and hierarchy. Four taxon groups were proposed to classify CWRs in the same genus as the crop to be cross-compatible; however, taxonomic circumscriptions do not necessarily reflect phylogenetic relationships. More recently, Viruel et al. [24] proposed the use of the phylogenetic distances to estimate cross-compatibility, where shorter phylogenetic distances between species equate to a greater possibility for them to be cross-compatible. This is a useful tool, especially when there is no information available for the GP classification.

### Characterisation of Brassicaceae species

Brassicaceae possesses a wide variety of crops and cultivated species; some of the most important ones are within the *Brassica* genus. Cabbage, broccoli (*Brassica oleracea* L.), turnip (*Brassica rapa* L.), rapeseed (*Brassica napus* L.), and mustard (*Brassica juncea* L. Czern.) are the main crops and most economically important within the family. There are breeding needs in agriculture that target different traits of these crops, such as resistance to biotic diseases, adaptation or tolerance to abiotic stresses, and improving or enhancing agronomic and functional traits [25].

Efforts to characterise CWRs physiologically and phenotypically have increased in recent years describing traits of interest. However, these often focus on plant growth, leaf characterisation and composition, dispersal syndrome and a few traits related to seeds such as germination, storage behaviour, and mass (TRY database [26]). As a result, there is less information on their tolerance to biotic and abiotic stresses compared to other key traits. Cultivated Brassicaceae are affected by biotic and abiotic hazards that cause loss of yield and poor performance in the field (especially pests [27] and diseases [28, 29]). CWRs of the Brassicaceae family are known to host desirable agronomic traits (compiled in [25, 30–32]) and abiotic stress tolerance such as to drought and salinity [33–35]. Different successful crosses have been performed to transfer some of these traits between *Brassica* crops and wild species [36–40]. The potential advantages of these crosses are not limited to agronomic traits and include health-related applications; a CWR of broccoli, *Brassica villosa* Biv., for example, has been used to increase anticancer compounds in a new variety [41].

Understanding the cross-compatibility between a CWR and a crop is essential for identifying the breeding techniques required to incorporate traits from wild species into the cultivar. However, publications of successful sexual crosses between crops and CWRs of Brassicaceae family are very limited (reviews mainly on *Brassica* genus [39, 42, 43]), which could be due to the complexity of the process [31] and the lack of knowledge and characterisation of Brassicaceae CWRs. Therefore, it is critical to characterise and understand the cross-compatibility between CWRs and crops of Brassicaceae, as well as to preserve potential CWRs to facilitate their accessibility and conservation for future sustainable use.

### Conservation and accessibility

Many CWRs are threatened with extinction by a range of factors such as land use and environmental changes, overexploitation, or invasive species [44]. Kell et al. [45] urged for conserving at least 78% of the known CWRs in Europe and suggested increasing the use of data on population distribution, trends, and size, as well as threat status, to design effective conservation plans. This would require information on the global distribution of wild genetic resources and their current *ex situ* representation in genebanks. Previous studies have identified gaps for specific areas (e.g., Indonesia [46], United States [47], Middle East [48], Europe [49]) or for specific crops (e.g., *Hordeum* [50], *Capsicum* [51], *Solanum* [52]) and landraces [53]. In the past decade, there has been an increase in *ex situ* conservation of CWRs [4, 6, 20, 54]. However, CWRs of major and minor crops have yet to be conserved, especially from the Brassicaceae family, which contains 70 priority CWRs for 17 crops [20]. Although there are scientific publications on *Brassica* crops, varieties, landraces, and wild relatives [30, 43], there has been very little focus on the characterisation and conservation of minor crops or on the evolutionary relationships between crops and wild Brassicaceae.

In this study, we aim to review the current classification of CWRs in Brassicaceae, identify new CWRs potentially cross-compatible with cultivated Brassicaceae estimated by phylogenetic distance, and describe the current geographic distribution and *ex situ* conservation status of CWRs in Brassicaceae.

## Results

### Gaps in genetic sequence data and phylogeny

**Figure 1:**
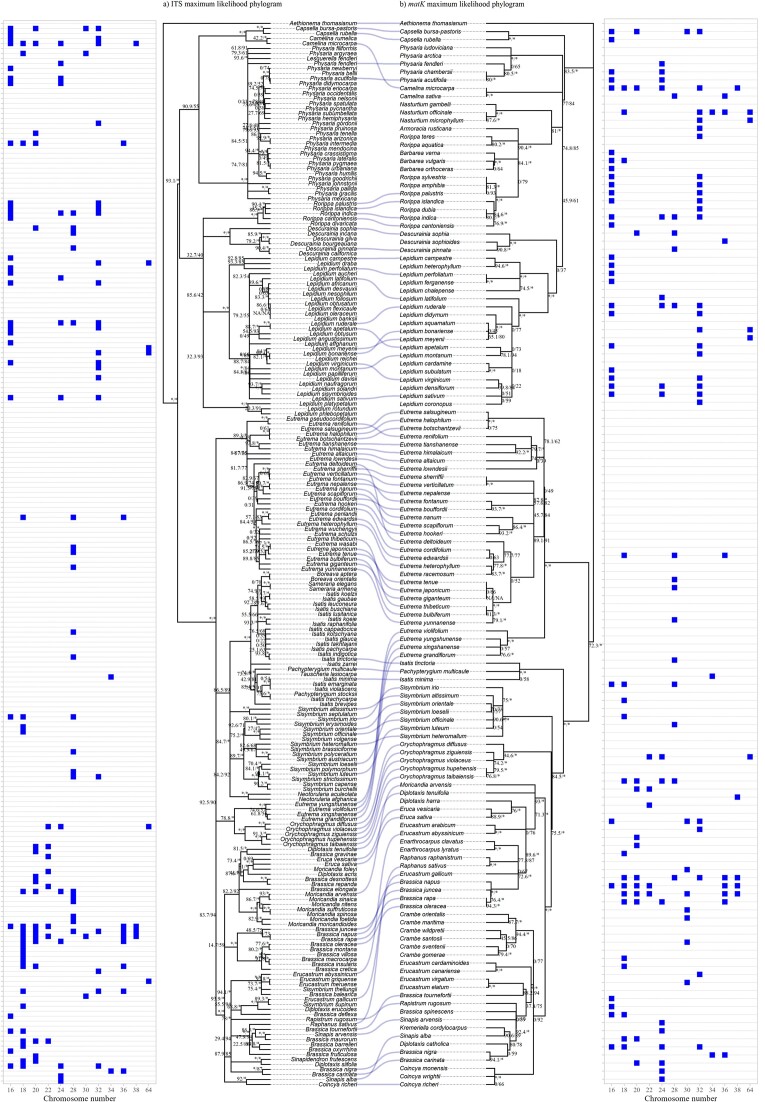
A comparison of 2 phylogenetic trees, nuclear marker ITS (A) and plastid marker *mat*K (B), reconstructed using maximum likelihood approaches for the data available in the GenBank for Brassicaceae. When the bootstrap is greater than or equal to 95, it is represented with an asterisk. Chromosome numbers reported in the literature are shown for each species. See Supplementary Data D3 for more details.

We have obtained DNA sequence data for 30% of the species (348 spp. out of a total 1,242 spp.) for 4 DNA regions: *rbc*L (175 spp.), *mat*K (162 spp.), ITS (241 spp.), and *trn*L-F (277 spp). Phylogenetic trees were built independently for each 4 genetic markers (Supplementary Fig. S1), and the phylogenetic trees with the highest resolution and bootstrap support were obtained using *mat*K data (with 131 taxa from the total of 162) and ITS data (214 taxa from the total of 241). In both phylogenetic trees, the tribes and groups were clearly divided in concordance with those defined in previous studies. A comparison of both phylogenetic trees was built to identify similarities and disparities between them (containing 85 common species, Fig. 1). Although the phylogenetic tree for ITS marker had more DNA sequence data, the genera *Armoracia, Barbarea, Crambe*, and *Nasturtium* were only present in the plastid marker *mat*K (Fig. 1). On the other hand, there were more DNA sequences available from the genera *Physaria* and *Isatis* for the ITS marker.

### Identification of potential cross-compatible CWRs

In this study, we distinguished successful conventional crosses of CWRs and crops that will produce hybrids from crosses that will require biotechnology techniques (e.g., *in vitro* culture, embryo, or ovary rescue). A summary of previous GP and TG classifications for CWRs in Brassicaceae, including 265 taxa (20% of the total 1,242), is listed in Supplementary Table S1. However, there were some wild species assigned as GP2 where no evidence of successful conventional cross-pollination has been published—for example, for *B. elongata* Ehrh. as a GP2 of turnip (*B. rapa*, Table 1 and Supplementary Table S1) with no information of crosses between them. Thus, to apply phylogenetic distances (PDs) as a proxy for cross-compatibility, we only use as a reference CWRs with evidence of successful sexual crosses with the crop. The PD thresholds were specific for each crop and ranged from 0 (the closest species in the phylogenetic tree) to 0.19 (the furthest cross-compatible CWR, Table 1) to predict potential cross-compatible CWRs. For example, white mustard, *Sinapis alba* L., and the wild species *Kremeriella cordylocarpus* (Coss. & Durieu) Maire are suggested to be cross-compatible (Table 1, PD = 0.0096), because pairwise phylogenetic distances were lower than other known CWRs with reported successful crosses with the crop (Table 1, PD <0.0125). Using this method based on phylogenetic distances, we propose 103 new potential CWRs (Table 1; see Supplementary Data D1 for more details ) to be cross-compatible with 18 cultivated species.

**Table 1:** List of cultivated Brassicaceae and potential CWR (crop wild relatives) species based on PDs between them for the 2 genes used in the phylogenetic tree, *mat*K and ITS from Fig. 1. (Y) represents conventional crosses reported for classified CWRs (based on gene pool classification listed in Supplementary Table S1), (Y*) represents conventional crosses with very low success, and (N) are unsuccessful conventional crosses (or crosses that needed biotechnology). NI means no information was found for their crosses [37, 42, 94 and references within them]. We represented with NA the species that were not present in 1 or both trees. Authorships and IDs for the scientific name of the species are listed in the Supplementary Data file. Bold taxa represent the new CWRs identified using the PDs (to see the complete and detailed list, please view Supplementary Data [122]).

Crop	CWRs *mat*K	PD	CWRs ITS	PD
*Barbarea verna* (TG)	***B. orthoceras, B. vulgaris***	0.00428	NA	NA
*Barbarea vulgaris* (TG)	***B. orthoceras***	0.00369	NA	NA
	*B. verna*	0.00428		
*Brassica carinata*	*Brassica nigra (Y)*	0.00187	*Brassica nigra (Y)*	0.02790
	***Diplotaxis catholica***	0.01131	***B. deflexa***, *B. maurorum, Coincya tournefortii*, ***B. balearica***, *B. fruticulosa*, ***B. oxyrrhina****, B. barrelieri*, ***Diplotaxis spp., Erucastrum spp***., *Sinapis spp., Raphanus spp.*, ***Rapistrum rugosum***	0.14164
	***Kremeriella cordylocarpus***, *Sinapis alba*	0.01251	*B. napus (Y), B. juncea (Y), Brassica spp.*, ***Moricandia spp***.	0.16518
	***B. spinescens, Rapistrum rugosum***, *Sinapis arvensis*	0.0158–0.01592	*Orychophragmus violaceus (Y)*	0.19612
	*Coincya tournefortii*, ***Erucastrum spp., Coincya spp., Crambe spp***.	0.02118–0.02505		
	*Orychophragmus violaceus (Y)*, ***Enarthrocarpus lyratus****, Eruca spp., Raphanus spp., B. napus (Y), B. juncea (Y)*	0.03134		
*Brassica juncea*	B. rapa (Y*), B. oleracea (Y), B. napus (Y)	0.00228–0.00246	B. rapa (Y*), B. napus (Y)	0.02807
	***Enarthrocarpus spp*** *., Raphanus spp*.	0.01692	*B. insularis, B. macrocarpa, B. villosa, B. cretica, B. oleracea, B. montana*	0.11898
	*Erucastrum spp., Diplotaxis spp., Coincya spp., Eruca spp., B. carinata (Y), B. nigra (Y), Coincya tournefortii*, ***Crambe spp****., Sinapis spp., Orychophragmus violaceus (Y)*, ***Sisymbrium spp., Kremeriella cordylocarpu******s***, ***Rapistrum rugosum***	0.0256–0.03454	*B. carinata (Y), B. nigra (Y)*, ***B. deflexa****, Coincya tournefortii, B. barrelieri, Eruca spp.*, ***Erucastrum spp., Moricandia spp****., Raphanus spp., Sinapis spp*.	0.16518
			*Orychophragmus violaceus (Y)*	0.19612
*Brassica napus*	*B. juncea (Y), B. rapa (Y), B. oleracea (Y)*	0.00246	*B. rapa (Y)*	0.000003
	***Enarthrocarpus spp., Erucastrum spp*** *., Raphanus spp*.	0.01692	*B. juncea (Y)*	0.02807
	*Eruca spp.*, ***Diplotaxis spp****., B. carinata (Y), Coincya tournefortii, B. nigra*, ***Crambe spp***.	0.02103–0.03134	*B. insularis, B. macrocarpa, B. villosa, B. cretica, B. oleracea (Y), B. montana*	0.11898
			***B. deflexa, Erucastrum spp*** *., B. carinata (Y)*, ***Diplotaxis spp***., ***Sisymbrium spp****., Sinapis spp.*, ***Moricandia spp***.	0.16518
*Brassica nigra*	*B. carinata (N)*	0.00187	*B. carinata (N)*	0.02790
	***Diplotaxis spp., Kremeriella cordylocarpus*** *, Sinapis alba*	0.01131–0.0125	***B. deflexa*** , B. maurorum (Y*), Sinapis arvensis (Y*), Coincya tournefortii, ***B. oxyrrhina****, B. barrelieri*, ***B. balearica****, B. fruticulosa*, ***Diplotaxis spp., Erucastrum spp., Rapistrum rugosum****, Raphanus spp.*, ***Rapistrum rugosum***	0.14146
	***B. spinescens*** , Sinapis arvensis (Y*), ***Rapistrum rugosum***	0.01578	*B. juncea (Y)*, ***Moricandia spp***., *Brassica spp*.	0.16518
	***Coincya spp., Crambe spp*** *., B. napus, B. oleracea (N), B. juncea (Y), B. rapa*, ***Moricandia arvensis***	0.02118–0.03134		
*Brassica oleracea*	*B. rapa (Y)*	0.000002	*B. montana (Y)*	0.00405
	*B. juncea (Y), B. napus (Y)*	0.00228–0.00246	*B. insularis (Y), B. macrocarpa (Y), B. villosa (Y), B. cretica (Y)*	0.02898–0.03112
	***Enarthrocarpus spp*** *., Raphanus spp.*, ***Erucastrum spp****., Eruca spp*.	0.01692–0.0260	*B. juncea (Y), B. rapa (Y), B. napus (Y)*	0.11898
	*Coincya tournefortii (Y), B. nigra*, ***Coincya spp., Erucastrum spp., Crambe spp***.	0.03134	*Coincya tournefortii (Y), B. nigra*, ***Brassica spp., Erucastrum spp****., Raphanus sativus*	0.16518
*Brassica rapa*	*B. oleracea (Y)*	0.000002	*B. napus (Y)*	0.000003
	B. juncea (Y*), B. napus (Y)	0.00228–0.00246	B. juncea (Y*)	0.02807
	***Enarthrocarpus spp*** *., Erucastrum gallicum (Y), Raphanus spp*.	0.01692	*B. oleracea (Y), B. macrocarpa, B. villosa, B. cretica, B. montana, B. insularis*	0.11898
	*Erucastrum spp., Diplotaxis spp., Coincya spp., C. tournefortii, B. carinata (Y), B. nigra*, ***Crambe spp., Enarthrocarpus spp***., *Eruca spp., Sinapis spp*.	0.02103–0.03135	Erucastrum gallicum (Y), B. barrelieri (Y), B. carinata (Y), B. elongata (NI), B. fruticulosa (Y*), ***Brassica spp***., ***Diplotaxis spp****., Sinapis spp., Eruca spp.*, ***Moricandia spp., Erucastrum spp***.	0.16518
*Diplotaxis tenuifolia*	*B. oleracea, B. rapa (Y), B. juncea (Y)*, ***Enarthrocarpus spp***., *Erucastrum spp., Eruca spp.*, ***Moricandia arvensis***	0.02599	***B. gravinae***	0.08368
	*B. nigra (Y)*, ***Coincya spp., Crambe spp***.	0.03134	***B. repanda, B. desnottesii*** *, Eruca spp.*, ***Diplotaxis acris, Moricandia spp***.	0.10650–0.11255
			*B. juncea (Y), B. rapa (Y), B. nigra (Y)*, ***Erucastrum spp., Diplotaxis spp****., Raphanus sativus, Sinapis spp.*, ***Brassica spp***.	0.16518
*Eruca vesicaria*	*E. sativa*	0.00254	*E. sativa*, ***E. foleyi***	0.06195
	***Diplotaxis harra***	0.01327	***Diplotaxis acris, Brassica repanda, B. desnottesii***	0.08016–0.09699
	*Brassica napus, B. juncea, B. rapa, B. oleracea*, ***Enarthrocarpus spp****., Raphanus spp. Diplotaxis tenuifolia (Y)*, ***Moricandia arvensis***	0.02103–0.02599	***Brassica gravinae*** *, Diplotaxis tenuifolia (Y)*, ***Moricandia spp., Brassicaelongata***	0.10650–0.11250
*Eutrema japonicum*	***E. giganteum, E. tenue*** *(NI)*	0.000002	*E. wasabi*	0.000002
	***E. thibeticum, E. bulbiferum, E. yunnanense*** *(NI)*	0.00349	***E. tenue*** *(NI)*	0.00656
			***E. bulbiferum***	0.01620
			***E. yunnanense*** *(NI)*, ***E. thibeticum, E. giganteum, E. schulzii, E. wuchengyii***	0.03965
*Isatis tinctoria* (TG)	***I. minima*** *(NI)*, ***I. multicaulis***	0.01772	***I. indigotica***	0.003760
			***I. pachycarpa, I. takhtajanii, I. glauca, I. kotschyana, I. cappadocica***	0.01646
*Lepidium meyenii*	***L. bonariense*** *(NI)*, ***L. squamatum, L. disymum***	0.00107–0.00509	***L. reichei, L. bonariense*** *(NI)*, ***L. virginicum***	0.01302
	***Lepidium spp***.	0.01359	***Lepidium spp***.	0.07325
*Lepidium sativum*	***L. virginicum, L. densiflorum, L. coronopus***	0.008112	***Lepidium spp***.	0.07560
	***Lepidium spp***.	0.01359–0.03284		
*Nasturtium officinale* (NI)	***N. microphyllum***	0.000002	NA	NA
	***N. gambelii***	0.00505		
*Raphanus raphanistrum subsp. sativus*	*Raphanus sativus (Y)*	0.00184	*Brassica spp., Sinapis arvensis*	0.07473
	Brassica napus (Y*), ***Enarthrocarpus spp***., ***Erucastrum spp***., *Brassica spp*.	0.01692	*Brassica spp.*, ***Diplotaxis spp., Erucastrum spp***., B. napus (Y*)	0.14164–0.16518
*Rorippa indica* (TG)	***R. dubia***	0.000002	***R. islandica, R. palustris***	0.01775
	***R. cantoniensis, R. islandica***	0.00391–0.00406	***R. cantoniensis***	0.02508
	***R. palustris*** *, R. amphibia*, ***R. sylvestris***	0.00507	***R. divaricata***	0.04479
*Sinapis alba*	*Kremeriella cordylocarpus*	0.00956	***Coincya richeri***	0.08614
	Brassica carinata, B. nigra (Y*), B. spinescens, ***Diplotaxis catholica****, Sinapis arvensis*	0.01251–0.01578	B. nigra (Y*), Diplotaxis spp., ***Erucastrum spp., Moricandia spp***.*, Eruca spp., Brassica spp*.	0.16518
	***Rapistrum rugosum, Erucastrum spp*** *., Coincya monensis (Y)*, ***Coincya spp., Crambe spp***.	0.02119–0.0251		
*Sisymbrium officinale* (NI)	***S. loeselii, S. orientale, S. luteum, S. altissimum***	0.00361	***S. volgense, S. orientale***	0.03702
			*Sisymbrium spp*.	0.09579

The potential cross-compatibility CWRs estimated using phylogenetic distances will need to be revised considering ploidy level variation, because Brassicaceae species have a large variation in chromosome numbers, from 2*n* = 150 (*Crambe gordjaginii* Sprygin & Popov) to 2*n* = 8 for some *Physaria* species (Fig. 1; for detailed information see Supplementary Data D3), and crosses between the same ploidy levels are recommended when possible. For example, using phylogenetic distances, we estimated that *Brassica gravinae* Ten. (2*n* = 20) is likely cross-compatible with turnip (*B. rapa*, 2*n* = 10 and 20 between others). We recommend using the same cytotype forms (2*n* = 20) to attempt crossing them. However, only 40% of the wild Brassicaceae species on this study had ploidy level or chromosome number information available (474 taxa), of which 122 are in ITS and 93 in *mat*K phylogenetic tree (55 in common, Fig. 1).

### Major crops

All the major crops listed in Table 1 were present in both phylogenetic trees and had successful conventional crosses reported that can confirm the cross-compatibility between some species. Since major crops had more information published, we identified potential CWRs based on the phylogenetic distances, including new genera and species not previously suggested that had shorter distances (Table 1) than the cross-compatible CWRs already identified (Supplementary Table S1).

### Minor crops

In general, there was very little information regarding successful conventional crosses between wild species and minor cultivated crop (perennial wall rocket, *Diplotaxis tenuifolia* (L.) DC., was the only exception). For some taxa, there were not enough sequences (*Barbarea* genus) or no DNA sequence data (e.g., cultivated *Crambe*); in others, the problem was the lack of information on the cross-compatibility as a reference on the phylogenetic tree (e.g., *Eutrema japonicum* (Miq.) Koidz., had many wild species with DNA sequence data but lacked referenced species as confirmed cross-compatible).

### Traits for breeding

**Figure 2:**
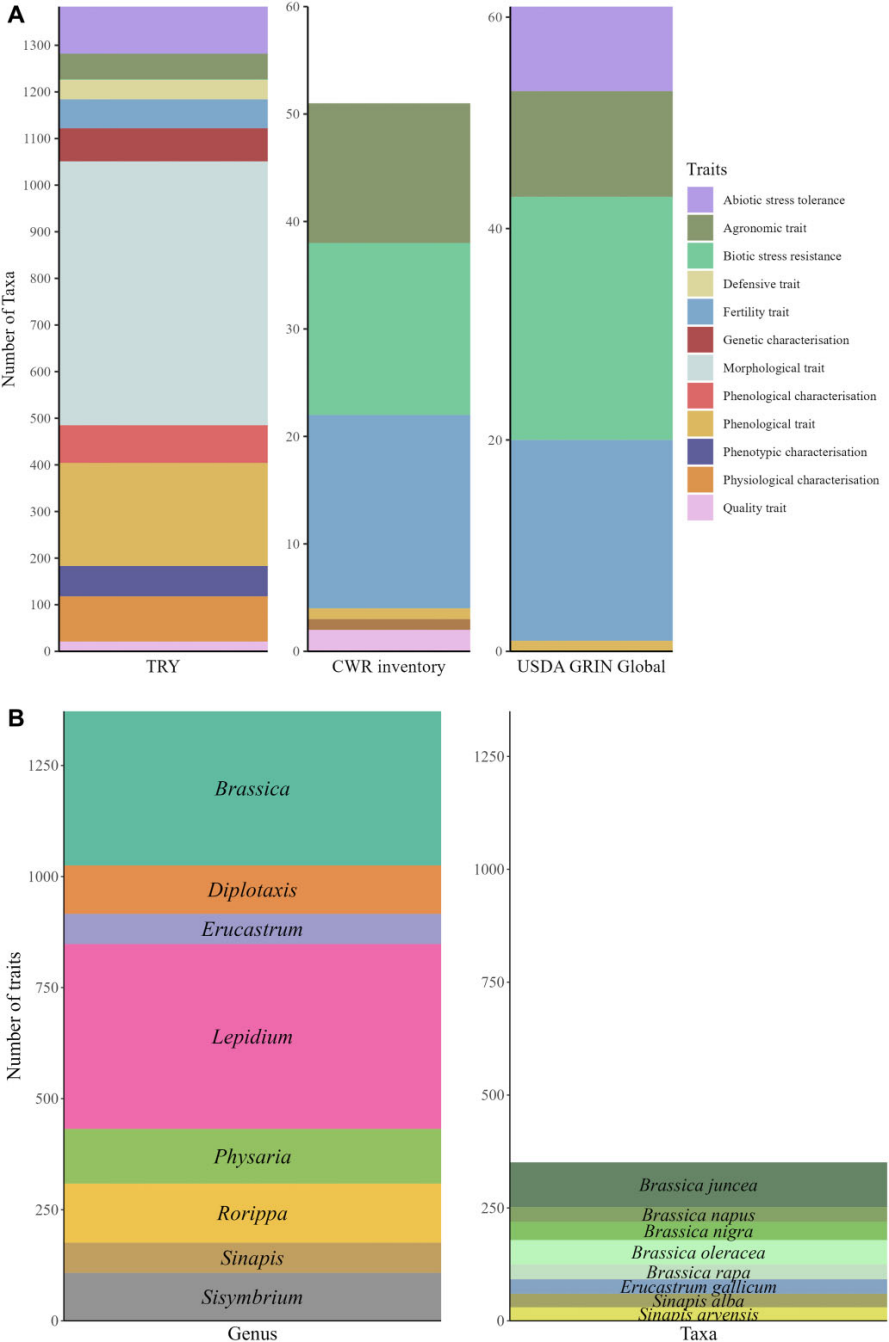
Top traits from global databases for all Brassicaceae available (A) and the top 8 genera and species with the most traits characterised (B). This information was obtained from a total of 599 taxa.

We identified gaps in the characterisation of the wild Brassicaceae species included in this study by compiling information in different databases to describe the most and least explored and characterised species. USDA-GRIN Global database on CWR [55] and Harlan and de Wet CWR inventory [56] compile traits of CWR and hold information for 14 cultivated Brassicaceae taxa (Supplementary Data D2) and 171 wild species related to them. Biotic traits are the most studied, followed by fertility traits; the combination of both represents 74% of the available data. The remaining 26% are abiotic and agronomic traits (Fig. 2A). Additionally, TRY database [26] shows more than 7,000 entries for wild Brassicaceae, and there is information on potential traits of 599 Brassicaceae species. The main traits captured focus on morphology and physiology (e.g., plant growth, flowering time, dispersal syndrome, Fig. 2A). The genus with the largest number of traits recorded and published is *Lepidium* (416), followed by *Brassica* (347, Fig. 2B). However, the top 5 species that were the most characterised, with more traits identified, are from the *Brassica* genus (5 of the 6 species that form the U’s triangle, Fig. 2B).

### Geographical conservation gaps of Brassicaceae

**Figure 3:**
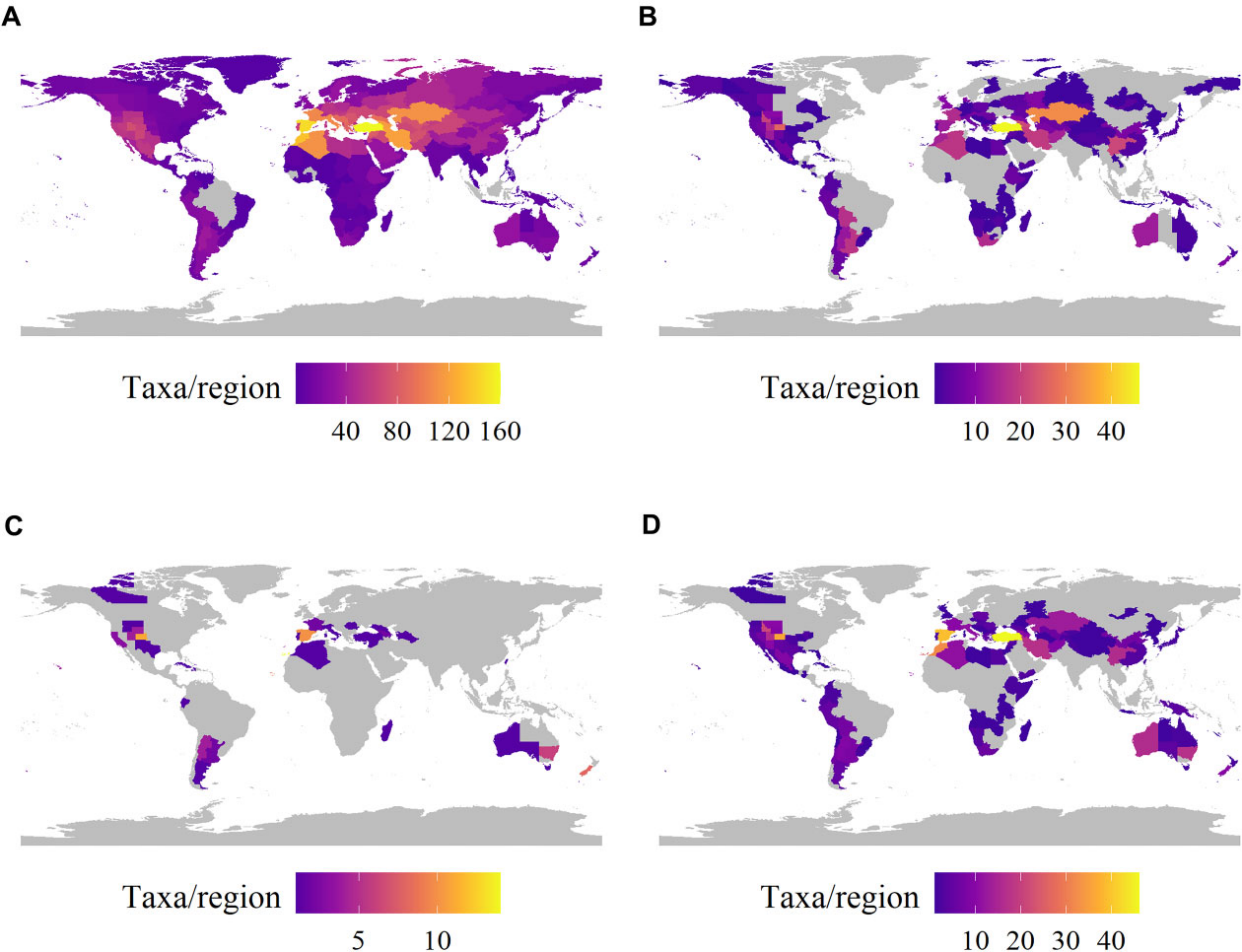
Global distribution of the 1,242 wild Brassicaceae species identified (A) native distribution, (B) distribution of species not conserved *ex situ*, (C) threatened species, and (D) endemic species. Gray areas represent regions where the populations are not present.

The distribution of all 1,242 studied populations confirms the global presence of wild Brassicaceae species, with some of them widely cultivated (i.e., *Brassica rapa, B. juncea, Raphanus raphanistrum* subsp*. sativus* (L.) Domin) or introduced. However, introduced species were removed from the analysis to focus on the native distribution of wild Brassicaceae. For the geographical distribution, we used TDWG (Biodiversity Information Standards) level 3, and the 3 regions with the greatest number of native taxa are Turkey (160 species), Spain (147), and Morocco (135, Fig. 3A). There are 787 species yet to be conserved *ex situ* (i.e., no records available on global databases) and more than 200 that are underrepresented (fewer than 5 populations conserved *ex situ*, Supplementary Data D1). The greatest number of taxa missing from *ex situ* collections occurs in Turkey (46), Kazakhstan (32), and Colorado regions (26, Fig. 3B).

Conservation status has been evaluated for only 440 species, of which ca. 30% are considered threatened (119 threatened, of which 110 are also endemic). The highest number of threatened taxa was found in the Canary Islands (14), peninsular Spain (10), Colorado (11), and Cape Verde regions (9, Fig. 3C; see Supplementary Data D1 for more details). Half of the taxa in the database represent single-region endemics (667 species). The greatest number of endemic species is found in Turkey (49), peninsular Spain (39), and Colorado regions (36, Fig. 3D; see Supplementary Data D1 for more details).

**Figure 4:**
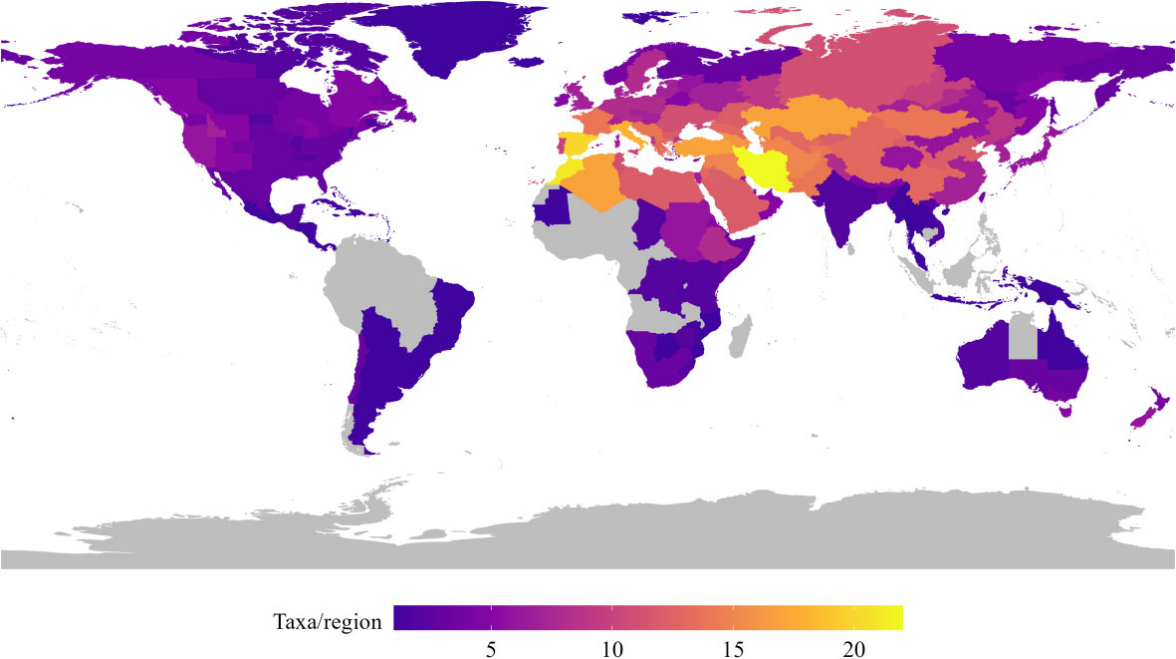
Global distribution of the new 103 estimated cross-compatible CWRs of Brassicaceae using phylogenetic distances. See Supplementary Data D1 for more details.

The geographical distribution of the new 103 proposed as cross-compatible CWRs has been defined (Fig. 4) and we observed that almost 70% of these species are not well represented in *ex situ* conservation or not represented at all (36%, Supplementary Data D1). Unfortunately, more than 70% of them have not being globally evaluated for their conservation status, and the level of threat of their populations is unknown (Supplementary Data D1).

## Discussion

### Identification of new potential CWRs in Brassicaceae from available DNA data

Various molecular markers have been used to reconstruct phylogenetic trees to distinguish between species and identify clades in Brassicaceae; the most frequently used are *rbc*L, *mat*K, ITS, and *trn*L-F. In general, *rbc*L is considered a slow evolving gene, *mat*K is intermediate, and ITS and *trn*L-F are evolving relatively faster [57]. The choice of markers used is based on the desirable outcome; for example, a combination of 2 markers, such as *rbc*L and *mat*K, has been suggested to build phylogenetic trees and identify species [58]. In the present study, we compared one nuclear marker (ITS) and 1 plastid marker (*mat*K, Fig. 1). A more comprehensive phylogeny was recently published using a larger Brassicaceae dataset (1 species per genus [11]). The cultivated Brassicaceae were well spread around the supertribes Camelinodae (I) and Brassicodae (II) of the phylogenetic tree [11, 13], with all major crops present in Brassicodae (II) (Supplementary Fig. S1). In our study, both phylogenetic trees (ITS and *mat*K) were congruent regarding the major clusters or groups formed, but there were a few discrepancies.

- The genus *Eutrema* was split in the phylogenetic tree ITS in 2 clades, with one containing most *Eutrema* species and a second with *E. violifolium* (H.Lév.) Al-Shehbaz & Warwick, *E. yungshunense* (W.T.Wang) Al-Shehbaz & Warwick, *E. xingshanense* (Z.E.Chao, Z.L.Ning, & X.W.Hu) G.Q.Hao, and Al-Shehbaz & J.Quan Liu and *E. grandiflorum* (Al-Shehbaz) Al-Shehbaz & Warwick grouped to a clade formed by the genus *Orychophragmus*. This is in agreement with other phylogenetic trees [59] where the genus *Eutrema* was split due to the geographical distribution of the species (all of them occur only in Central China) and were clustered with *Orychophragmus* taxa, which are mostly distributed in China. However, the phylogenetic tree reconstructed with *mat*K resolved *Eutrema* species in a monophyletic clade, although the aforementioned 4 species formed a subclade within the genus. This could reflect a different evolutionary history between nuclear and plastid markers (reflected by larger phylogenetic distances, Fig. 1), and those 4 species are likely to be noncompatible with wasabi crop (*Eutrema japonicum*), but further research is needed for this unexplored crop.
- The *Erucastrum* genus was scattered around the Brassiceae tribe in the *mat* K phylogenetic tree (Fig. 1) with *Erucastrum* species placed in both the Rapa/Oleracea and the Nigra clades, as reported in previous phylogenies [17, 32]. In general, only 2 species are well studied in this genus, *E. abyssinicum* (A.Rich.) O.E.Schulz and *E. gallicum* (Willd.) O.E.Schulz (the latter is widely distributed in the United States, Europe, and some areas of Asia [60]), and both were present in the two phylogenetic trees, *mat*K and ITS. The division in the *mat*K marker could be due to the distribution of the species, but more populations should be investigated to verify this. Additionally, some of them are edible [32] and may have been subjected to some type of selection, a potential further reason for their distribution across clades.

Chromosome numbers also play an important role to estimate cross-compatibility between species. The evolution of Brassicaceae species seems to be driven by whole-genome duplication events and polyploidy [61]. These polyploidy events are species and lineage specific and can affect the relationships of the species in the phylogeny. Polyploidy is present especially in the Brassiceae tribe [62]; for example, polysomaty or mixoploidy (having cells with different numbers of chromosomes in different tissues or cells) has been reported in *Brassica* and *Raphanus* genera [63]. There is a large disparity in chromosome numbers within the family [61, 62] (Supplementary Data D3), making ploidy data critical for the identification of cross-compatible species in addition to the phylogenetic distances. For example, variation in ploidy levels could be the reason why *Brassica oleracea*, a progenitor of *B. carinata* A. Braun, has few successful crosses and very low rates of hybrids produced [42].

Harlan and de Wet’s classification [22] of CWRs enables the identification of potentially compatible wild species. They defined secondary gene pool (GP2) species as those that will be able to transfer genes by conventional crosses, with some possible barriers or lower success rates. They also suggested that gene pools could be separated based on different ploidy levels, but to our knowledge, this approach has not been carried out, which could be challenging when species exhibit multiple ploidy levels. The literature and databases contain a mixture of “secondary” and “tertiary” (not compatible or resulting in sterile hybrids) CWRs, where no crosses are found in the literature, or with very limited success of crossing (Table 1), or even where biotechnology techniques (e.g., embryo rescue, ovary rescue, somatic hybrids) were required to obtain hybrids. Due to the complexity of the Brassicaceae family and the mixed classifications, it is more difficult for prebreeders to use some of these wild genetic resources, because in many cases, previous classifications were not validated by crosses. Thus, a detailed review of CWR lists corroborated with data from crosses is urgently needed to clarify our current knowledge of Brassicaceae CWRs. In this study, we compiled information of successful crosses, chromosome numbers, and phylogenetic distances (Fig. 1) to update the classification of CWRs in Brassicaceae and to identify new CWRs that are potentially cross-compatible with crops (Table 1). These newly identify CWRs, using phylogenetic distances, will require characterisation and evaluation for crossability with the crop.

### Major crops

As expected, there were more data and publications for well-known crops (*Brassica* U’s triangle, *Eruca, Sinapis, Raphanus*) and their CWRs than for minor crops, especially regarding breeding and agronomic traits and ploidy level. Despite the *Brassica* genus comprising most of the major crops, the phylogenetic relationships between species are still far from understood. Brassiceae is a polyploid tribe [64], and this is a challenge for taxonomists and geneticists, and more investigation is needed to resolve taxonomic issues and fully understand the cross-compatibility between species.

There are some incongruences on the cross-compatibility in the literature within *Brassica* U’s triangle. For example, despite being in different clades (Nigra and Oleracea, respectively [17], Fig. 1) and having longer phylogenetic distances, *B. nigra* and *B. oleracea* are classified as secondary CWRs to each other due to successful crosses between them, but only when the *B. nigra* was used as the female parent [42]. In general, most of the crosses that were successful within this tribe had the cultivated species as the female donor [42]. Another successful interspecies sexual hybridisation was published by Kumar et al. [37], between *B. rapa* and *B. fruticulosa* (*B. rapa* being the female parent), and this technique has been used by other researchers as a bridge to transfer resistance genes from *B. fruticulosa* to *B. juncea* [40]. *B. rapa* and *B. juncea* are classified as GP3, but successful crosses between them have been reported [65, 66]. On the other hand, *B. oleracea* is one of the species from which *B. napus* originated, but the crosses between them produced a very low number of hybrids [42]. However, *B. oleracea* has been successfully crossed (by conventional reproduction) with *B. cretica* Lam., *B. incana* Ten., *B. macrocarpa* Guss., *B. montana* Pourr., and *B. villosa* Biv. [67]. Nonetheless, further research is required to improve the success of gene transfer for this species.

There are also successful intergeneric crosses involving the *Brassica* genus such as attempts to cross with *Orychophragmus violaceous* [68, 69] even though the species is distant in the phylogenetic tree (Table 1). *Diplotaxis tenuifolia* and *Erucastrum gallicum* were also able to produce hybrids when crossing with *Brassica* species, although in some cases, these were only successful when *Brassica* was the female parent [70, 71]. Intergeneric crosses were also possible with *Raphanus* species and *Eruca vesicaria* (L.) Cav., but with low success, and in some cases, biotechnology techniques were required to overcome cross-compatibility barriers [72–74].

### Minor crops

Minor or less common cultivated species such as hedge mustard, cress cultivars, or Abyssinian kale are less widely cultivated, and thus, less information is available for them. *Crambe hispanica* subsp. *abyssinica* (Hochst. ex R.E.Fr.) Prina was not included in the phylogenetic tree due to lack of genetic sequence information. Similarly, characterisation and information about interesting traits within wild species of these minor crops are lacking in the literature. In some cases, there is an issue of self-incompatibility or sterile plants (*Armoracia* [75]), which makes the breeding process more complex. Due to the limited information about their ploidy levels, it is complicated to identify potential candidates to be cross-compatible with cultivated species. This is the case in the genus *Diplotaxis*, which possesses dysploidy (an organism that has an increased or decreased number of chromosomes, by 1 or more, than the original [76]).

However, these CWRs could hide a wide genetic diversity and future evaluation of their adaptation and traits would be useful. For example, the *Barbarea* genus is considered a great source of plant defence compounds within the family [77], and some species show resistance to several biotic stresses (mildew, nematodes, and thrips [78]). In other genera, medicinal compounds have been reported (*Isatis* [79], *Nasturtium* [80, 81], and *Sisymbrium* [82]), and the effect of different environmental conditions has been evaluated (*Isatis* [83], *Nasturtium* [84], and *Rorippa* [85]).

Probably due to the novelty of some of these crops, very little has been done to improve their characteristics, but also few traits have been characterised to understand the requirements (if any at this stage) to cultivate these species, and therefore, further investigation is needed, especially to understand and improve their performance and adaptation. Using available data for Brassicaceae, we have identified around 103 new potentially cross-compatible CWRs (Table 1; see Supplementary Data D1 for more details) for 18 crops, although, in general, more investigation is needed. More species will need to be sequenced and generate more data (e.g., physiological and phenotypic characterisation as well as acquiring knowledge of the ploidy level). This is key to understand the needs of the cultivated species and to identify CWRs with interesting traits. Confirming the cross-compatibility of the new potential CWRs with the same ploidy level is critical, in addition to generating more DNA sequencing data to complete the genetic characterisation of the family.

### Cultivated Brassicaceae limitations

A detailed characterisation of plant species is fundamental to understand the limitations of cultivated species. Combining phenotypic and genotypic data will positively impact on improving and transferring traits to major and minor crops, as reviewed by Katche et al. [39]. As for the compatibility data, phenotypic and genotypic characterisation is generally available for major crops and nonexistent or rare for less well-known cultivated species (*Crambe, Nasturtium*, or *Diplotaxis*). The exception observed in Fig. 2B is for *Lepidium*, which was one of the top 3 genera with more species characterised for at least 1 trait, but this could be due to the large number of accepted species included in the genus (up to 262 spp.).

The most studied traits were those related to the morphology and phenology of the plant in addition to agronomic traits and biotic stress resistance [36, 40, 73]. Despite a recent increase in the study of abiotic stresses (salt and drought tolerance in *Brassica* [86, 87] and *Diplotaxis* [88]), and characterisation of plants for improving photorespiratory activities (reported in *Diplotaxis tenuifolia, D. muralis* (L.) DC., *D. erucoides* (L.) DC., and *Moricandia arvensis* (L.) DC., which are characterised as C3–C4 intermediate species [89, 90]), there is still work to be done, especially for minor crops and wild relatives, to increase their use in sustainable agriculture.

### Key areas for *ex situ* conservation

CWRs from the *Brassica* genus that are native to Europe and related to human food were included on a high-priority list for threat assessment [45]. Additionally, other Brassicaceae genera (*Armoracia, Barbarea, Camelina, Crambe, Diplotaxis, Eruca, Isatis, Lepidium, Raphanus, Rorippa, Sinapidendron*, and *Sinapis*) were also considered for the European Red List because of their importance to human and animal food. However, conservation assessments are urgently needed, since almost 80% of the wild Brassicaceae are Data Deficient according to the IUCN Red List [91] or not assessed for their global threatened status (Supplementary Data D1), including 38 taxa that are new CWRs potentially cross-compatible with crops.

Based on the results presented here, the Mediterranean basin and the Middle East are 2 hotspots for wild and endemic Brassicaceae species (Fig. 3). The areas are 2 of the centres of origin highlighted by Vavilov and both are hotspots for plant biodiversity [48, 92]. For example, *Brassica oleracea* is thought to have been domesticated in the Eastern Mediterranean [93]. On the other hand, some studies propose Central Asia as the origin of domestication for *Brassica rapa* but do not dismiss the possibility of multiple origins of domestication [94]. Many species are endemic to China, and several regions in Asia should also be considered for exploring key traits (Fig. 3B).

This study contributes to determining conservation gaps in the Brassicaceae family, identifying the need for further collection and conservation of wild species. We have compiled information available of 1,242 Brassicaceae species, emphasising the gaps in genetic sequence data (more than 700 spp. lack this information), conservation status (only 400 are conserved *ex situ* and fewer than 300 are assessed in the IUCN Red List), and trait characterisation to promote their use as cross-compatible CWRs. The Mediterranean region has been described as a potential hotspot of threatened and endemic Brassicaceae species that have yet to be conserved. Additionally, we also have revealed gaps in understanding and evaluating CWRs for this important family (more than 500 species not characterised). Using phylogenetic distances, we proposed 103 new potential CWRs, of which 72 are already conserved *ex situ*. Once conserved, these CWRs should be characterised physiologically and genetically, requiring the sequencing of more markers (nuclear and plastid) and ploidy studies. This will facilitate their use in future breeding programmes.

## Materials and Methods

### Genetic data and phylogenetic analysis

Cultivated Brassicaceae species were identified using Annex I on the International Treaty on plant genetic resources for food and agriculture [95] and scientific literature, gathering a total of 22 major and minor crops from 15 genera. All wild species from the same genera as the cultivated Brassicaceae crops were added to the database. Similarly, using the accepted CWR lists based on gene pool and taxon group classifications [20, 55], a further 14 new genera were included, obtaining a final target list of 29 genera and 1,242 taxa.

We used available data of cross-compatibility between species and phylogenetic reconstructions to estimate phylogenetic distances between species and differences in ploidy levels to estimate the potential of each pair of species to be cross-compatible [24]. We used wild species with successful conventional crosses reported in the literature [42, 43, 55] (represented with a “Y” and “Y*” in Table 1) to predict new potential cross-compatible CWR with their respective crop. We built a phylogenetic tree where pairwise phylogenetic distances between the tips were estimated using the patristic method with the *adephylo* package [96] (v.1.1.13). The phylogenetic trees were transformed to ultrametric, and the distance of the branches was standardised to “1.0” from the root. A threshold was established within the range of phylogenetic distance from a crop taxon to a known cross-compatible wild species.

DNA sequence data were compiled from NCBI [97] (accessed in November 2022) using several markers (*rbc*L, *mat*K, ITS, and *trn*L-F) selected for their higher number of sequences available for the Brassicaceae family (Supplementary Fig. S2). Chromosome numbers and ploidy levels were collected from the Wild Germplasm of *Brassica* [43] (Part II: Chromosome number), Brassibase [7] (accessed in November 2022), the Plant DNA C-values database [98] (accessed in November 2022), and plant CCDB database [99] (accessed in December 2022). DNA records were cleaned and analysed in R [100] (version 4.2.1), using *tidyverse* [101], *seqinr* [102], and *ape* [103] packages. The sequences were aligned using MAFFT [104] (v7.505) and cleaned with *trimAl* [105], applying the parameters *resoverlap* 0.70 and *resoverlap seqoverlap* 0.75. Alignments were edited to remove sequences with large gaps and samples with missing data (80% or higher) using AMAS [106]. The phylogenetic tree was built using the maximum likelihood criterion as implemented in IQ-TREE [107] (v. 2.0.6) using the substitution model selected in MFP (*ModelFinder Plus*), which was GTR+G+I. The phylogenetic trees represented in this study included only 1 sequence per species, which corresponded with the longest sequence available. We also discarded any sequence not clustering with the remaining sequences of the same species in a preliminary analysis. We used *Aethionema thomasianum* J. Gay as an outgroup. The bootstrap was set up with 1,000 replicates and an ultrametric tree calculated with the *phangorn* package [108]. To compare and show the 2 phylogenetic trees, we used the cophylo function from the *phytools* package [109] (v.1.2.0), using *ggplot2* [110] and *magick* [111] to collate the ploidy figures and the trees.

### Trait characterisation

Agronomic and physiological traits were obtained from the literature and from several databases such as USDA GRIN global [55] (accessed in December 2022) and the Harlan and De Wet CWR inventory [56] (accessed in December 2022) for all the CWRs that had information available. Additionally, 50 seed and plant traits (Supplementary Data D4) were gathered from the TRY database [26]. This database includes specific traits and plant characterisation that have been published or reported in other databases, research articles, or unpublished data.

### Distribution and conservation data

The distribution and accepted scientific names were downloaded and matched from World Checklist of Vascular Plants [60] (version 9, accessed in February 2022) for all taxa. The distribution of introduced species was not included to focus on the native distribution of wild species. For the geographical distribution, we used the Biodiversity Information Standards (before known as the Taxonomic Databases Working Group, TDWG) level 3. We used the IUCN Red List [91] (accessed in September 2022) and the ThreatSearch tool from Botanic Gardens Conservation International (BCGI [112], accessed in September 2022) to assess the global threat status of the Brassicaceae species. Similarly, global records of *ex situ* collections were gathered using Genesys [113] (data accessed through Genesys in November 2022 via R package *genesysr* [114]) and the Millennium Seed Bank Partnership database [115] (accessed in September 2022). The conservation status for the 1,242 species was extracted using *rredlist* package [116]. The analysis of the data for this section was performed in R [100] (v. 4.2.1) unless otherwise specified, using the following R packages: to curate, visualise, and analyse the data, we used *cowplot* [117] (v.1.1.1), *data.table* [118] (v1.14.8), *geojson* [119] (v.0.3.5), *sf* [120] (v.1.0.14), and *tidyverse* [101] (v2.0.0).

## Supplementary Material

giae050_GIGA-D-23-00376_Original_Submission

giae050_GIGA-D-23-00376_Revision_1

giae050_GIGA-D-23-00376_Revision_2

giae050_Response_to_Reviewer_Comments_Original_Submission

giae050_Response_to_Reviewer_Comments_Revision_1

giae050_Reviewer_1_Report_Original_Submission Chrystian C. Sosa -- 2/19/2024 Reviewed

giae050_Reviewer_1_Report_Revision_1 Chrystian C. Sosa -- 4/30/2024 Reviewed

giae050_Reviewer_2_Report_Original_Submission MAKENZIE Mabry -- 2/23/2024 Reviewed

giae050_Reviewer_3_Report_Original_Submission Lorenzo Maggioni -- 2/28/2024 Reviewed

giae050_Supplemental_File

## Data Availability

The sequence data used in this study and the script for curating the data are available in the *GigaScience* database, GigaDB [121]. Lists of NCBI accession numbers and taxa are also available via GigaDB (file names: “rbcl_id_spp.csv,” “its_id_sp.csv,” “matk_id_spp.csv,” “trn_id_spp.csv”). Additional Supplementary Data are available via Figshare [122]. These include a database with new CWRs, authorships of the taxa, and extra information of the species (D1), Global distribution (D1), current classification of CWRs (D2), Chromosome numbers (D3) and Traits (D4) for the species available, and Additional files (Figures S1, S2 and Table S1).

## Additional Files

**Supplementary Fig. S1**. Phylogenetic trees from each marker evaluated, (a) matK, (b) ITS, (c) rbcL, and (d) trnL-F.

**Supplementary Fig. S2**. Proportion of number of entries in the NCBI for 9 markers in the Brassicaceae family.

**Supplementary Table S1**. Classification of Brassicaceae wild species as CWRs combining 2 global databases.

## Abbreviations

CWR: crop wild relative; GP: gene pool; NCBI: National Center for Biotechnology Information; PD: phylogenetic distance; TG: taxon group.
