# Supplementary material for: Current status of global conservation and characterisation of wild and cultivated Brassicaceae genetic resources
Source: Gigascience. 2024 Aug 7;13:giae050. doi: 10.1093/gigascience/giae050 (PMC11304946; doi:10.1093/gigascience/giae050)
Supplement: giae050_GIGA-D-23-00376_Revision_2 [file giae050_giga-d-23-00376_revision_2.pdf]

GigaScience

Current status of global conservation and characterisation of wild and cultivated Brassicaceae genetic resources

--Manuscript Draft--

**Manuscript Number:**	GIGA-D-23-00376R2
**Full Title:**	Current status of global conservation and characterisation of wild and cultivated Brassicaceae genetic resources
**Article Type:**	Research
**Funding Information:**	
**Abstract:**	Background<br>The economic importance of the globally distributed Brassicaceae family resides in the large diversity of crops within the family, and the substantial variety of agronomic and functional traits they possess. We reviewed the current classifications of crop wild relatives (CWRs) in the Brassicaceae family with the aim of identifying new potential cross-compatible species from a total of 1,242 species using phylogenetic approaches.<br>Results<br>In general, cross-compatibility data between wild species and crops, and phenotype and genotype characterisation data, were available for major crops but very limited for minor crops, restricting the identification of new potential CWRs. Around 70% of wild Brassicaceae did not have genetic sequence data available in public repositories, and only 40% had chromosome counts published. Using phylogenetic distances, we propose 103 new potential CWRs for this family, which we recommend as priorities for cross compatibility tests with crops and for phenotypic characterisation, including 71 newly identified CWRs for 10 minor crops. From the total species used in this study, more than half had no records of being in ex situ conservation and 80% were not assessed for their conservation status of were data deficient (IUCN Red List Assessments).<br>Conclusions<br>Great efforts are needed on ex situ conservation to have accessible material for characterising and evaluating the species for future breeding programmes. We identified the Mediterranean region as one key conservation area for wild Brassicaceae species, with great numbers of endemic and threaten species. Conservation assessments are urgently needed to evaluate most of these wild Brassicaceae.
**Corresponding Author:**	Elena Castillo-Lorenzo<br>Royal Botanic Gardens Kew<br>Haywards Heath, England UNITED KINGDOM
**Corresponding Author Secondary Information:**	
**Corresponding Author's Institution:**	Royal Botanic Gardens Kew
**Corresponding Author's Secondary Institution:**	
**First Author:**	Elena Castillo-Lorenzo
**First Author Secondary Information:**	
**Order of Authors:**	Elena Castillo-Lorenzo
	Elinor Breman
	Pablo Gómez Barreiro
	Juan Viruel
**Order of Authors Secondary Information:**	
**Response to Reviewers:**	Dear Editor,<br>I have submitted the last version of the manuscript in Word. With the following changes<br>1- I have added the citations and text for the Supplemetary data in GigaDB and Figshare.

Powered by Editorial Manager® and ProduXion Manager® from Aries Systems Corporation

	2- I have removed the url links through the text and placed them only on the references<br>3- I included the ORCID number in the tittle page.<br>4- The abstract is now structured as Background, Results and Conclusions<br>5- I have re-order the last sections after the discussion section and placed the references at the very end<br>6- I removed the highlighted text<br>7- the uploaded document is in Word (.doc) format<br>8- I think all the references and style is according to the instructions to authors stated on the website.<br>I have added a sentence to the Acknowledgments section, there is a project of crop wild relatives that will help paying for the open access fees of this manuscript and I have acknowledged them. I hope this is okay.<br><br>Thank you very much!<br>Best,<br>Elena
**Additional Information:**	
**Question**	**Response**
Are you submitting this manuscript to a special series or article collection?	No
**Experimental design and statistics**<br><br>Full details of the experimental design and statistical methods used should be given in the Methods section, as detailed in our Minimum Standards Reporting Checklist. Information essential to interpreting the data presented should be made available in the figure legends.<br><br>Have you included all the information requested in your manuscript?	Yes
**Resources**<br><br>A description of all resources used, including antibodies, cell lines, animals and software tools, with enough information to allow them to be uniquely identified, should be included in the Methods section. Authors are strongly encouraged to cite Research Resource Identifiers (RRIDs) for antibodies, model organisms and tools, where possible.<br><br>Have you included the information requested as detailed in our Minimum Standards Reporting Checklist?	Yes

**Availability of data and materials**<br><br>All datasets and code on which the conclusions of the paper rely must be either included in your submission or deposited in publicly available repositories (where available and ethically appropriate), referencing such data using a unique identifier in the references and in the "Availability of Data and Materials" section of your manuscript.<br><br>Have you have met the above requirement as detailed in our Minimum Standards Reporting Checklist?	Yes

Powered by Editorial Manager® and ProduXion Manager® from Aries Systems Corporation

Manuscript

Click here to access/download;Manuscript;BrassicaceaeCWRs_for

Current status of global conservation and characterisation of wild and cultivated Brassicaceae genetic resources

Elena Castillo-Lorenzo[α+], e.castillolorenzo@kew.org
ORCID - 0000-0001-6123-0289

Elinor Breman[α], e.breman@kew.org
ORCID- 0000-0001-9834-5186

Pablo Gómez Barreiro[α], p.gomez@kew.org
ORCID - 0000-0002-3140-3326

Juan Viruel[β] j.viruel@kew.org
ORCID - 0000-0001-5658-8411

[α] Royal Botanic Gardens, Kew, Wakehurst, Ardingly, Haywards Heath, West Sussex, RH17 6TN, UK

[β] Royal Botanic Gardens, Kew, Richmond, Surrey TW9 3AE, UK

[+] Corresponding author

Abstract

Background

The economic importance of the globally distributed Brassicaceae family resides in the large diversity of crops within the family, and the substantial variety of agronomic and functional traits they possess. We reviewed the current classifications of crop wild relatives (CWRs) in the Brassicaceae family with the aim of identifying new potential cross-compatible species from a total of 1,242 species using phylogenetic approaches.

Results

In general, cross-compatibility data between wild species and crops, and phenotype and genotype characterisation data, were available for major crops but very limited for minor crops, restricting the identification of new potential CWRs. Around 70% of wild Brassicaceae did not have genetic sequence data available in public repositories, and only 40% had chromosome counts published. Using phylogenetic distances, we propose 103 new potential CWRs for this family, which we recommend as priorities for cross compatibility tests with crops and for phenotypic characterisation, including 71 newly identified CWRs for 10 minor crops. From the total species used in this study, more than half had no records of being in *ex situ* conservation and 80% were not assessed for their conservation status or were data deficient (IUCN Red List Assessments).

Conclusions

Great efforts are needed on *ex situ* conservation to have accessible material for characterising and evaluating the species for future breeding programmes. We identified the Mediterranean region as one key conservation area for wild Brassicaceae species, with great numbers of endemic and threatened species. Conservation assessments are urgently needed to evaluate most of these wild Brassicaceae.

Keywords

Crop wild relatives, cross-compatible, phylogenetic distances, plant conservation, cultivated Brassicaceae, breeding.

Introduction

Improving crops to face biotic and abiotic stresses, and to enhance their nutritional value is essential for ensuring global food security[1]. Ongoing biodiversity loss or decline can have a detrimental effect on future food security, natural diversity provides resources to overcome challenges to food production such as environmental changes, pests and diseases or limited land availability[2]. CWRs hold a wealth of genetic diversity which can be used to improve and help adapt traditional crops to succeed under environmental changes, making them of paramount importance for research and conservation[3, 4]. For example, CWRs have recently been used to transfer key traits to common crops in breeding programmes, such as tolerance and resilience to diseases and abiotic stresses such as salt or drought conditions[5] (and references therein). Previous studies have focused on major crops such as pulses, cereals and forages, and their respective CWRs[6], and relatively little work has been done on oil crops, vegetables or fruits, and minor crops, such as those found in the Brassicaceae family. To date, this family has more than 300 accepted genera and around 4,000 species[7, 8], and possess a wide array of genetic diversity. It owes its economic importance to the widespread use of edible root crops, vegetables and oilseeds[9].

Due to the importance of the family, several phylogenetic studies have aimed to unravel the systematics within Brassicaceae, and several taxonomic circumscriptions have been proposed to divide the family in tribes and to recognise relationships between species[10-14]. In a revision of the family, Al-Shehbaz et al.,[15] divided Brassicaceae in 25 tribes but highlighted that further revisions might be required due to the large number of species (400) and genera (100) not yet sequenced. The tribe Brassiceae, formed by eight clades, is the most studied

because it includes the *Brassica* complex or U's triangle[16, 17], which is formed by six globally important species of the genus that share three core genomes, termed A, B, and C, that have evolved independently[18]. These studies are useful to understand the phylogenetic relationships between wild and cultivated species, but also to estimate cross-compatibility between them to enable future breeding[19, 20]. However, further sampling efforts and sequence data are still required.

Classification of CWRs

Transfer of genes between CWRs and crops can be challenging because of reproductive barriers between each pair of species[21]. Thus, knowing the level of cross-compatibility between CWRs and their respective crop(s) is key to transferring desirable traits using traditional breeding approaches. There are three different methods to classify CWRs, each aiming to identify the cross-compatibility of a CWR to its respective crop. The most important and accurate classification was proposed by Harlan and de Wet [22], which relies on actual crossing data between a crop and a wild species. They classified CWRs in gene pools (GP): GP1 corresponds with cross-compatible individuals of the same species as the crop, GP2 represents a successful cross-pollination between a CWR and a crop, and GP3 is generally not compatible or results in sterile hybrids. However, producing this type of data is challenging, requiring living samples and investment. Thus, other classifications have been proposed when these resources are lacking. The taxon group (TG) classification[23] aims to estimate evolutionary relatedness based on taxonomic and hierarchical relationships. Four taxon groups were proposed to classify CWRs in the same genus as the crop to be cross-compatible; however, taxonomic circumscriptions do not necessarily reflect phylogenetic relationships. More recently, Viruel et al.,[24] proposed the use of the phylogenetic distances to estimate cross-compatibility, where shorter phylogenetic distances between species equates to a greater

possibility for them to be cross-compatible. This is a useful tool, especially when there is no information available for the GP classification.

Characterisation of Brassicaceae species

Brassicaceae possesses a wide variety of crops and cultivated species, some of the most important ones are within the *Brassica* genus. Cabbage, broccoli (*B. oleracea* L.), turnip (*B. rapa* L.), rapeseed (*B. napus* L.) and mustard (*B. juncea* L. Czern.) are the main crops and most economically important within the family. There are breeding needs in agriculture that target different traits of these crops, such as resistance to biotic diseases, adaptation or tolerance to abiotic stresses and improving or enhancing agronomic and functional traits[25].

Efforts to characterise CWRs physio- and phenotypically have increased in recent years describing traits of interest. However, these often focus on plant growth, leaf characterisation and composition, dispersal syndrome and a few traits related to seeds such as germination, storage behaviour and mass (TRY database[26]). As a result, there is less information on their tolerance to biotic and abiotic stresses compared to other key traits. Cultivated Brassicaceae are affected by biotic and abiotic hazards that cause loss of yield and poor performance in the field (especially pests[27] and diseases[28, 29]). CWRs of the Brassicaceae family are known to host desirable agronomic traits (compiled in[25, 30-32]), and abiotic stress tolerance such as to drought and salinity[33-35]. Different successful crosses have been performed to transfer some of these traits between *Brassica* crops and wild species[36-40]. The potential advantages of these crosses area not limited to agronomic traits and include health related applications such as, a CWR of broccoli, *Brassica villosa* Biv., has been used to increase anti-cancer compounds in a new variety[41].

Understanding the cross-compatibility between a CWR and a crop is essential for identifying the breeding techniques required to incorporate traits from wild species into the cultivar. However, publications of successful sexual crosses between crops and CWRs of Brassicaceae

family are very limited (reviews mainly on *Brassica* genus[39, 42, 43]), which could be due to the complexity of the process[31] and the lack of knowledge and characterisation of Brassicaceae CWRs. Therefore, it is critical to characterise and understand the cross-compatibility between CWRs and crops of Brassicaceae, as well as to preserve potential CWRs to facilitate their accessibility and conservation for future sustainable use.

Conservation and accessibility

Many CWRs are threatened with extinction by a range of factors such as land use and environmental changes, overexploitation, or invasive species[44]. Kell et al.,[45] urged for conserving at least 78% of the known CWRs in Europe and suggested increasing the use of data on population distribution, trends and size, as well as threat status, to design effective conservation plans. This would require information on the global distribution of wild genetic resources and their current *ex situ* representation in genebanks. Previous studies have identified gaps for specific areas (e.g., Indonesia[46], USA[47], Middle East[48], Europe[49]), or for specific crops (e.g., Hordeum[50], Capsicum[51], *Solanum*[52]) and landraces[53]. In the last decade, there has been an increase in *ex situ* conservation of CWRs[4, 6, 20, 54]. However, there are CWRs of major and minor crops yet to be conserved, especially from the Brassicaceae family which contains 70 priority CWRs for 17 crops[20]. Although there are scientific publications on *Brassica* crops, varieties, landraces and wild relatives[30, 43], there has been very little focus on the characterisation and conservation of minor crops, or on the evolutionary relationships between crops and wild Brassicaceae.

In this study we aim to review the current classification of CWRs in Brassicaceae, to identify new CWRs potentially cross-compatible with cultivated Brassicaceae estimated by phylogenetic distance; and to describe the current geographic distribution and ex situ conservation status of CWRs in Brassicaceae.

Results

Gaps in genetic sequence data and phylogeny

We have obtained DNA sequence data for 30% of the species (348 spp. out of a total 1,242 spp.) for four DNA regions: *rbc*L (175 spp.), *mat*K (162 spp.), ITS (241 spp.), and *trn*LF (277 spp). Phylogenetic trees were built independently for each four genetic markers (Supplementary material Fig. S1) and the phylogenetic trees with the highest resolution and bootstrap support were obtained using *mat*K data (with 131 taxa from the total of 162) and ITS data (214 taxa from the total of 241). In both phylogenetic trees, the tribes and groups were clearly divided in concordance with those defined in previous studies. A comparison of both phylogenetic trees was built to identify similarities and disparities between them (containing 85 common species, Fig. 1). Although the phylogenetic tree for ITS marker had more DNA sequence data, the genera *Armoracia, Barbarea, Crambe* and *Nasturtium* were only present in the plastid marker *mat*K (Fig. 1). On the other hand, there were more DNA sequences available from the genera *Physaria* and *Isatis* for the ITS marker.

Identification of potential cross-compatible CWRs

In this study we distinguished successful conventional crosses of CWRs and crops that will produce hybrids, from crosses that will require biotechnology techniques (e.g., in vitro culture, embryo or ovary rescue). A summary of previous GP and TG classifications for CWRs in Brassicaceae including 265 taxa (20% of the total 1,242) is listed in Supplementary material Table S1. However, there were some wild species assigned as GP2 where no evidence of successful conventional cross pollination has been published. For example, for *B. elongata* Ehrh. as a GP2 of turnip (*B. rapa*, Table 1 and Supplementary material Table S1) with no information of crosses between them. Thus, to apply phylogenetic distances (PD) as a proxy for cross-compatibility, we only use as a reference CWRs with successful sexual crosses with the crop. The PD thresholds were specific for each crop and ranged from 0 (the closest species

in the phylogenetic tree) up to 0.19 (the furthest cross-compatible CWR, Table 1) to predict potential cross-compatible CWRs. For example, white mustard, *Sinapis alba* L., and the wild species *Kremeriella cordylocarpus* (Coss. & Durieu) Maire, are suggested to be cross-compatible (Table 1, PD = 0.0096), because pairwise phylogenetic distances were lower than other known CWR with reported successful crosses with the crop (Table 1, PD < 0.0125). Using this method based on phylogenetic distances, we propose 103 new potential CWRs (Table. 1, see Supplementary data for more details) to be cross-compatible with 18 cultivated species.

The potential cross-compatibility CWRs estimated using phylogenetic distances will need to be revised considering ploidy level variation, because Brassicaceae species have a large variation in chromosome numbers, from $2n$=150 (*Crambe gordjaginii* Sprygin & Popov) to $2n$=8 for some *Physaria* species (Fig.1, for detailed information see Supplementary data), and crosses between the same ploidy levels are recommended when possible. For example, using phylogenetic distances, we estimated that *Brassica gravinae* Ten. ($2n$=20) is likely cross-compatible with turnip (*B. rapa*, $2n$= 10 and 20 between others). We recommend using the same cytotype forms ($2n$=20) to attempt crossing them. However, only 40% of the wild Brassicaceae species on this study had ploidy level or chromosome number information available (474 taxa) of which 122 are in ITS and 93 in *mat*K phylogenetic tree (55 in common, Fig. 1).

Major crops

All the major crops listed in Table 1 were present in both phylogenetic trees and had successful conventional crosses reported that can confirm the cross-compatibility between some species. Since major crops had more information published, we identified potential CWRs based on the phylogenetic distances, including new genera and species not previously suggested that had

shorter distances (Table 1) than the cross-compatible CWRs already identified (Supplementary material Table S1).

Minor crops

In general, there was very little information regarding successful conventional crosses between wild species and minor cultivated crop (perennial wall rocket, *Diplotaxis tenuifolia* (L.) DC., was the only exception). For some taxa there were not enough sequences (*Barbarea* genus) or no DNA sequence data (e.g., cultivated *Crambe*), in others the problem was the lack of information on the cross-compatibility as a reference on the phylogenetic tree (e.g., *Eutrema japonicum* (Miq.) Koidz., had many wild species with DNA sequence data, but lacked referenced species as confirmed cross-compatible).

Traits for breeding

We identified gaps in the characterisation of the wild Brassicaceae species included in this study by compiling information in different databases to describe the most and least explored and characterised species. USDA-GRIN Global database on CWR[55] and Harlan and de Wet CWR inventory[56] compile traits of CWR and hold information for 14 cultivated Brassicaceae taxa (Supplementary data) and 171 wild species related to them. Biotic traits are the most studied, followed by fertility traits, the combination of both represents 74% of the available data. The remaining 26% are abiotic and agronomic traits (Fig 2a). Additionally, TRY database[26] shows more than 7,000 entries for wild Brassicaceae and there is information on potential traits of 599 Brassicaceae species. The main traits captured focus on morphology and physiology (e.g., plant growth, flowering time, dispersal syndrome, Fig. 2a). The genus with the largest number of traits recorded and published is *Lepidium* (416), followed by *Brassica* (347, Fig. 2b). However, the top five species that were the most characterised, with more traits

identified, are from the *Brassica* genus (five of the six species that form the U's triangle, Fig. 2b).

Geographical conservation gaps of Brassicaceae

The distribution of all 1,242 studied populations confirms the global presence of wild Brassicaceae species, some of them are widely cultivated (i.e., *Brassica rapa, B. juncea, Raphanus raphanistrum* subsp. *sativus* (L.) Domin) or introduced. However, introduced species were removed from the analysis to focus on the native distribution of wild Brassicaceae. For the geographical distribution we used TDWG (Biodiversity Information Standards) level 3, and the three regions with the greatest number of native taxa are Turkey (160 species), Spain (147) and Morocco (135, Fig. 3a). There are 787 species yet to be conserved *ex situ* (i.e., no records available on global databases) and more than 200 that are underrepresented (less than 5 populations conserved *ex situ*, Supplementary data). The greatest number of taxa missing from *ex situ* collections occur in Turkey (46), Kazakhstan (32) and Colorado regions (26, Fig. 3b).

Conservation status has been evaluated for only 440 species, of which ca. 30% are considered threatened (119 threatened of which 110 are also endemic). The highest number of threatened taxa were found in the Canary Islands (14), peninsular Spain (10), Colorado (11) and Cape Verde regions (9, Fig. 3c, see Supplementary data for more details). Half of the taxa in the database represent single region endemics (667 species). The greatest number of endemic species are found in Turkey (49), peninsular Spain (39) and Colorado regions (36, Fig. 3d, see Supplementary data for more details).

The geographical distribution of the new 103 proposed as cross-compatible CWRs has been defined (Fig. 4) and we observed that almost 70% of these species are not well represented in *ex situ* conservation or not represented at all (36%, Supplementary data). Unfortunately, more

than 70% of them have not being globally evaluated for their conservation status and the level of threat of their populations is unknown (Supplementary data).

Discussion

Identification of new potential CWRs in Brassicaceae from available DNA data

Various molecular markers have been used to reconstruct phylogenetic trees to distinguish between species and identify clades in Brassicaceae, the most frequently used are *rbc*L, *mat*K, ITS and *trn*L_F. In general, *rbc*L is considered a slow evolving gene, *mat*K is intermediate and ITS and *trn*L_F are evolving relatively faster[57]. The choice of markers used is based on the desirable outcome, for example a combination of two markers such as *rbc*L and *mat*K has been suggested to build phylogenetic trees and identify species[58]. In the present study, we compared one nuclear marker (ITS) and one plastid marker (*mat*K, Fig. 1). A more comprehensive phylogeny was recently published using larger Brassicaceae dataset (one species per genus[11]). The cultivated Brassicaceae were well spread around the supertribes Camelinodae (I) and Brassicodae (II) of the phylogenetic tree[11, 13], with all major crops present in Brassicodae (II) (Supplementary material Fig. S1). In our study, both phylogenetic trees (ITS and *mat*K) were congruent regarding the major clusters or groups formed, however there were a few discrepancies.

- The genus *Eutrema* was split in the phylogenetic tree ITS in two clades, one containing most *Eutrema* species and a second with *E. violifolium* (H.Lév.) Al-Shehbaz & Warwick, *E. yungshunense* (W.T.Wang) Al-Shehbaz & Warwick, *E. xingshanense* (Z.E.Chao, Z.L.Ning & X.W.Hu) G.Q.Hao, Al-Shehbaz & J.Quan Liu and *E. grandiflorum* (Al-Shehbaz) Al-Shehbaz & Warwick, grouped to a clade formed by the genus *Orychophragmus*. This is in agreement with other phylogenetic trees[59] where the genus *Eutrema* was split due to the geographical distribution of the species (all of

them occur only in Central China) and were clustered with *Orychophragmus* taxa, which are mostly distributed in China. However, the phylogenetic tree reconstructed with *mat*K resolved *Eutrema* species in a monophyletic clade, although aforementioned four species formed a subclade within the genus. This could reflect a different evolutionary history between nuclear and plastid markers (reflected by larger phylogenetic distances, Fig. 1), and those four species are likely to be non-compatible with wasabi crop (*Eutrema japonicum*), but further research is needed for this unexplored crop.

- The *Erucastrum* genus was scattered around the Brassiceae tribe in the phylogenetic tree *mat*K (Fig. 1) with *Erucastrum* species placed in both the Rapa/Oleracea and in the Nigra clades, as reported in previous phylogenies[17, 32]. In general, only two species are well studied in this genus, *E. abyssinicum* (A.Rich.) O.E.Schulz and *E. gallicum* (Willd.) O.E.Schulz, (the latter is widely distributed in USA, Europe and some areas of Asia[60]), and both were present in the two phylogenetic trees, *mat*K and ITS. The division in the *mat*K marker could be due to the distribution of the species, however, more populations should be investigated to verify this. Additionally, some of them are edible[32] and may have been subjected to some type of selection, a potential further reason for their distribution across clades.

Chromosome numbers also play an important role to estimate cross-compatibility between species. The evolution of Brassicaceae species seems to be driven by whole genome duplication events and polyploidy[61]. These polyploidy events are species and lineage-specific and can affect the relationships of the species in the phylogeny. Polyploidy is present especially in the Brassiceae tribe[62], for example, polysomaty or mixoploidy (having cells with different numbers of chromosomes in different tissues or cells) has been reported in *Brassica* and *Raphanus* genera[63]. There is a large disparity in chromosome numbers within

the family[61, 62] (Supplementary data), making ploidy data critical for the identification of cross-compatible species in addition to the phylogenetic distances. For example, variation in ploidy levels could be the reason why *Brassica oleracea*, a progenitor of *B. carinata* A.Braun, has few successful crosses and very low rates of hybrids produced[42].

Harlan and de Wet's classification[22] of CWRs enables the identification of potentially compatible wild species. They defined secondary gene pool (GP2) species as those that will be able to transfer genes by conventional crosses, with some possible barriers or lower success rates. They also suggested that gene pools could be separated based on different ploidy levels, but to our knowledge this approach has not been carried out, which could be challenging when species exhibit multiple ploidy levels. The literature and databases contain a mixture of 'secondary' and 'tertiary' (not compatible or resulting in sterile hybrids) CWRs, where no crosses are found in the literature, or with very limited success of crossing (Table 1), or even where biotechnology techniques (e.g., embryo rescue, ovary rescue, somatic hybrids) were required to obtain hybrids. Due to the complexity of the Brassicaceae family and the mixed classifications, it is more difficult for pre-breeders to use some of these wild genetic resources, because in many cases previous classifications were not validated by crosses. Thus, a detailed review of CWRs lists corroborated with data from crosses is urgently needed to clarify our current knowledge of Brassicaceae CWRs. In this study we compiled information of successful crosses, chromosome numbers and phylogenetic distances (Fig. 1) to update the classification of CWRs in Brassicaceae and to identify new CWRs that are potentially cross-compatible with crops (Table 1). These newly identify CWRs, using phylogenetic distances, will require characterisation and evaluation for crossability with the crop.

Major crops

As expected, there were more data and publications for well-known crops (*Brassica* U's triangle, *Eruca*, *Sinapis*, *Raphanus*) and their CWRs than for minor crops, especially regarding

breeding and agronomic traits and ploidy level. Despite the *Brassica* genus comprising most of the major crops, the phylogenetic relationships between species are still far from understood. Brassiceae is a polyploid tribe[64] and this is a challenge for taxonomists and geneticists and more investigation is needed to resolve taxonomic issues, and fully understand the cross-compatibility between species.

There are some incongruences on the cross-compatibility in the literature within the *Brassica* U's triangle. For example, despite being in different clades (Nigra and Oleracea respectively[17], Fig. 1) and having longer phylogenetic distances, *B. nigra* and *B. oleracea* are classified as secondary CWRs to each other due to successful crosses between them, but only when the *B. nigra* was used as the female parent[42]. In general, most of the crosses that were successful within this tribe had the cultivated species as the female donor[42]. Another successful interspecies sexual hybridisation was published by Kumar et al.[37], between *B. rapa* and *B. fruticulosa* (*B. rapa* being the female parent) and this technique has been used by other researchers as a bridge to transfer resistance genes from *B. fruticulosa* to *B. juncea*[40]. *B. rapa* and *B. juncea* are classified as GP3, however successful crosses between them have been reported[65, 66]. On the other hand, *B. oleracea* is one of the species from which *B. napus* originated, but the crosses between them produced a very low number of hybrids[42]. However, *B. oleracea* has been successfully crossed (by conventional reproduction), with *B. cretica* Lam., *B. incana* Ten., *B. macrocarpa* Guss., *B. montana* Pourr., and *B. villosa* Biv.[67]. Nonetheless, further research is required to improve the success of gene transfer for this species.

There are also successful intergeneric crosses involving the *Brassica* genus such as attempts to cross with *Orychophragmus violaceous*[68, 69] even though the species is distant in the phylogenetic tree (Table 1). *Diplotaxis tenuifolia* and *Erucastrum gallicum* were also able to produce hybrids when crossing with *Brassica* species, although in some cases these were only

successful when *Brassica* was the female parent[70, 71]. Intergeneric crosses were also possible with *Raphanus* species and *Eruca vesicaria* (L.) Cav., but with low success and in some cases biotechnology techniques were required to overcome cross-compatibility barriers[72-74].

Minor crops

Minor or less common cultivated species such as hedge mustard, cress cultivars or Abyssinian kale are less widely cultivated and thus, less information is available for them. *Crambe hispanica* subsp. *abyssinica* (Hochst. ex R.E.Fr.) Prina, was not included in the phylogenetic tree due to lack of genetic sequence information. Similarly, characterisation and information about interesting traits within wild species of these minor crops are lacking in the literature. In some cases, there is an issue of self-incompatibility or sterile plants (*Armoracia*[75]) which makes the breeding process more complex. Due to the limited information about their ploidy levels, it is complicated to identify potential candidates to be cross-compatible with cultivated species. This is the case in the genus *Diplotaxis*, which possess dysploidy (an organism that has an increased or decreased number of chromosomes, by one or more, than the original[76]). However, these CWRs could hide a wide genetic diversity and future evaluation of their adaptation and traits would be useful. For example, the *Barbarea* genus is considered a great source of plant defence compounds within the family[77], and some species show resistance to several biotic stresses (mildew, nematodes and thrips[78]). In other genera, medicinal compounds have been reported (*Isatis*[79], *Nasturtium*[80, 81] and *Sisymbrium*[82]), and the effect of different environmental conditions have been evaluated (*Isatis*[83], *Nasturtium*[84] and *Rorippa*[85]).

Probably due to the novelty of some of these crops, very little has been done to improve their characteristics, but also few traits have been characterised to understand the requirements (if any at this stage) to cultivate these species and therefore, further investigation is needed,

especially to understand and improve their performance and adaptation. Using available data for Brassicaceae, we have identified around 103 new potentially cross-compatible CWRs (Table 1, see Supplementary data for more details) for 18 crops, although, in general more investigation is needed. More species will need to be sequenced and generate more data (e.g., physiological and phenotypic characterisation as well as acquiring knowledge of the ploidy level). This is key to understand the needs of the cultivated species and to identify CWRs with interesting traits. Confirming the cross-compatibility of the new potential CWRs with the same ploidy level is critical, in addition to generating more DNA sequencing data to complete the genetic characterisation of the family.

Cultivated Brassicaceae limitations

A detailed characterisation of plant species is fundamental to understand the limitations of cultivated species. Combining phenotypic and genotypic data will positively impact on improving and transferring traits to major and minor crops as reviewed by Katche et al.[39]. As for the compatibility data, phenotypic and genotypic characterisation is generally available for major crops and non-existent or rare for less well-known cultivated species (*Crambe*, *Nasturtium*, or *Diplotaxis*). The exception observed in Fig. 2b is for *Lepidium*, which was one of the top three genera with more species characterised for at least one trait, however this could be due to the large number of accepted species included in the genus (up to 262 spp.).

The most studied traits were those related to the morphology and phenology of the plant in addition to agronomic traits and biotic stress resistance[36, 40, 73]. Despite a recent increase in the study of abiotic stresses (salt and drought tolerance in *Brassica*[86, 87] and *Diplotaxis*[88]), and characterisation of plants for improving photorespiratory activities (reported in *Diplotaxis tenuifolia*, *D. muralis* (L.) DC., *D. erucoides* (L.) DC. and *Moricandia arvensis* (L.) DC., which are characterised as C3-C4 intermediate species[89, 90]), there is still

work to be done especially for minor crops and wild relatives to increase their use in sustainable agriculture.

Key areas for ex situ conservation

CWRs from the *Brassica* genus that are native to Europe and related to human food were included on a high priority list for threat assessment[45]. Additionally other Brassicaceae genera (*Armoracia, Barbarea, Camelina, Crambe, Diplotaxis, Eruca, Isatis, Lepidium, Raphanus, Rorippa, Sinapidendron and Sinapis*) were also considered for the European Red List because of their importance to human and animal food. However, conservation assessments are urgently needed, since almost 80% of the wild Brassicaceae are Data Deficient according to the IUCN Red List[91], or not assessed for their global threatened status (Supplementary data), including 38 taxa that are new CWRs potentially cross-compatible with crops.

Based on the results presented here, the Mediterranean basin and the Middle East are two hotspots for wild and endemic Brassicaceae species (Fig. 3). The areas are two of the centres of origin highlighted by Vavilov and both are hotspots for plant biodiversity[48, 92]. For example, *Brassica oleracea* is thought to have been domesticated in Eastern Mediterranean[93]. On the other hand, some studies propose Central Asia as the origin of domestication for *Brassica rapa*, but do not dismiss the possibility of multiple origins of domestication[94]. There are many species that are endemic to China and several regions in Asia that should also be considered for exploring key traits (Fig. 3b).

This study contributes to determining conservation gaps in the Brassicaceae family, identifying the need for further collection and conservation of wild species. We have compiled information available of 1,242 Brassicaceae species, emphasising the gaps in genetic sequence data (more than 700 spp. lack this information), conservation status (only 400 are conserved *ex situ* and less than 300 are assessed in the IUCN Red List) and trait characterisation to promote their use

as cross-compatible CWRs. The Mediterranean region has been described as a potential hotspot of threaten and endemic Brassicaceae species that have yet to be conserved. Additionally, we also have revealed gaps in understanding and evaluating CWRs for this important family (more than 500 species not characterised). Using phylogenetic distances, we proposed 103 new potential CWRs, of which 72 are already conserved *ex situ*. Once conserved, these CWRs should be characterised physiologically and genetically, requiring the sequencing of more markers (nuclear and plastid) and ploidy studies. This will facilitate their use in future breeding programmes.

Material and methods

Genetic data and phylogenetic analysis

Cultivated Brassicaceae species were identified using Annex I on the International Treaty on plant genetic resources for food and agriculture[95], and scientific literature, gathering a total of 22 major and minor crops, from 15 genera. All wild species from the same genera as the cultivated Brassicaceae crops were added to the database. Similarly, using the accepted CWRs lists based on gene pool and taxon group classifications[20, 55], a further 14 new genera were included, obtaining a final target list of 29 genera and 1,242 taxa.

We used available data of cross-compatibility between species and phylogenetic reconstructions to estimate phylogenetic distances between species and differences in ploidy levels to estimate the potential of each pair of species to be cross-compatible[24]. We used wild species with successful conventional crosses reported in the literature[42, 43, 55] (represented with a ‘Y’ and ‘Y*’ in Table 1) to predict new potential cross-compatible CWR with their respective crop. We built a phylogenetic tree where pairwise phylogenetic distances between the tips were estimated using the patristic method with the *adephylo* package[96] (v.1.1.13). The phylogenetic trees were transformed to ultrametic and the distance of the

branches were standardised to '1.0' from the root. A threshold was established within the range of phylogenetic distance from a crop taxon to a known cross-compatible wild species.

DNA sequence data were compiled from NCBI (National Center for Biotechnology Information[97] accessed on November 2022) using several markers (*rbc*L, *mat*K, ITS and *trn*LF) selected for their higher number of sequences available for the Brassicaceae family (Supplementary material Fig. S2). Chromosome numbers and ploidy levels were collected from the Wild Germplasm of *Brassica*[43] (Part II: Chromosome number), Brassibase[7] (accessed on November 2022), the Plant DNA C-values database[98] (accessed on November 2022) and plant CCDB database[99] (accessed on December 2022). DNA records were cleaned and analysed in R[100] (version 4.2.1), using *tidyverse*[101], *seqinr*[102] and *ape*[103] packages. The sequences were aligned using MAFFT[104] (v7.505) and cleaned with *trimAl*[105] applying the parameters *resoverlap* 0.70 and *resoverlap seqoverlap* 0.75. Alignments were edited to remove sequences with large gaps and samples with missing data (80% or higher) using AMAS[106]. The phylogenetic tree was built using the maximum likelihood criterion as implemented in IQ-TREE[107] (v. 2.0.6) using the substitution model selected in MFP (*ModelFinder Plus*), which was GTR+G+I. The phylogenetic trees represented in this study included only one sequence per species, which corresponded with the longest sequence available. We also discarded any sequence not clustering with the remaining sequences of the same species in a preliminary analysis. We used *Aethionema thomasianum* J. Gay as outgroup. The bootstrap was set up with 1,000 replicates and an ultrametric tree calculated with *phangorn* package[108]. To compare and show the two phylogenetic trees we used the cophylo function from the *phytools* package[109] (v.1.2.0), using *ggplot2*[110], and *magick*[111] to collate the ploidy figures and the trees.

Trait characterisation

Agronomic and physiological traits were obtained from the literature and from several databases such as USDA GRIN global[55] (accessed on December 2022) and the Harlan and De Wet CWR inventory[56] (accessed on December 2022) for all the CWRs that had information available. Additionally, 50 seed and plant traits (Supplementary data) were gathered from TRY database[26]. This database includes specific traits and plant characterisation that have been published or reported in other databases, research articles or unpublished data.

Distribution and conservation data

The distribution and accepted scientific names were downloaded and matched from World Checklist of Vascular Plants[60] (version 9, accessed on February 2022) for all taxa. The distribution of introduced species was not included to focus on the native distribution of wild species. For the geographical distribution we used the Biodiversity Information Standards (before known as Taxonomic Databases Working Group, TDWG) level 3. We used the IUCN Red List[91] (accessed on September 2022) and the ThreatSearch tool from Botanic Gardens Conservation International (BCGI[112], accessed on September 2022) to assess the global threat status of the Brassicaceae species. Similarly, global records of *ex situ* collections were gathered using Genesys[113] (Data accessed through Genesys on November 2022 via R package *genesysr*[114]) and the Millennium Seed Bank Partnership database[115] (accessed on September 2022). The conservation status for the 1,242 species were extracted using *rredlist* package[116]. The analysis of the data for this section was performed in R[100] (v. 4.2.1) unless otherwise specified, using the following R packages: To curate, visualise and analyse the data we used: *cowplot*[117](v.1.1.1), *data.table*[118] (v1.14.8), *geojson*[119] (v.0.3.5), *sf*[120] (v.1.0.14) and *tidyverse* [101](v2.0.0).

Data Availability

The sequence data used in this study and the script for curating the data are available in the GigaScience database GigaDB[121]. Lists of NCBI accession numbers and taxa are also available via GigaDB (file names: “rbcl_id_spp.csv”; “its_id_sp.csv”; “matk_id_spp.csv “; “trn_id_spp.csv“) Additional supporting data including the database with new CWRs, authorships of the taxa and extra information of the species are available via Figshare[122].

Supplementary material Fig. S1. Phylogenetic trees from each marker evaluated, a) matK, b) ITS, c) rbcL and d) trnL-F

Supplementary material Fig. S2. Proportion of number of entries in the NCBI for nine markers in the Brassicaceae family

Supplementary material Table S1. Classification of Brassicaceae wild species as CWRs combining two global databases

Acknowledgments

ECL is supported by the Kew Future Leaders Fellowship from the Royal Botanic Gardens, Kew. The Royal Botanic Gardens, Kew receives grant-in-aid from Defra. The project “Adapting Agriculture to Climate Change: Collecting, Protecting and Preparing Crop Wild Relatives”, which is supported by the Government of Norway (grant number from the Norwegian Government: QZA-14/0005) has partly funded the publication of this article.

Authors contribution

ECL, EB and JV conceived and designed the study. ECL and PGB compiled and processed data. ECL, PGB and JV wrote and ran the code. ECL, EB and JV interpreted the results. ECL wrote the paper. ECL, EB, PGB and JV edited and commented the paper.

Declaration of interests

The authors declare no competing interests.

Table 1

Table 1 List of cultivated Brassicaceae and potential CWR (crop wild relatives) species based on phylogenetic distances (PD) between them for the two genes used in the phylogenetic tree, *mat*K and ITS from Fig. 1. (Y) represents conventional crosses reported for classified CWRs (based on Gene Pool classification listed in Supplementary material Table S1), (Y*) represents conventional crosses with very low success, (N) are unsuccessful conventional crosses (or crosses that needed biotechnology). "NI" means no information was found for their crosses [37, 42, 94 and references within them]. We represented with 'NA' the species that were not present in one or both trees. Authorships and ID for the scientific name of the species are listed in the Supplementary data file. **Bold** taxa represent the new CWRs identified using the PD (to see the complete and detailed list please view Supplementary data[122]).

Crop	CWRs *mat*K	PD	CWRs ITS	PD
Barbarea verna (TG)	***B. orthoceras, B. vulgaris***	0.00428	NA	NA
Barbarea vulgaris (TG)	***B. orthoceras***	0.00369	NA	NA
	B. verna	0.00428		
Brassica carinata	*Brassica nigra* (Y)	0.00187	*Brassica nigra* (Y)	0.02790
	Diplotaxis catholica	0.01131	***B. deflexa,*** *B. maurorum, Coincya tournefortii,* ***B. balearica,*** *B. fruticulosa,* ***B. oxyrrhina****, B. barrelieri****, Diplotaxis spp., Erucastrum spp.,*** *Sinapis spp., Raphanus spp.,* ***Rapistrum rugosum****.*	0.14164
	Kremeriella cordylocarpus, *Sinapis alba*	0.01251	*B. napus (Y), B. juncea (Y), Brassica spp.,* ***Moricandia spp****.*	0.16518
	B. spinescens*,* ***Rapistrum rugosum,*** *Sinapis arvensis*	0.0158-0.01592	*Orychophragmus violaceus (Y)*	0.19612
	Coincya tournefortii, ***Erucastrum spp., Coincya spp., Crambe spp****.*	0.02118-0.02505		
	Orychophragmus violaceus (Y), ***Enarthrocarpus lyratus****, Eruca spp., Raphanus spp., B. napus (Y), B. juncea (Y)*	0.03134		

Brassica juncea	*B. rapa (Y*), B. oleracea (Y), B. napus (Y)*	0.00228-0.00246	*B. rapa (Y*), B. napus (Y)*	0.02807
	Enarthrocarpus spp., *Raphanus spp.*	0.01692	*B. insularis, B. macrocarpa, B. villosa, B. cretica, B. oleracea, B. montana*	0.11898
	Erucastrum spp., Diplotaxis spp., Coincya spp., Eruca spp., B. carinata (Y), B. nigra (Y), Coincya tournefortii, ***Crambe spp***., *Sinapis spp., Orychophragmus violaceus (Y*)**,** ***Sisymbrium spp., Kremeriella cordylocarpu****s*, ***Rapistrum rugosum.***	0.0256-0.03454	*B. carinata (Y), B. nigra (Y),* ***B. deflexa***, *Coincya tournefortii, B. barrelieri, Eruca spp.,* ***Erucastrum spp***., ***Moricandia spp***., *Raphanus spp., Sinapis spp.*	0.16518
			Orychophragmus violaceus (Y)	0.19612
Brassica napus	*B. juncea (Y), B. rapa (Y), B. oleracea (Y)*	0.00246	*B. rapa (Y)*	0.000003
	Enarthrocarpus spp., Erucastrum spp., *Raphanus spp.*	0.01692	*B. juncea (Y)*	0.02807
	Eruca spp., ***Diplotaxis spp***., *B. carinata (Y), Coincya tournefortii, B. nigra,* ***Crambe spp***.	0.02103-0.03134	*B. insularis, B. macrocarpa, B. villosa, B. cretica, B. oleracea (Y), B. montana*	0.11898
			B. deflexa, ***Erucastrum spp***., *B. carinata (Y),* ***Diplotaxis spp***., ***Sisymbrium spp***., *Sinapis spp.,* ***Moricandia spp***.	0.16518
Brassica nigra	*B. carinata (N)*	0.00187	*B. carinata (N)*	0.02790
	Diplotaxis spp., ***Kremeriella cordylocarpus***, *Sinapis alba*	0.01131-0.0125	***B. deflexa***, *B. maurorum (Y*), Sinapis arvensis (Y*), Coincya tournefortii,* ***B. oxyrrhina***, *B. barrelieri,* ***B. balearica***, *B. fruticulosa,* ***Diplotaxis spp., Erucastrum spp., Rapistrum rugosum***, *Raphanus spp.,* ***Rapistrum rugosum***.	0.14146

	**B. spinescens**, Sinapis arvensis (Y*), **Rapistrum rugosum**	0.01578	B. juncea (Y), **Moricandia spp**., Brassica spp.	0.16518
	**Coincya spp., Crambe spp**., B. napus, B. oleracea (N), B. juncea (Y), B. rapa, **Moricandia arvensis**	0.02118-0.03134		
Brassica oleracea	B. rapa (Y)	0.000002	B. montana (Y)	0.00405
	B. juncea (Y), B. napus (Y)	0.00228-0.00246	B. insularis (Y), B. macrocarpa (Y), B. villosa (Y), B. cretica (Y)	0.02898-0.03112
	**Enarthrocarpus spp**., Raphanus spp., **Erucastrum spp**., Eruca spp.	0.01692-0.0260	B. juncea (Y), B. rapa (Y), B. napus (Y)	0.11898
	Coincya tournefortii (Y), B. nigra**, Coincya spp., Erucastrum spp.**, **Crambe spp**.	0.03134	Coincya tournefortii (Y), B. nigra, **Brassica spp**., **Erucastrum spp**., Raphanus sativus	0.16518
Brassica rapa	B. oleracea (Y)	0.000002	B. napus (Y)	0.000003
	B. juncea (Y*), B. napus (Y)	0.00228-0.00246	B. juncea (Y*)	0.02807
	**Enarthrocarpus spp**., Erucastrum gallicum (Y), Raphanus spp.	0.01692	B. oleracea (Y), B. macrocarpa, B. villosa, B. cretica, B. montana, B. insularis	0.11898
	Erucastrum spp., Diplotaxis spp., Coincya spp., C. tournefortii, B. carinata (Y), B. nigra, **Crambe spp., Enarthrocarpus spp**., Eruca spp., Sinapis spp.	0.02103-0.03135	Erucastrum gallicum (Y), B. barrelieri (Y), B. carinata (Y), B. elongata (NI), B. fruticulosa (Y*), **Brassica spp., Diplotaxis spp**., Sinapis spp., Eruca spp., **Moricandia spp., Erucastrum spp**.	0.16518
Diplotaxis tenuifolia	B. oleracea, B. rapa (Y), B. juncea (Y), **Enarthrocarpus spp.,** Erucastrum spp., Eruca spp., **Moricandia arvensis**	0.02599	**B. gravinae**	0.08368

	B. nigra (Y), ***Coincya spp., Crambe spp***.	0.03134	***B. repanda, B. desnottesii****, Eruca spp.,* ***Diplotaxis acris, Moricandia spp***.	0.10650-0.11255
			B. juncea (Y), B. rapa (Y), B. nigra (Y), ***Erucastrum spp., Diplotaxis spp****., Raphanus sativus, Sinapis spp.,* ***Brassica spp***.	0.16518
Eruca vesicaria	*E. sativa*	0.00254	*E. sativa,* ***E. foleyi***	0.06195
	Diplotaxis harra	0.01327	***Diplotaxis acris, Brassica repanda, B. desnottesii***	0.08016-0.09699
	Brassica napus, B. juncea, B. rapa, B. oleracea, ***Enarthrocarpus spp****., Raphanus spp. Diplotaxis tenuifolia (Y),* ***Moricandia arvensis***	0.02103-0.02599	***Brassica gravinae****, Diplotaxis tenuifolia (Y),* ***Moricandia spp****.,* ***Brassica elongata***	0.10650-0.11250
Eutrema japonicum	***E. giganteum, E. tenue*** *(NI)*	0.000002	*E. wasabi*	0.000002
	E. thibeticum, E. bulbiferum, E. yunnanense *(NI)*	0.00349	***E. tenue*** *(NI)*	0.00656
			E. bulbiferum	0.01620
			E. yunnanense *(NI),* ***E. thibeticum, E. giganteum, E. schulzii, E. wuchengyii***	0.03965
Isatis tinctoria (TG)	***I. minima*** *(NI),* ***I. multicaulis***	0.01772	***I. indigotica***	0.003760
			I. pachycarpa, I. takhtajanii, I. glauca, I. kotschyana, I. cappadocica	0.01646
Lepidium meyenii	***L. bonariense*** *(NI),* ***L. squamatum, L. disymum***	0.00107-0.00509	***L. reichei, L. bonariense*** *(NI),* ***L. virginicum***	0.01302
	Lepidium spp.	0.01359	***Lepidium spp.***	0.07325
Lepidium sativum	***L. virginicum, L. densiflorum, L. coronopus***	0.008112	***Lepidium spp.***	0.07560
	Lepidium spp.	0.01359-0.03284		

Nasturtium officinale (NI)	***N. microphyllum***	0.000002	NA	NA
	N. gambelii	0.00505		
Raphanus raphanistrum subsp. sativus	*Raphanus sativus (Y)*	0.00184	*Brassica spp., Sinapis arvensis*	0.07473
	Brassica napus (Y),* ***Enarthrocarpus spp., Erucastrum spp.,*** *Brassica spp.*	0.01692	*Brassica spp.,* ***Diplotaxis spp., Erucastrum spp****., B. napus (Y*)*	0.14164-0.16518
Rorippa indica (TG)	***R. dubia***	0.000002	***R. islandica, R. palustris***	0.01775
	R. cantoniensis, R. islandica	0.00391-0.00406	***R. cantoniensis***	0.02508
	R. palustris*, R. amphibia,* ***R. sylvestris***	0.00507	***R. divaricata***	0.04479
Sinapis alba	*Kremeriella cordylocarpus*	0.00956	***Coincya richeri***	0.08614
	Brassica carinata, B. nigra (Y), B. spinescens,* ***Diplotaxis catholica****, Sinapis arvensis*	0.01251-0.01578	*B. nigra (Y*), Diplotaxis spp.,* ***Erucastrum spp****.,* ***Moricandia spp.****, Eruca spp., Brassica spp.*	0.16518
	Rapistrum rugosum*,* ***Erucastrum spp****., Coincya monensis (Y),* ***Coincya spp****.,* ***Crambe spp.***	0.02119-0.0251		
Sisymbrium officinale (NI)	***S. loeselii, S. orientale, S. luteum, S. altissimum***	0.00361	***S. volgense, S. orientale***	0.03702
			Sisymbrium spp.	0.09579

References

1. FAO, IFAD, UNICEF, WFP and WHO. The State of Food Security and Nutrition in the World 2021: Transforming food systems for food security, improved nutrition and affordable healthy diets for all. FAO, Rome. https://www.fao.org/documents/card/en/c/cb4474en; 2021.
2. FAO. The State of the World's Biodiversity for Food and Agriculture. In: Bélanger J and Pilling D, (eds.). Rome, 572 pp. http://www.fao.org/3/CA3129EN/CA3129EN.pdf: FAO Commission on Genetic Resources for Food and Agriculture Assessments, 2019.
3. Dempewolf H, Eastwood RJ, Guarino L, Khoury CK, Müller JV and Toll J. Adapting agriculture to climate change: a global initiative to collect, conserve and use crop wild relatives. Agroecology and Sustainable Food Systems. 2014;38:369-77.
4. Eastwood RJ, Tambam BB, Aboagye LM, Akparov ZI, Aladele SE, Allen R, et al. Adapting agriculture to climate change: A synopsis of coordinated National Crop Wild Relative Seed Collecting Programs across five continents. Plants. 2022;11 14:1840.
5. Kilian B, Dempewolf H, Guarino L, Werner P, Coyne C and Warburton ML. Crop Science special issue: Adapting agriculture to climate change: A walk on the wild side. Crop Sci. 2021;61:32-6.
6. Castañeda-Álvarez NP, Khoury CK, Achicanoy HA, Bernau V, Dempewolf H, Eastwood RJ, et al. Global conservation priorities for crop wild relatives. Nature Plants. 2016;2(4):16022. doi:10.1038/nplants.2016.22.
7. Kiefer M, Schmickl R, German DA, Mandáková T, Lysak MA, Al-Shehbaz IA, et al. BrassiBase: introduction to a novel knowledge database on Brassicaceae evolution. Plant and Cell Physiology. 2014;55 1:e3. doi: https://brassibase.cos.uni-heidelberg.de.
8. Govaerts R, Nic Lughadha E, Black N, Turner R and Paton A. The World Checklist of Vascular Plants, a continuously updated resource for exploring global plant diversity. Scientific Data. 2021;8 1:215.
9. Tsunoda S, Hinata K and Gómez-Campo C. *Brassica* crops and wild allies. Biology and breeding. Japan Scientific Societies Press, Tokyo 1980.
10. Edger PP, Tang M, Bird KA, Mayfield DR, Conant G, Mummenhoff K, et al. Secondary structure analyses of the nuclear rRNA internal transcribed spacers and assessment of its phylogenetic utility across the Brassicaceae (mustards). PloS one. 2014;9 7:e101341.
11. Hendriks KP, Kiefer C, Al-Shehbaz IA, Bailey CD, Hooft van Huysduynen A, Nikolov LA, et al. Global Brassicaceae phylogeny based on filtering of 1,000-gene dataset. Curr Biol. 2023; doi:10.1016/j.cub.2023.08.026.
12. Liu LM, Du XY, Guo C and Li DZ. Resolving robust phylogenetic relationships of core Brassicaceae using genome skimming data. Journal of Systematics and Evolution. 2021;59 3:442-53.
13. Nikolov LA, Shushkov P, Nevado B, Gan X, Al-Shehbaz IA, Filatov D, et al. Resolving the backbone of the Brassicaceae phylogeny for investigating trait diversity. New Phytologist. 2019;222 3:1638-51.
14. Warwick SI, Mummenhoff K, Sauder CA, Koch MA and Al-Shehbaz IA. Closing the gaps: phylogenetic relationships in the Brassicaceae based on DNA sequence data of nuclear ribosomal ITS region. Plant Systematics and Evolution. 2010;285 3:209-32.
15. Al-Shehbaz I, Beilstein M and Kellogg E. Systematics and phylogeny of the Brassicaceae (Cruciferae): an overview. Plant systematics and evolution. 2006;259:89-120.
16. Abrahams RS. *The Power of Synteny: Deep Evolutionary Insights from Comparative Genomics*. University of Missouri-Columbia, 2021.
17. Arias T and Pires CJ. A fully resolved chloroplast phylogeny of the brassica crops and wild relatives (Brassicaceae: *Brassiceae*): novel clades and potential taxonomics implications. Taxon. 2012;61(5):980-8.
18. Nagaharu U and Nagaharu N. Genome analysis in *Brassica* with special reference to the experimental formation of *B. napus* and peculiar mode of fertilization. Jpn J Bot. 1935;7 7:389-452.

19. Miller RE and Khoury CK. The gene pool concept applied to crop wild relatives: An evolutionary perspective. North American crop wild relatives, volume 1: conservation strategies. 2018:167-88.
20. Vincent H, Wiersema J, Kell S, Fielder H, Dobbie S, Castañeda-Álvarez NP, et al. A prioritized crop wild relative inventory to help underpin global food security. Biological conservation. 2013;167:265-75.
21. Dempewolf H, Baute G, Anderson J, Kilian B, Smith C and Guarino L. Past and future use of wild relatives in crop breeding. Crop science. 2017;57 3:1070-82.
22. Harlan JR and de Wet JM. Toward a rational classification of cultivated plants. Taxon. 1971;20 4:509-17.
23. Maxted N, Ford-Lloyd BV, Jury S, Kell S and Scholten M. Towards a definition of a crop wild relative. Biodiversity & Conservation. 2006;15(8) 8:2673-85.
24. Viruel J, Kantar MB, Gargiulo R, Hesketh-Prichard P, Leong N, Cockel C, et al. Crop wild phylorelatives (CWPs): phylogenetic distance, cytogenetic compatibility and breeding system data enable estimation of crop wild relative gene pool classification. Botanical Journal of the Linnean Society. 2021;195 1:1-33.
25. Warwick SI. Brassicaceae in agriculture. Genetics and Genomics of the Brassicaceae. 2011:33-65.
26. Kattge J, Bönisch G, Díaz S, Lavorel S, Prentice IC, Leadley P, et al. TRY plant trait database–enhanced coverage and open access. Global change biology. 2020;26 1:119-88. https://www.try-db.org/TryWeb/dp.php
27. Ahuja I, Rohloff J and Bones AM. Defence mechanisms of Brassicaceae: implications for plant-insect interactions and potential for integrated pest management. A review. Agronomy for Sustainable Development. 2011;30 2:623-70.
28. Koch S, Dunker S, Kleinhenz B, Röhrig M and Tiedemann Av. A crop loss-related forecasting model for *Sclerotinia* stem rot in winter oilseed rape. Phytopathology. 2007;97 9:1186-94.
29. Singh D, Dhar S and Yadava D. Genetic and pathogenic variability of Indian strains of *Xanthomonas campestris* pv. *campestris* causing black rot disease in crucifers. Current microbiology. 2011;63:551-60.
30. Branca F and Cartea E. Brassica. In: Kole C, editor. Wild crop relatives: genomic and breeding resources. Springer; 2011. p. 17-36.
31. Quezada-Martinez D, Addo Nyarko CP, Schiessl SV and Mason AS. Using wild relatives and related species to build climate resilience in *Brassica* crops. Theoretical and Applied Genetics. 2021;134 6:1711-28.
32. Warwick SI and Hall JC. Phylogeny of *Brassica* and wild relatives. In: Gupta SK, editor. Biology and breeding of crucifers. CRC Press, Boca Raton; 2009. p. 19-36.
33. Fahey JW, Zalcmann AT and Talalay P. The chemical diversity and distribution of glucosinolates and isothiocyanates among plants. Phytochemistry. 2001;56 1:5-51.
34. Kumar M, Choi J-Y, Kumari N, Pareek A and Kim S-R. Molecular breeding in *Brassica* for salt tolerance: importance of microsatellite (SSR) markers for molecular breeding in *Brassica*. Frontiers in plant science. 2015;6:688.
35. Ozturk E, Ozer H and Polat T. Growth and yield of safflower genotypes grown under irrigated and non-irrigated conditions in a highland environment. Plant Soil and Environment. 2008;54 10:453-60.
36. Chandra A, Gupta M, Banga S and Banga S. Production of an interspecific hybrid between *Brassica fruticulosa* and *B. rapa*. Plant breeding. 2004;123 5:497-8.
37. Kumar A, Singh BK, Singh VV and Chauhan JS. Cytomorphological and molecular evidences of synthesis of interspecific hybrids between *Brassica rapa* and *B. fruticulosa* through sexual hybridization. Australian Journal of Crop Science. 2013;7 6:849-54.
38. Chen H-F, Wang H and Li Z-Y. Production and genetic analysis of partial hybrids in intertribal crosses between *Brassica* species (*B. rapa, B. napus*) and *Capsella bursa-pastoris*. Plant cell reports. 2007;26:1791-800.

39. Katche E, Quezada-Martinez D, Katche EI, Vasquez-Teuber P and Mason AS. Interspecific hybridization for *Brassica* crop improvement. Crop Breeding, Genetics and Genomics. 2019;1 1.
40. Rana K, Atri C, Gupta M, Akhatar J, Sandhu PS, Kumar N, et al. Mapping resistance responses to *Sclerotinia* infestation in introgression lines of *Brassica juncea* carrying genomic segments from wild Brassicaceae *B. fruticulosa*. Scientific Reports. 2017;7 1:1-12.
41. Traka MH, Saha S, Huseby S, Kopriva S, Walley PG, Barker GC, et al. Genetic regulation of glucoraphanin accumulation in Beneforté® broccoli. New Phytologist. 2013;198 4:1085-95.
42. FitzJohn RG, Armstrong TT, Newstrom-Lloyd LE, Wilton AD and Cochrane M. Hybridisation within *Brassica* and allied genera: evaluation of potential for transgene escape. Euphytica. 2007;158:209-30.
43. Warwick S, Francis A and Gugel R. Guide to wild germplasm of *Brassica* and allied crops (tribe *Brassiceae,* Brassicaceae). Canada: Agriculture and Agri-Food Canada. 2009;1 6.
44. Hunter D. *Crop wild relatives: a manual of in situ conservation.* Routledge. 2012
45. Kell S, Maxted N and Bilz M. European crop wild relative threat assessment: knowledge gained and lessons learnt. In: Maxted N, Dulloo ME, Ford-Lloyd BV, Frese L, Iriondo J and de Carvalho MAP, editors. Agrobiodiversity conservation: securing the diversity of crop wild relatives and landraces. CABI Wallingford UK; 2012. p. 218-42.
46. Rahman W, Brehm JM, Maxted N, Phillips J, Contreras-Toledo AR, Faraji M, et al. Gap analyses of priority wild relatives of food crop in current *ex situ* and *in situ* conservation in Indonesia. Biodiversity and Conservation. 2021;30:2827-55.
47. Khoury CK, Greene S, Wiersema J, Maxted N, Jarvis A and Struik PC. An inventory of crop wild relatives of the United States. Crop Science. 2013;53 4:1496-508.
48. Zair W, Maxted N, Brehm JM and Amri A. *Ex situ* and *in situ* conservation gap analysis of crop wild relative diversity in the Fertile Crescent of the Middle East. Genetic Resources and Crop Evolution. 2021;68:693-709.
49. Rubio Teso ML, Álvarez Muñiz C, Gaisberger H, Kell S, Lara-Romero C, Magos Brehm J, et al. In situ plant genetic resources in Europe: crop wild relatives. Farmer's Pride. 2020:134.
50. Vincent H, Von Bothmer R, Knüpffer H, Amri A, Konopka J and Maxted N. Genetic gap analysis of wild *Hordeum* taxa. Plant Genetic Resources. 2012;10 3:242-53.
51. Khoury CK, Carver D, Barchenger DW, Barboza GE, van Zonneveld M, Jarret R, et al. Modelled distributions and conservation status of the wild relatives of chile peppers (*Capsicum* L.). Diversity and Distributions. 2020;26 2:209-25.
52. Castañeda-Álvarez NP, De Haan S, Juárez H, Khoury CK, Achicanoy HA, Sosa CC, et al. *Ex situ* conservation priorities for the wild relatives of potato (*Solanum* L. section Petota). PLoS One. 2015;10 4:e0122599.
53. Ramirez-Villegas J, Khoury CK, Achicanoy HA, Diaz MV, Mendez AC, Sosa CC, et al. State of *ex situ* conservation of landrace groups of 25 major crops. Nature Plants. 2022;8 5:491-9.
54. Maxted N, Kell S, Ford-Lloyd B, Dulloo E and Toledo Á. Toward the systematic conservation of global crop wild relative diversity. Crop Science. 2012;52 2:774-85.
55. USDA, Service AR and System NPG: Germplasm Resources Information Network (GRIN Taxonomy). (2022). https://npgsweb.ars-grin.gov/gringlobal/taxon/taxonomysearchcwr Accessed December 2022.
56. CWR Inventory: Crop wild relatives checklist. (2010). https://www.cwrdiversity.org/ Accessed December 2022.
57. Müller KF, Borsch T and Hilu KW. Phylogenetic utility of rapidly evolving DNA at high taxonomical levels: contrasting matK, *trn*T-F, and *rbc*L in basal angiosperms. Molecular phylogenetics and evolution. 2006;41 1:99-117.
58. CBOL Plant Working Group 1, Hollingsworth PM, Forrest LL, Spouge JL, Hajibabaei M, Ratnasingham S, et al. A DNA barcode for land plants. Proceedings of the National Academy of Sciences. 2009;106 31:12794-7.
59. Hao G, Al-Shehbaz IA, Ahani H, Liang Q, Mao K, Wang Q, et al. An integrative study of evolutionary diversification of *Eutrema* (*Eutremeae*, Brassicaceae). Botanical Journal of the Linnean Society. 2017;184 2:204-23.

60. Govaerts, R. World Checklist of Vascular Plants (WCVP) Version 12. Board of Trustees of the Royal Botanic Gardens, Kew, Kew, UK. 2023. https://powo.science.kew.org/ Accessed February 2022.
61. Lysak MA and Koch MA. Phylogeny, genome, and karyotype evolution of crucifers (Brassicaceae). Genetics and Genomics of the Brassicaceae. 2011. p. 1-31.
62. Marhold K and Lihová J. Polyploidy, hybridization and reticulate evolution: lessons from the Brassicaceae. Plant systematics and evolution. 2006;259:143-74.
63. Kunakh V, Adonin V, Ozheredov S and Blyum YB. Mixoploidy in wild and cultivated species of Cruciferae capable of hybridizing with rapeseed *Brassica napus*. Cytology and Genetics. 2008;42:204-9.
64. Lysak MA, Koch MA, Pecinka A and Schubert I. Chromosome triplication found across the tribe *Brassiceae*. Genome research. 2005;15 4:516-25.
65. Choudhary B and Joshi P. Genetic diversity in advanced derivatives of *Brassica* interspecific hybrids. Euphytica. 2001;121:1-7.
66. Choudhary B, Joshi P and Rao SR. Cytogenetics of *Brassica juncea*× *Brassica rapa* hybrids and patterns of variation in the hybrid derivatives. Plant Breeding. 2002;121 4:292-6.
67. von Bothmer R, Gustafsson M and Snogerup S. *Brassica* sect. *Brassica* (Brassicaceae) II. Inter- and intraspecific crosses with cultivars of *B. oleracea*. Genetic Resources and Crop Evolution. 1995;42:165-78.
68. Li Z and Heneen W. Production and cytogenetics of intergeneric hybrids between the three cultivated *Brassica* diploids and *Orychophragmus violaceus*. Theoretical and applied genetics. 1999;99:694-704.
69. Li Z, Wu J, Liu Y, Liu H and Heneen W. Production and cytogenetics of the intergeneric hybrids *Brassica juncea* × *Orychophragmus violaceus* and *B. carinata* × *O. violaceus*. Theoretical and Applied Genetics. 1998;96:251-65.
70. Lefol E, Séguin-Swartz G and Downey RK. Sexual hybridisation in crosses of cultivated *Brassica* species with the crucifers *Erucastrum gallicum* and *Raphanus raphanistrum*: potential for gene introgression. Euphytica. 1997;95:127-39.
71. Salisbury PA. *Genetic variability in Australian wild crucifers and its potential utilisation in oilseed Brassica species*. La Trobe University, 1991.
72. Matsuzawa Y, Funayama T, Kamibayashi M, Konnai M, Bang S and Kaneko Y. Synthetic *Brassica rapa-Raphanus sativus* amphidiploid lines developed by reciprocal hybridization. Plant breeding. 2000;119 4:357-9.
73. Qiong H, Yunchang L and Desheng M. Introgression of genes from wild crucifers. In: Gupta SK, editor. Biology and breeding of crucifers CRC Press, Boca Raton; 2009. p. 261-83.
74. Rieger M, Potter T, Preston C and Powles S. Hybridisation between *Brassica napus* L. and *Raphanus raphanistrum* L. under agronomic field conditions. Theoretical and Applied Genetics. 2001;103:555-60.
75. Walters SA, Bernhardt P, Joseph M and Miller AJ. Pollination and sterility in horseradish. Plant Breeding. 2016;135 6:735-42.
76. Pignone D and Martínez-Laborde JB. Diplotaxis. In: Kole C, editor. Wild Crop Relatives: Genomic and Breeding Resources: Oilseeds. Springer; 2010. p. 137-47.
77. Byrne SL, Erthmann PØ, Agerbirk N, Bak S, Hauser TP, Nagy I, et al. The genome sequence of *Barbarea vulgaris* facilitates the study of ecological biochemistry. Scientific reports. 2017;7 1:1-14.
78. Badenes-Pérez FR and López-Pérez JA. Resistance and susceptibility to powdery mildew, root-knot nematode, and western flower thrips in two types of winter cress (Brassicaceae). Crop protection. 2018;110:41-7.
79. Kang M, Wu H, Yang Q, Huang L, Hu Q, Ma T, et al. A chromosome-scale genome assembly of *Isatis indigotica*, an important medicinal plant used in traditional Chinese medicine: An *Isatis* genome. Horticulture research. 2020;7.
80. Klimek-Szczykutowicz M, Szopa A and Ekiert H. Chemical composition, traditional and professional use in medicine, application in environmental protection, position in food and cosmetics industries, and biotechnological studies of *Nasturtium officinale* (watercress): a review. Fitoterapia. 2018;129:283-92.

81. Zeb A. Phenolic profile and antioxidant potential of wild watercress (*Nasturtium officinale* L.). SpringerPlus. 2015;4 1:1-7.
82. Zorzan M, Zucca P, Collazuol D, Peddio S, Rescigno A and Pezzani R. *Sisymbrium officinale*, the plant of singers: A review of its properties and uses. Planta Medica. 2020;86 05:307-11.
83. Spataro G and Negri V. Adaptability and variation in *Isatis tinctoria* L.: a new crop for Europe. Euphytica. 2008;163:89-102.
84. Engelen-Eigles G, Holden G, Cohen JD and Gardner G. The effect of temperature, photoperiod, and light quality on gluconasturtiin concentration in watercress (*Nasturtium officinale* R. Br.). Journal of agricultural and food chemistry. 2006;54 2:328-34.
85. Han T-S, Hu Z-Y, Du Z-Q, Zheng Q-J, Liu J, Mitchell-Olds T, et al. Adaptive responses drive the success of polyploid yellowcresses (*Rorippa*, Brassicaceae) in the Hengduan Mountains, a temperate biodiversity hotspot. Plant Diversity. 2022;44 5:455-67.
86. Zhang X, Lu G, Long W, Zou X, Li F and Nishio T. Recent progress in drought and salt tolerance studies in *Brassica* crops. Breeding science. 2014;64 1:60-73.
87. Castillo-Lorenzo E, Finch-Savage W, Seal C and Pritchard H. Adaptive significance of functional germination traits in crop wild relatives of Brassica. Agricultural and forest meteorology. 2019;264:343-50.
88. Essoh AP, Monteiro F, Pena AR, Pais MS, Moura M and Romeiras MM. Exploring glucosinolates diversity in Brassicaceae: a genomic and chemical assessment for deciphering abiotic stress tolerance. Plant Physiology and Biochemistry. 2020;150:151-61.
89. Pratap A and Gupta S. Biology and ecology of wild crucifers. In: Gupta SK, editor. Biology and breeding of crucifers. CRC Press, Boca Raton; 2009. p. 37-67.
90. Razmjoo K, Toriyama K, Ishii R and Hinata K. Photosynthetic properties of hybrids between *Diplotaxis muralis* DC, a C3 species, and *Moricandia arvensis* (L.) DC, a C3-C4 intermediate species in Brassicaceae. Genes & Genetic Systems. 1996;71 3:189-92.
91. IUCN: The International Union for Conservation of Nature Red List of Threatened Species. (2020). https://www.iucnredlist.org/. Accessed September 2022.
92. Vavilov N. Centers of Origin of Cultivated Plants. Inst Appl Bot Plant breed. 1926;16 2.
93. Mabry ME, Turner-Hissong SD, Gallagher EY, McAlvay AC, An H, Edger PP, et al. The evolutionary history of wild, domesticated, and feral *Brassica oleracea* (Brassicaceae). Molecular biology and evolution. 2021;38 10:4419-34.
94. McAlvay AC, Ragsdale AP, Mabry ME, Qi X, Bird KA, Velasco P, et al. *Brassica rapa* domestication: untangling wild and feral forms and convergence of crop morphotypes. Molecular biology and evolution. 2021;38 8:3358-72.
95. FAO. International treaty on plant genetic resources for food and agriculture. Rome 2009.
96. Jombart T and Dray S. Adephylo: exploratory analyses for the phylogenetic comparative method. Version 1.1.13. Bioinformatics. 2010;26 15:1-21.
97. NCBI, National Center for Biotechnology Information (1988). https://www.ncbi.nlm.nih.gov/ Accessed November 2022.
98. Pellicer J and Leitch IJ. The Plant DNA C-values database (release 7.1): an updated online repository of plant genome size data for comparative studies. New Phytologist. 2019;226:301–5. https://cvalues.science.kew.org/.
99. Rice A, Glick L, Abadi S, Einhorn M, Kopelman NM, Salman-Minkov A, et al. The Chromosome Counts Database (CCDB)–a community resource of plant chromosome numbers. New Phytologist. 2015;206 1:19-26, https://taux.evolseq.net/CCDB_web/home/
100. R Core Team. R: A language and environment for statistical computing. Vienna, Austria: R Foundation for Statistical Computing; 2023.
101. Wickham H, Averick M, Bryan J, Chang W, McGowan LDA, François R, et al. Welcome to the Tidyverse. Journal of open source software. 2019;4 43:1686.
102. Charif D and Lobry JR. SeqinR 1.0-2: a contributed package to the R project for statistical computing devoted to biological sequences retrieval and analysis. Structural approaches to sequence evolution: Molecules, networks, populations. Springer; 2007. p. 207-32.
103. Paradis E and Schliep K. ape 5.0: an environment for modern phylogenetics and evolutionary analyses in R. Bioinformatics. 2019;35 3:526-8.

104. Katoh K and Standley DM. MAFFT multiple sequence alignment software version 7: improvements in performance and usability. Molecular biology and evolution. 2013;30 4:772-80.
105. Capella-Gutiérrez S, Silla-Martínez JM and Gabaldón T. trimAl: a tool for automated alignment trimming in large-scale phylogenetic analyses. Bioinformatics. 2009;25 15:1972-3.
106. Borowiec ML. AMAS: a fast tool for alignment manipulation and computing of summary statistics. PeerJ. 2016;4:e1660.
107. Minh BQ, Schmidt HA, Chernomor O, Schrempf D, Woodhams MD, Von Haeseler A, et al. IQ-TREE 2: new models and efficient methods for phylogenetic inference in the genomic era. Molecular biology and evolution. 2020;37 5:1530-4.
108. Schliep KP. phangorn: phylogenetic analysis in R (version 2.11.1). Bioinformatics. 2011;27 4:592-3.
109. Revell LJ. phytools: an R package for phylogenetic comparative biology (and other things). Methods in ecology and evolution. 2012; 2:217-23.
110. Villanueva RAM and Chen ZJ. ggplot2: elegant graphics for data analysis. Taylor & Francis, 2019.
111. Ooms J. Magick: Advanced Graphics and Image-Processing in R. R package version 2.8.0. https://CRAN.R-project.org/package=magick2023.
112. BGCI: ThreatSearch. Botanic Gardens Conservation International https://members.bgci.org/data_tools/threatsearch Accessed September 2022.
113. Genesys-pgr: Genesys plant genetic resources (2014), https://www.genesys-pgr.org/ Accessed November 2022.
114. Obreza M. genesysr: Genesys PGR Client. . R package version 200. 2023.
115. MSBP Data Warehouse: Millennium Seed Bank Partnership (MSBP) portal for seed collections data. https://brahmsonline.kew.org/msbp/SeedData/DW Accessed September 2022.
116. Gearty W and Chamberlain S. rredlist:'IUCN'red list client. R package version 071. 2022.
117. Wilke C. Streamlined Plot Theme and Plot Annotations for "ggplot2" [R Package Cowplot Version 1.1. 1]. https://CRAN.R-project.org/package=cowplot2020.
118. Dowle M and Srinivasan A. data. table: Extension of 'data. frame'. R package version 1148. 2023;1 8.
119. Chamberlain S and Ooms J. geojson: Classes for'GeoJSON' R package version 035. 2023.
120. Pebesma E and Bivand R. Spatial data science: With applications in R. CRC Press; 2023.
121. Castillo-Lorenzo E, Breman E, Gómez Barreiro P, Viruel J. Supporting data for "Current status of global conservation and characterisation of wild and cultivated Brassicaceae genetic resources". GigaScience Database. 2024. https://doi.org/10.5524/102536
122. Castillo-Lorenzo E, Breman E, Gómez Barreiro P, Viruel J. Supplementary_data_Castillo-Lorenzo et al., 2024_Current status of global conservation and characterisation of wild and cultivated Brassicaceae genetic resources. Figshare. 2024. https://doi.org/10.6084/m9.figshare.25002656.v8

Figure1 Click here to access/download;Figure;Figure1.pdf

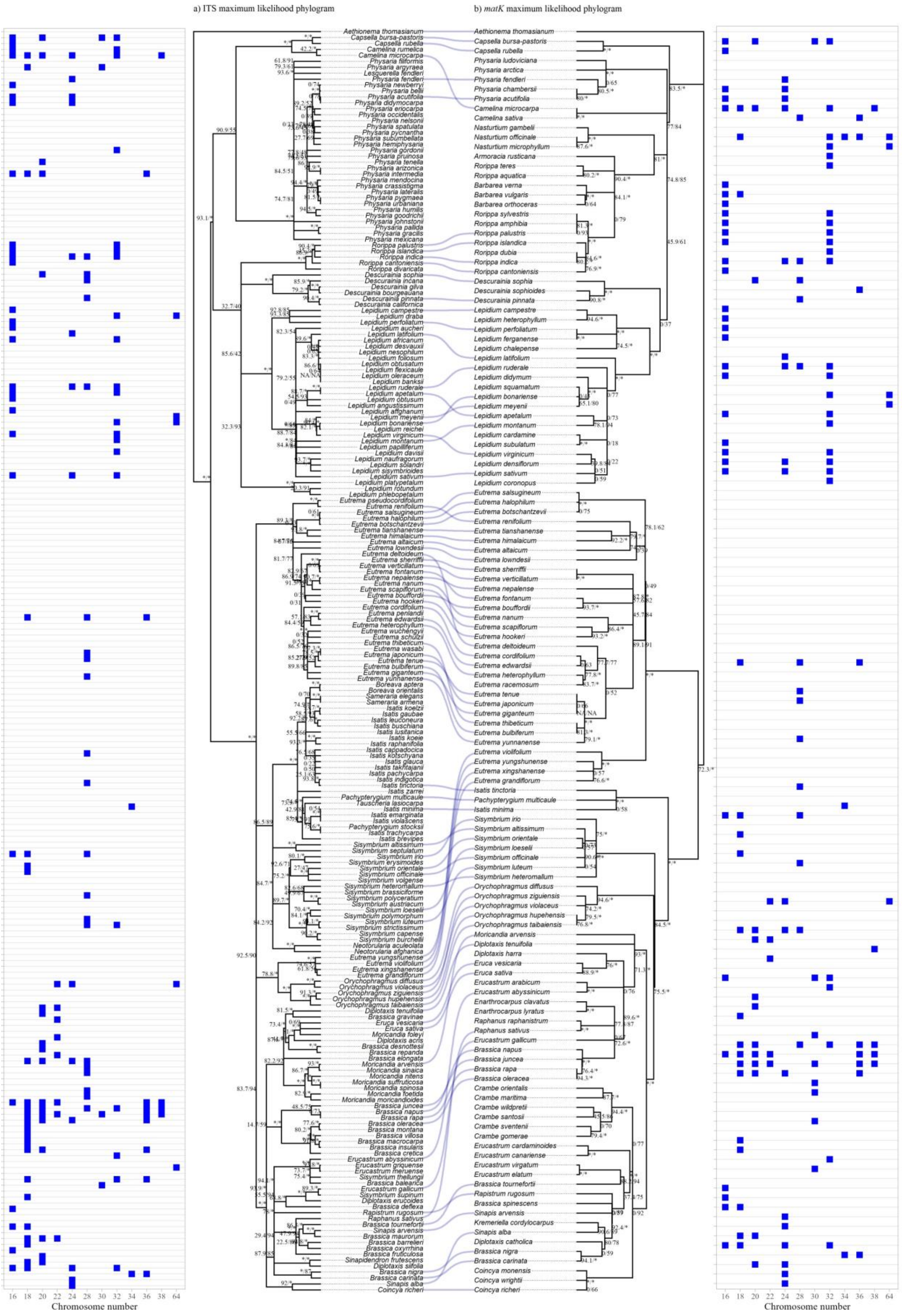

Figure 1. A comparison of two phylogenetic trees, nuclear marker ITS (a) and plastid marker *mat*K (b), reconstructed using maximum likelihood approaches for the data available in the GenBank for Brassicaceae. When the bootstrap is greater or

equal to 95, it is represented with an asterisk *. Chromosome numbers reported in the literature are shown for each species. See Supplementary data for more details.

Figure1

Click here to access/download;Figure;Fig_1.tiff

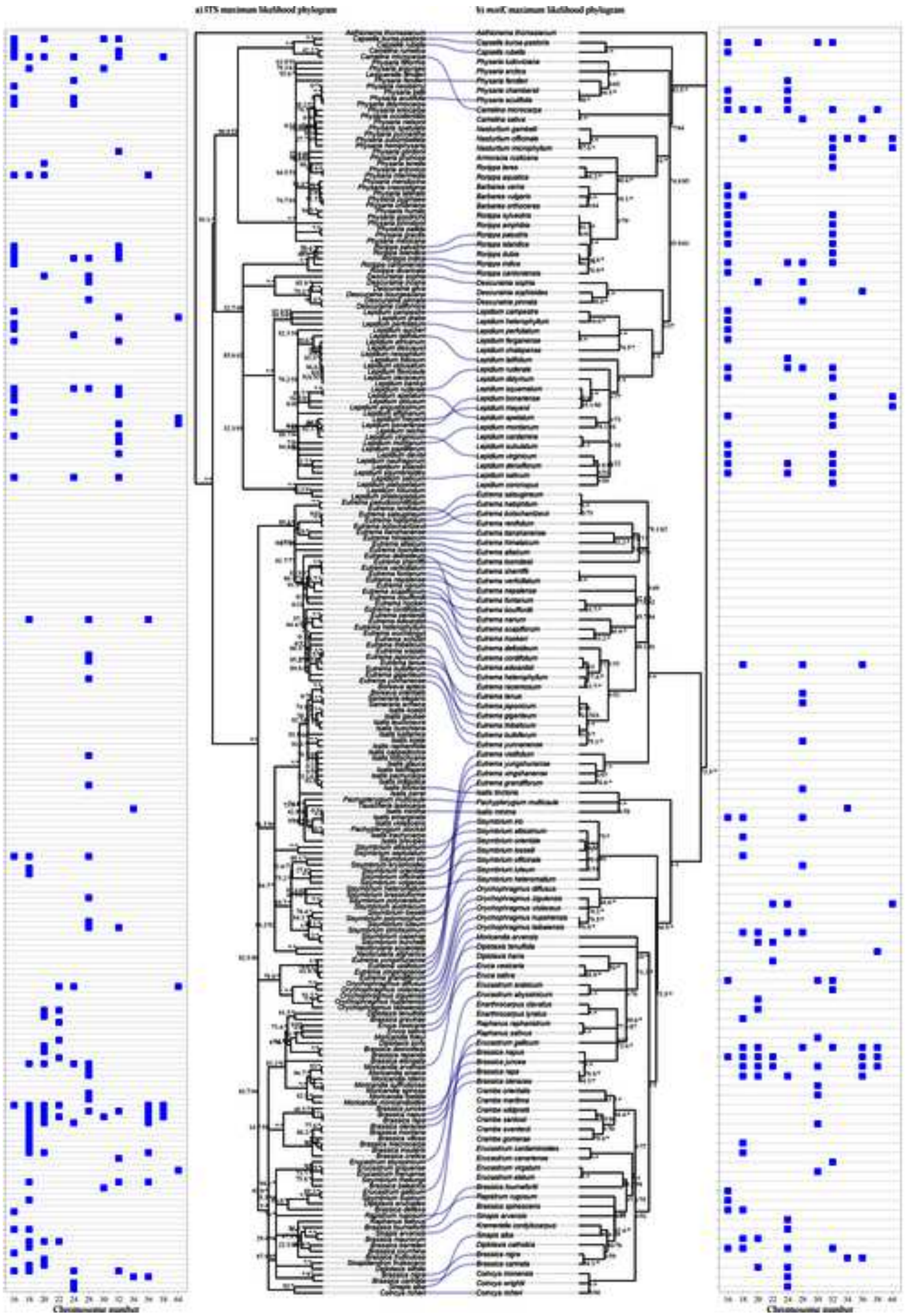

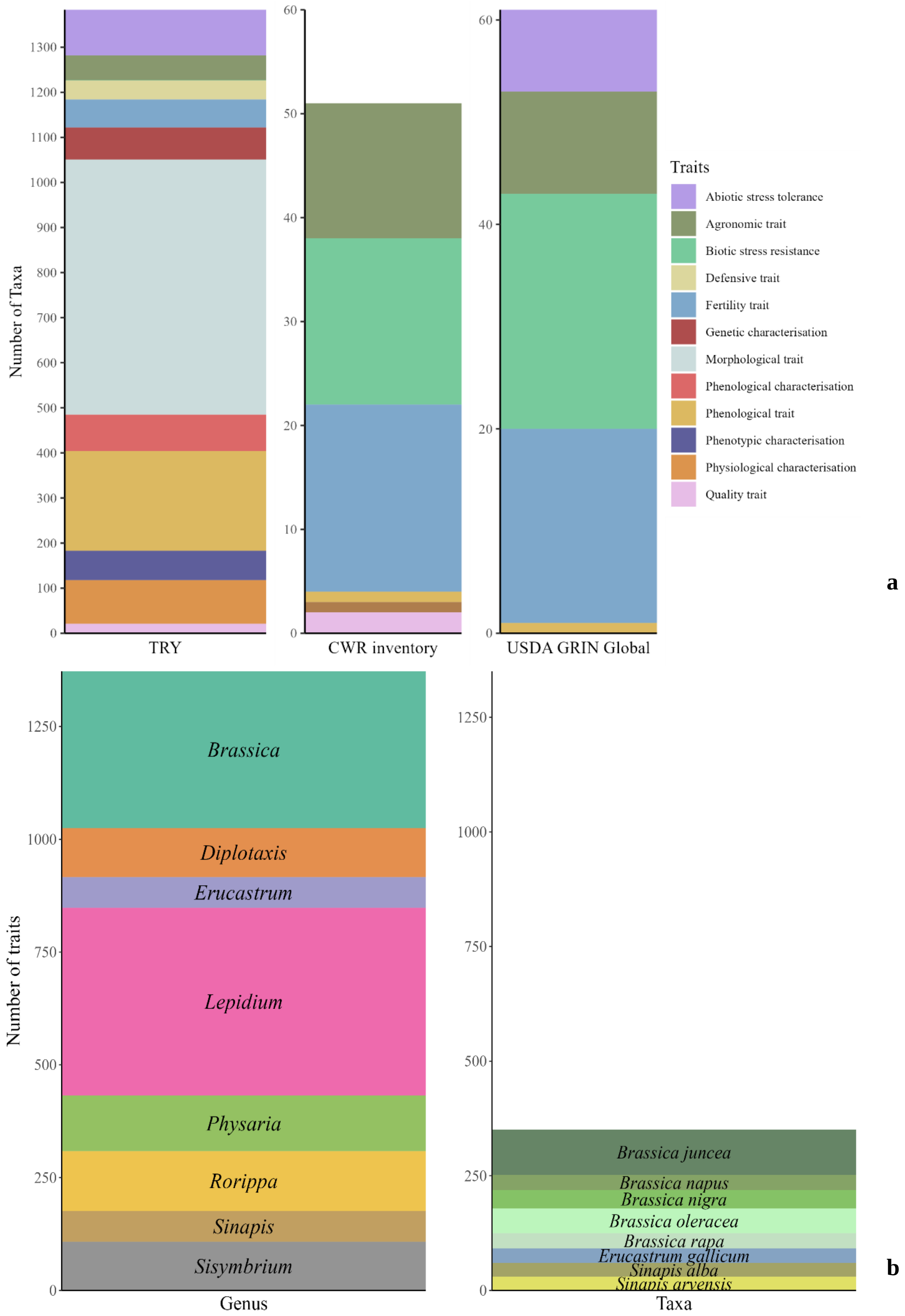

Figure 2. Top traits from global databases for all Brassicaceae available (a) and the top eight genera and species with the most traits characterised (b). This information was obtained from a total of 599 taxa.

Figure2a

Click here to access/download;Figure;Fig.2a.tiff

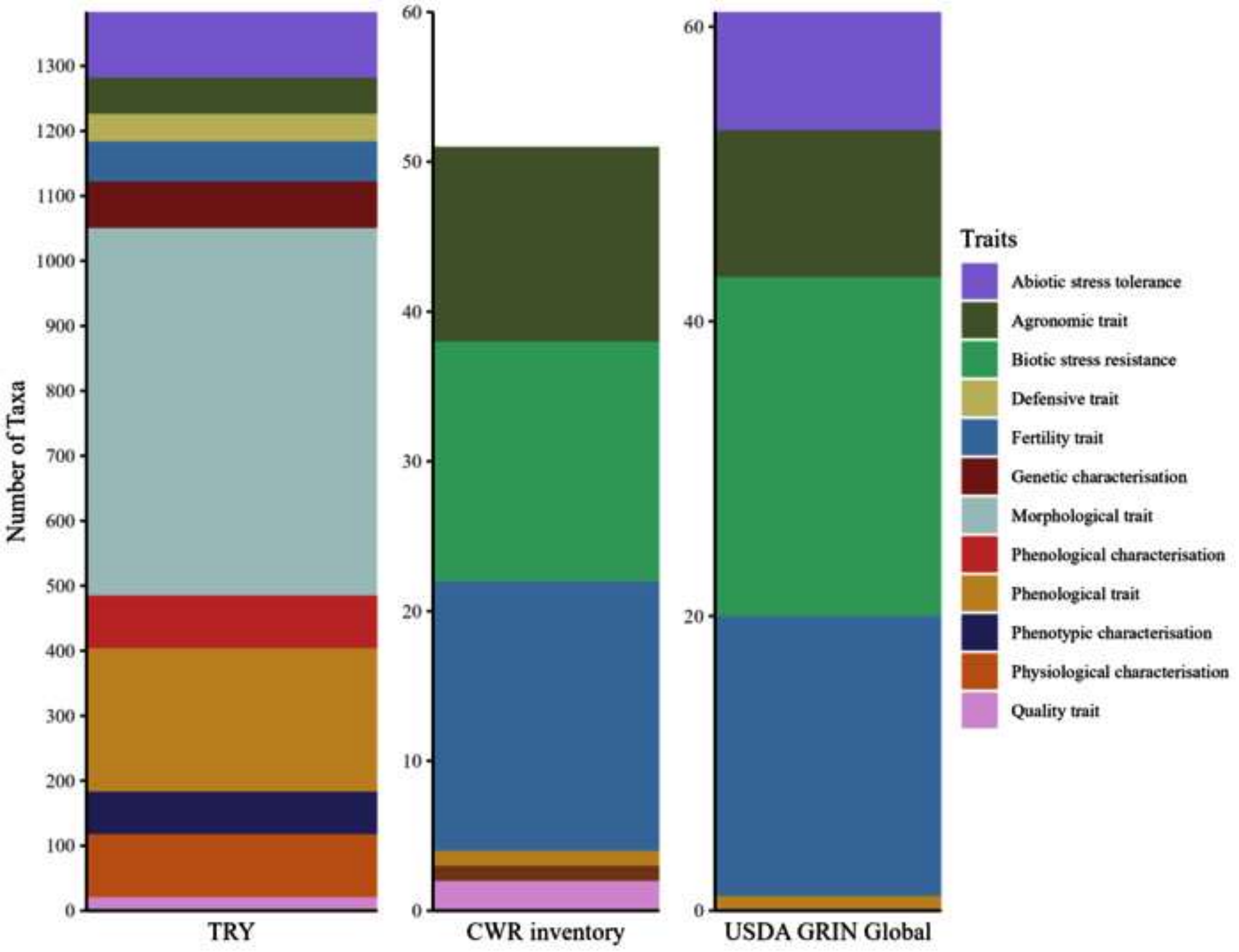

Figure2b

Click here to access/download;Figure;Fig.2b.tiff

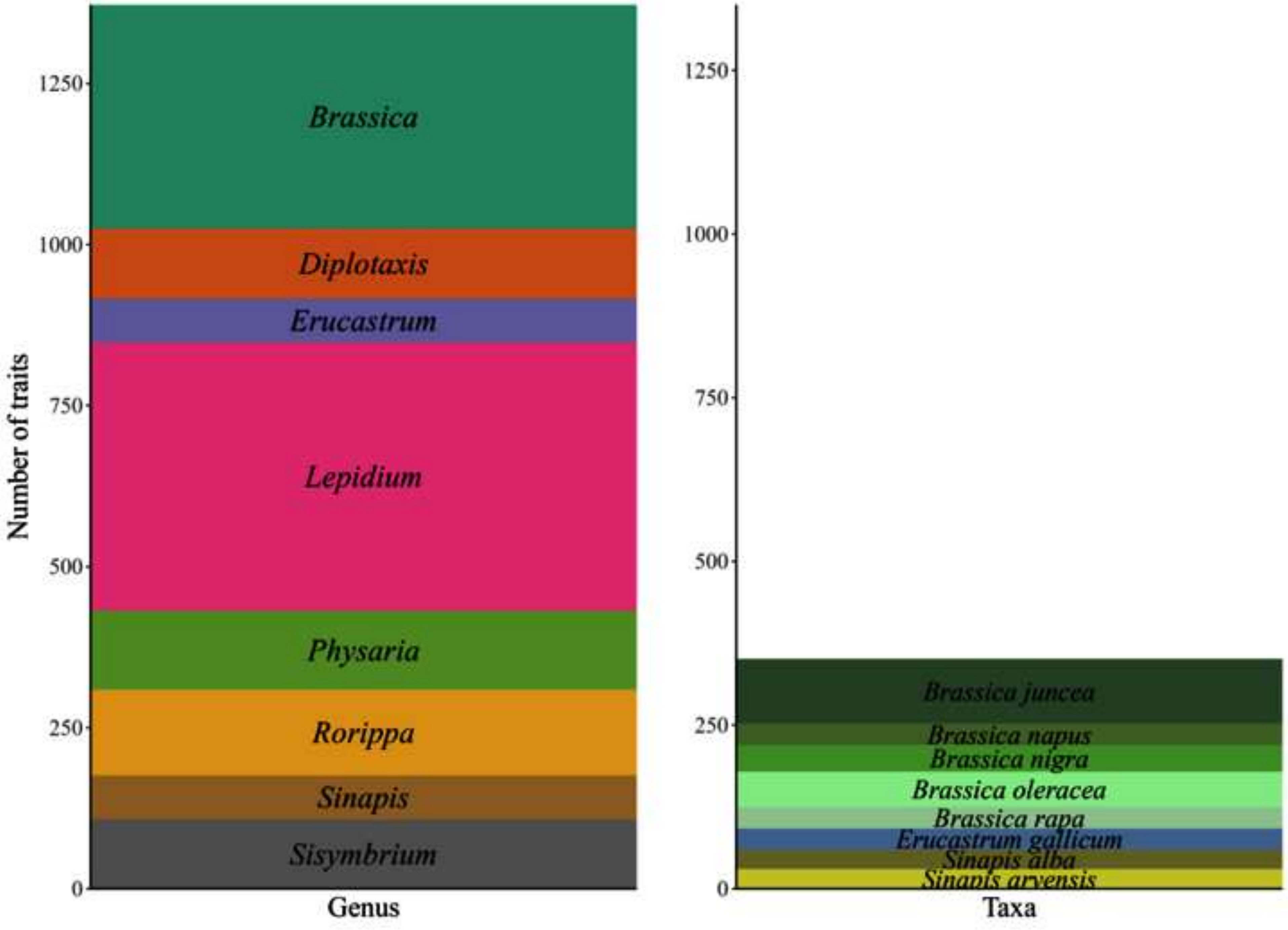

Figure3 Click here to access/download;Figure;Figure3.pdf

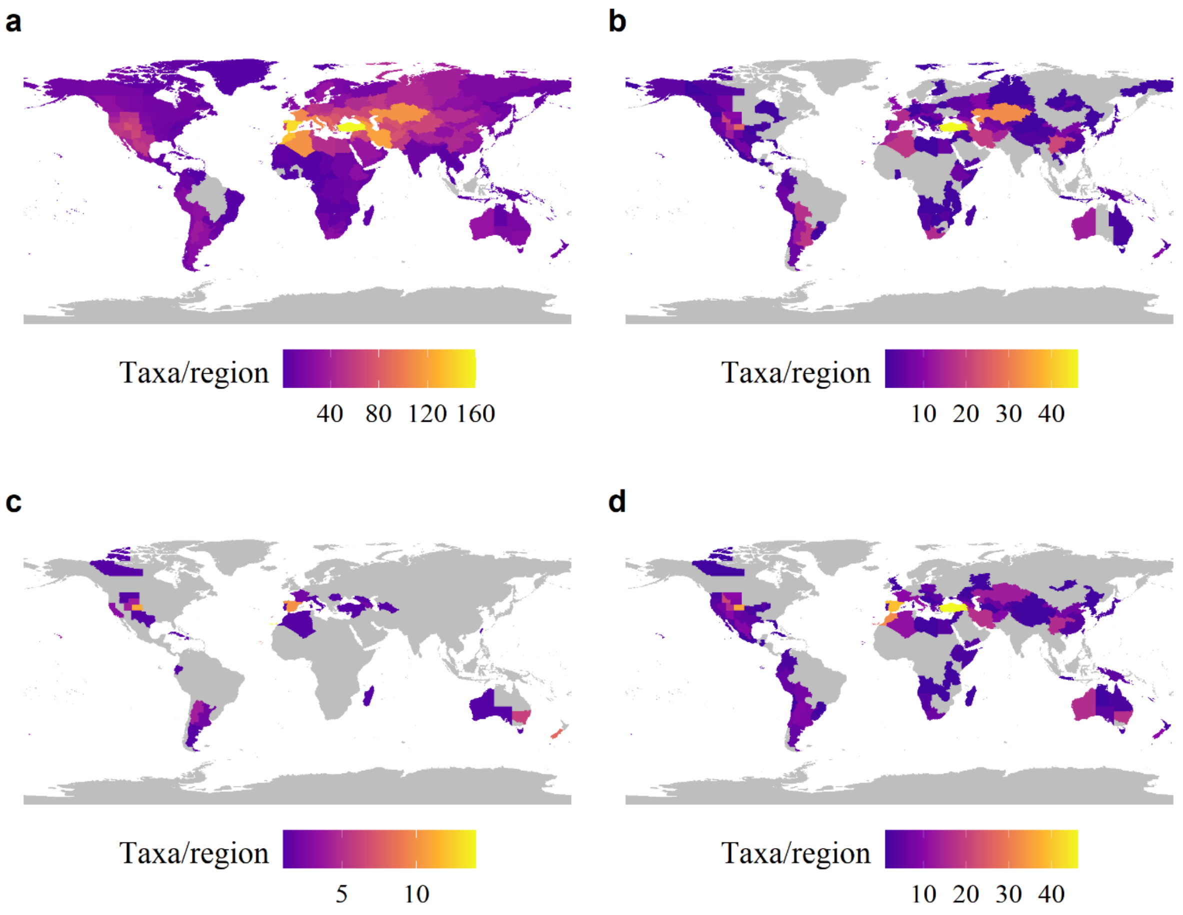

Figure 3. Global distribution of the 1,242 wild Brassicaceae species identified a) native distribution; b) distribution of species not conserved *ex situ*; c) threatened species; and d) endemic species. Grey areas represent regions where the populations are not present.

Figure3

Click here to access/download;Figure;Fig3.tiff

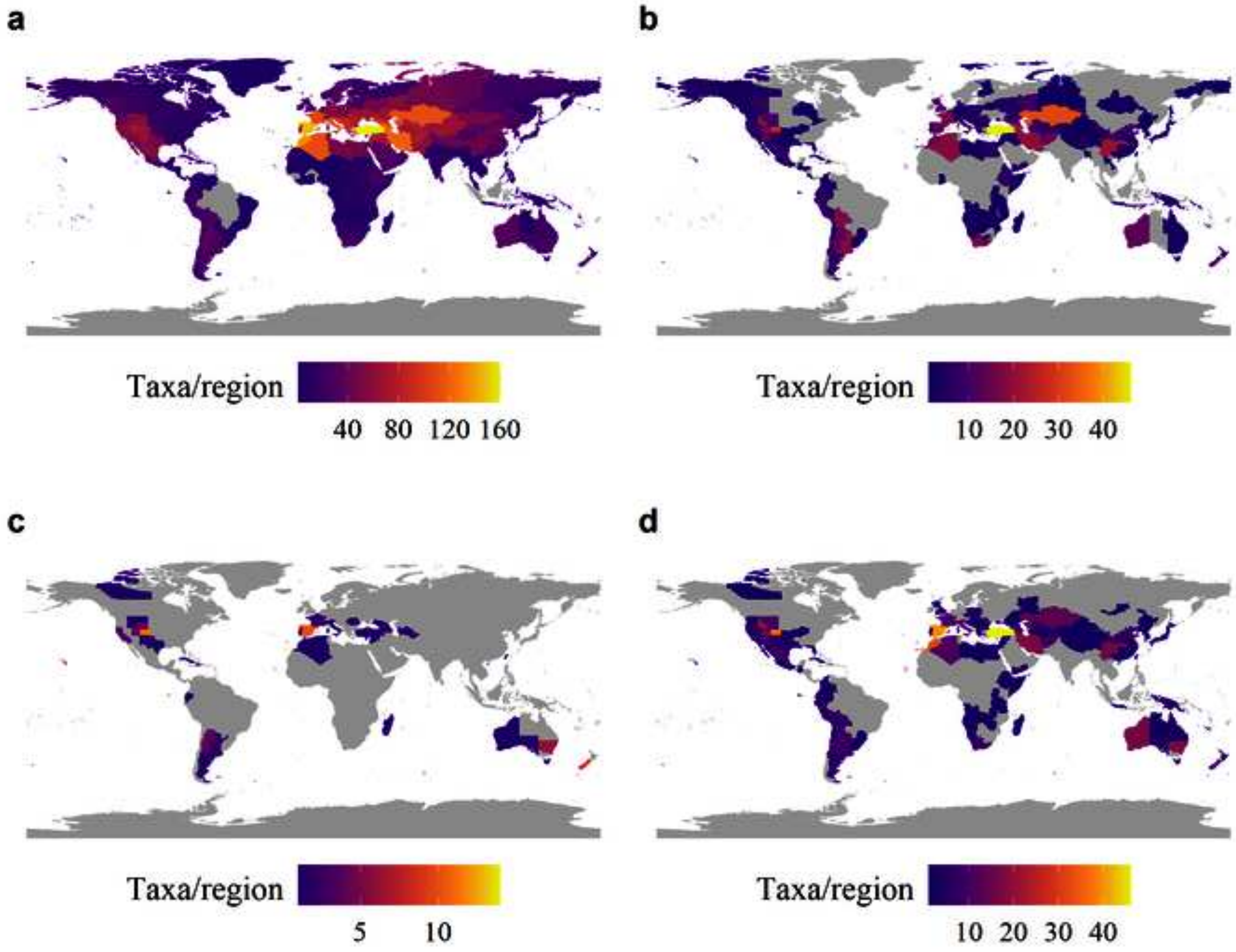

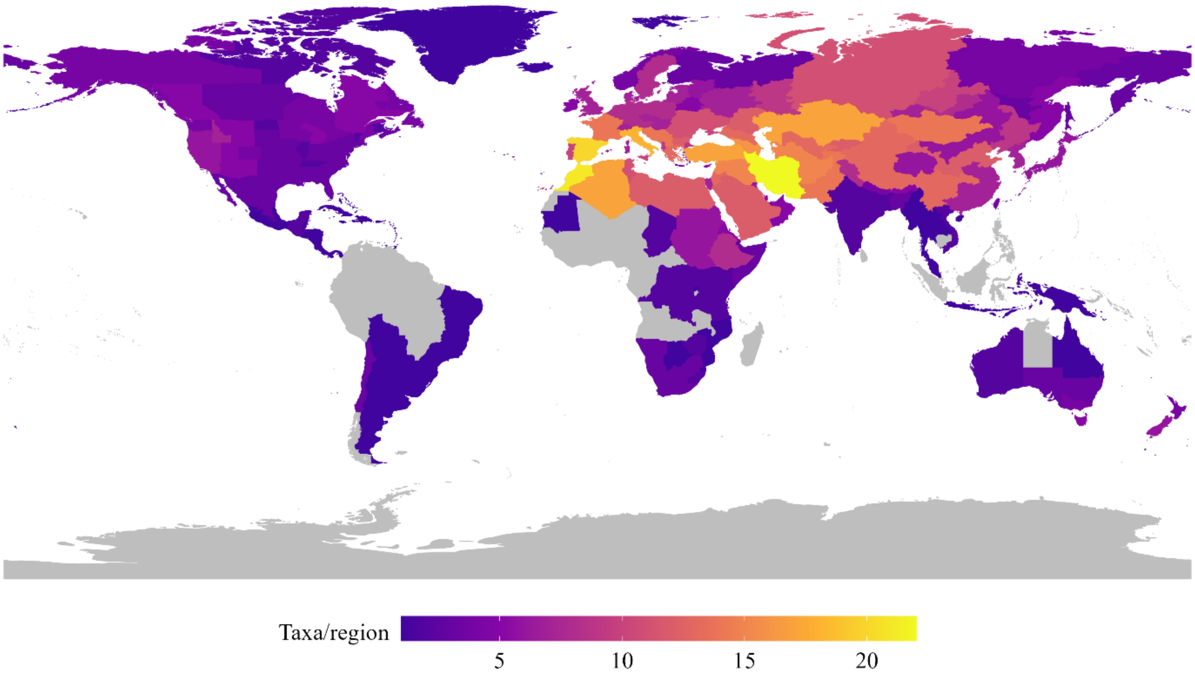

Figure 4. Global distribution of the new 103 estimated cross-compatible CWRs of Brassicaceae using phylogenetic distances. See Supplementary data for more details.

 Click here to access/download;Figure;Fig.4.tiff

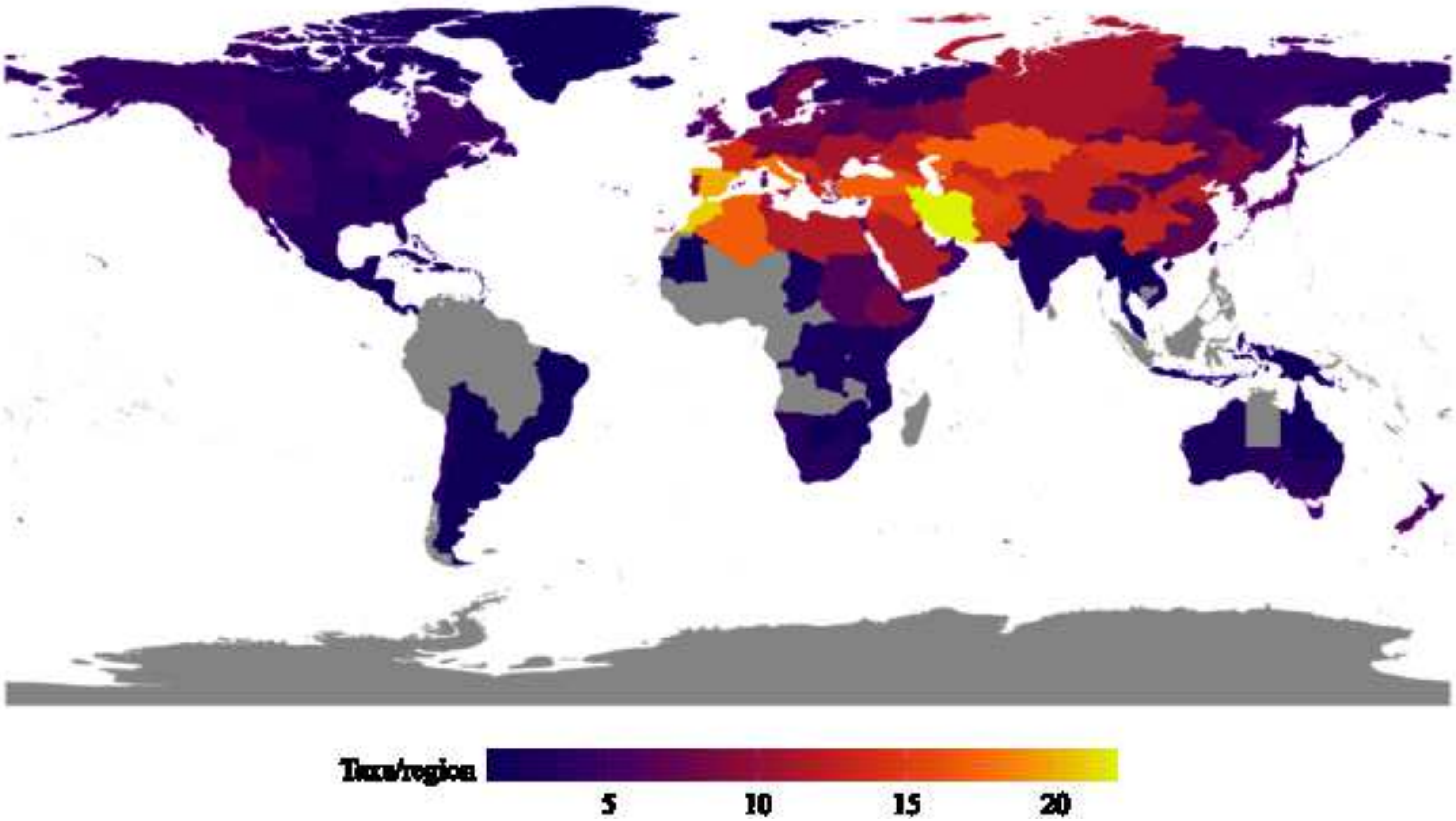

Click here to access/download
Supplementary Material
Supplementary_material_revised24.pdf

Personal cover

Click here to access/download;Personal Cover;ECL et al2023_Cover Letter.pdf

Kew Royal Botanic Gardens

Royal Botanic Gardens, Kew, Richmond, Surrey TW9 3AE
020 8332 5000 | kew.org | info@kew.org

Elena Castillo-Lorenzo
Royal Botanic Gardens, Kew, Wakehurst,
Ardingly, Haywards Heath, West Sussex
RH17 6TN
United Kingdom

December 2023

Dear Editor,

We are sending you the manuscript "*Current status of global conservation and characterisation of wild and cultivated Brassicaceae genetic resources: a gap analysis*" for consideration as a Research article, in GigaScience.

We have produced a large database of wild species (1242 spp.) with potential to be a crop wild relative (CWR) of cultivated Brassicaceae species. We identified what information is missing or needs to be improved to expand our knowledge and practices for using CWRs in breeding programmes. We gathered DNA sequence data and chromosome numbers for Brassicaeae species (to build a phylogenetic tree), conservation status information (present or not in *ex situ* seed banks as well as threat assessments) together with traits of interest for breeding as described in the literature.

The information gathered and analysed in this article will enhance the prioritisation of underrepresented Brassicaceae species to be evaluated for traits of interest for the development of climate change resilient crops, and obtaining their genetic sequences for further studies. Additionally, this information will serve as guidance for conservation programmes to prioritise hotspot areas and/or threatened species that urgently require collecting and conserving before they are lost to extinction. This will ensure not only the preservation of interesting plant genetic resources but also provide easy access to the material for future use.

Patron: His Royal Highness The Prince of Wales
Royal Botanic Gardens, Kew has exempt charitable status.

Our study, therefore, has a broad scope of global interest, suitable for scientists in plant species evolution, conservation, agriculture and breeding. GigaScience possess this diverse readership and scope, and will be appropriate to reach scientist globally.

We enclose the manuscript with 3 Figures, 1 Table and Supplementary material (1 Figure, 1 Table, 1 Database in excel with four sub-databases).

We have no other manuscripts under consideration or in press related to the current manuscript.

We declare no competing interests

The content of this manuscript has not been published previously or concurrently submitted for publication elsewhere. All authors have contributed significantly and are in agreement with the paper's content.

Yours faithfully,

Elena Castillo-Lorenzo (on behalf of all authors)
