# Supplementary material for: Current status of global conservation and characterisation of wild and cultivated Brassicaceae genetic resources
Source: Gigascience. 2024 Aug 7;13:giae050. doi: 10.1093/gigascience/giae050 (PMC11304946; doi:10.1093/gigascience/giae050)
Supplement: giae050_Supplemental_File [file giae050_supplemental_file.pdf]

Supplementary Figures and Tables

Current status of global conservation and characterisation of wild and cultivated Brassicaceae genetic resources

Castillo-Lorenzo, E.[α+], Breman, E.[α], Gómez Barreiro, P.[α], Viruel, J.[β]

[α] Royal Botanic Gardens, Kew, Wakehurst, Ardingly, Haywards Heath, West Sussex, RH17 6TN, UK

[β] Royal Botanic Gardens, Kew, Richmond, Surrey TW9 3AE, UK

[+] Corresponding author: e.castillolorenzo@kew.org

Fig. S1. Phylogenetic trees from each marker evaluated, a) matK, b) ITS, c) rbcL and d) trnL-F

Fig. S2. Proportion of number of entries in the NCBI for nine markers in the Brassicaceae family

Table S1. Classification of Brassicaceae wild species as CWRs combining two global databases

Supplementary data

Accessed here: https://doi.org/10.6084/m9.figshare.25002656

Fig S1. Phylogenetic trees from each marker evaluated, a) *mat*K, b) ITS, c) *rbc*L and d) *trn*L-F

Legend of the phylogenetic trees

Brassiceae tribe (Brassica, Crambe, Coincya, Diplotaxis, Eruca, Erucastrum, Sinapis, Moricandia, Rapistrum, Hirschfledia, Kremeriella**,** Orychophragmus)

Capsella – Camelina (Camelineae)

*__Cardamineae tribe__ (Barbarea, Nasturtium, Rorippa)

*__Descurainia__ (Descurainieae)

Eutrema (Eutremeae)

*__Isatis__ (Isatideae)

*__Lepidium__ (Lepidieae)

*__Physaria__ (Physarieae)

Sisymbrium (Sisymbrieae)

*tribes and genera included in Camelinodae I

a) *mat*K

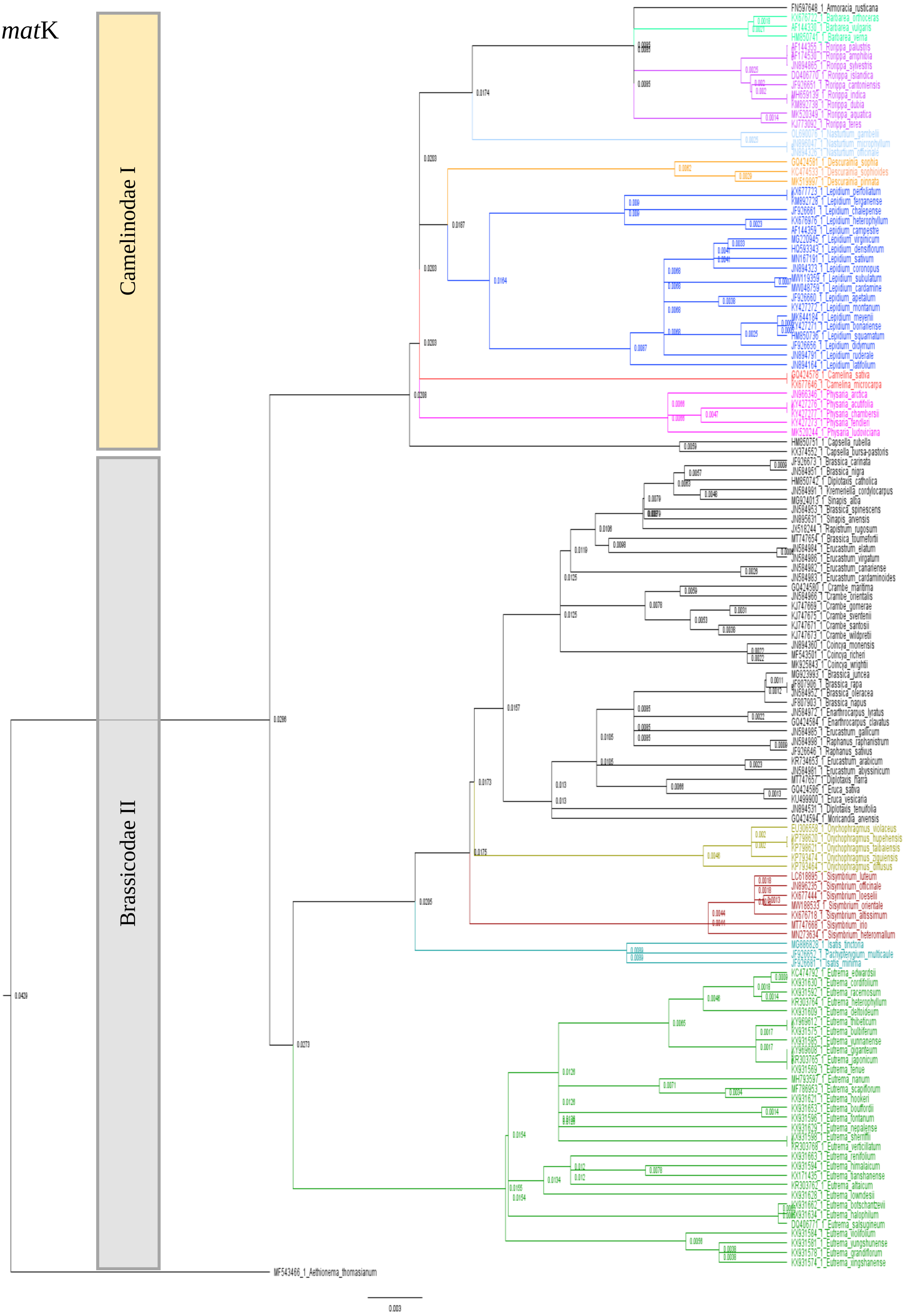

b) ITS

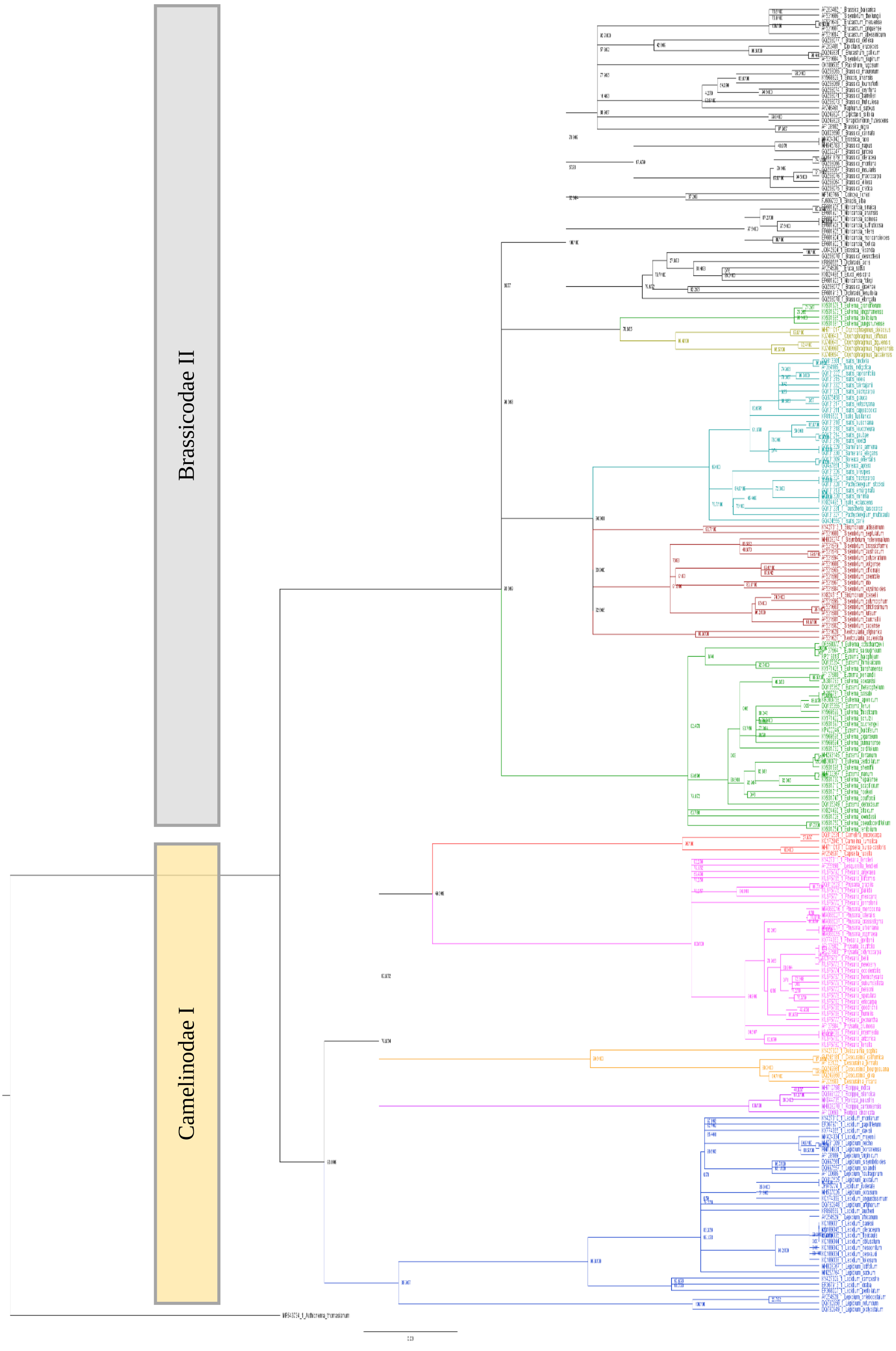

c) *rbc*L

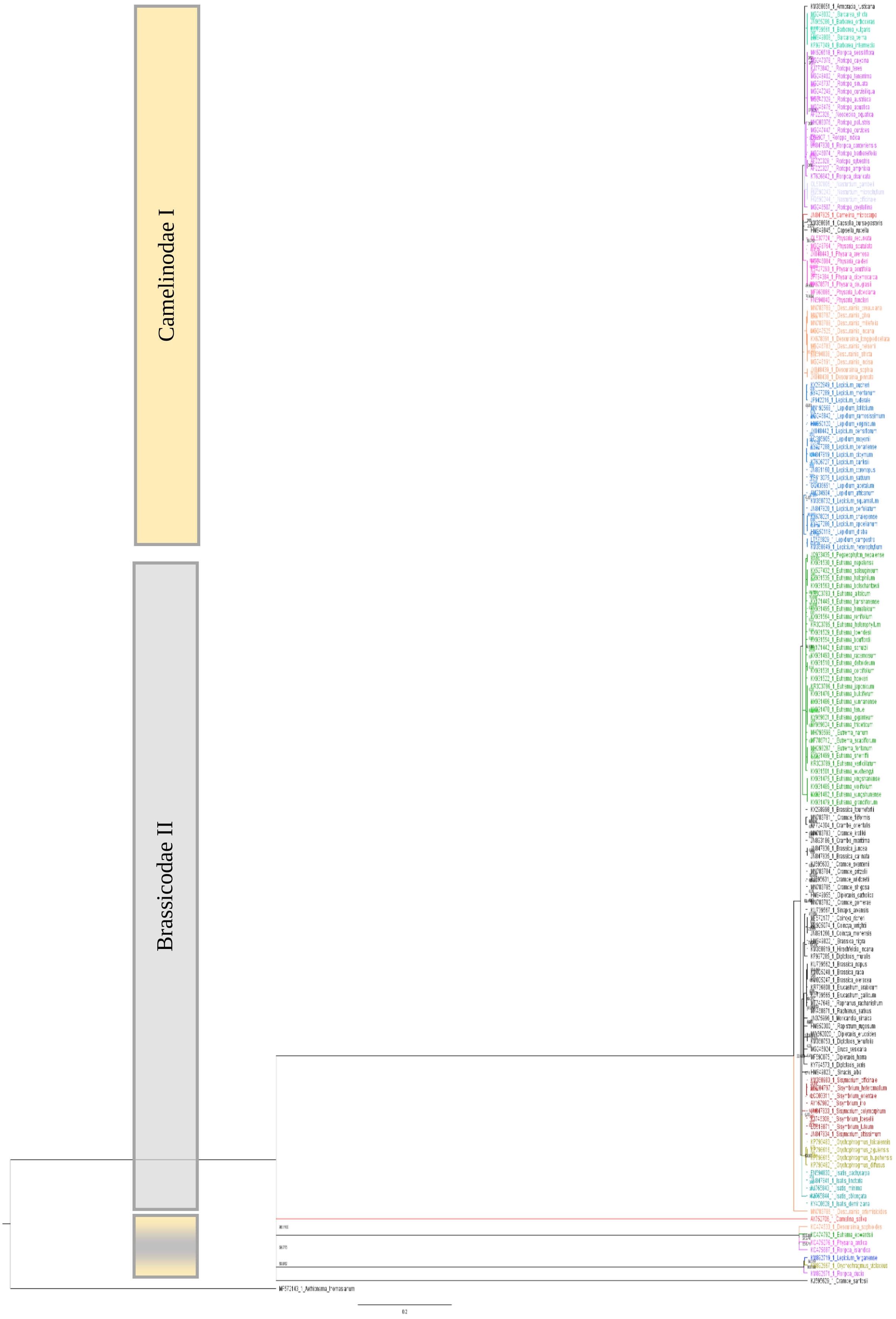

d) *trn*L-F

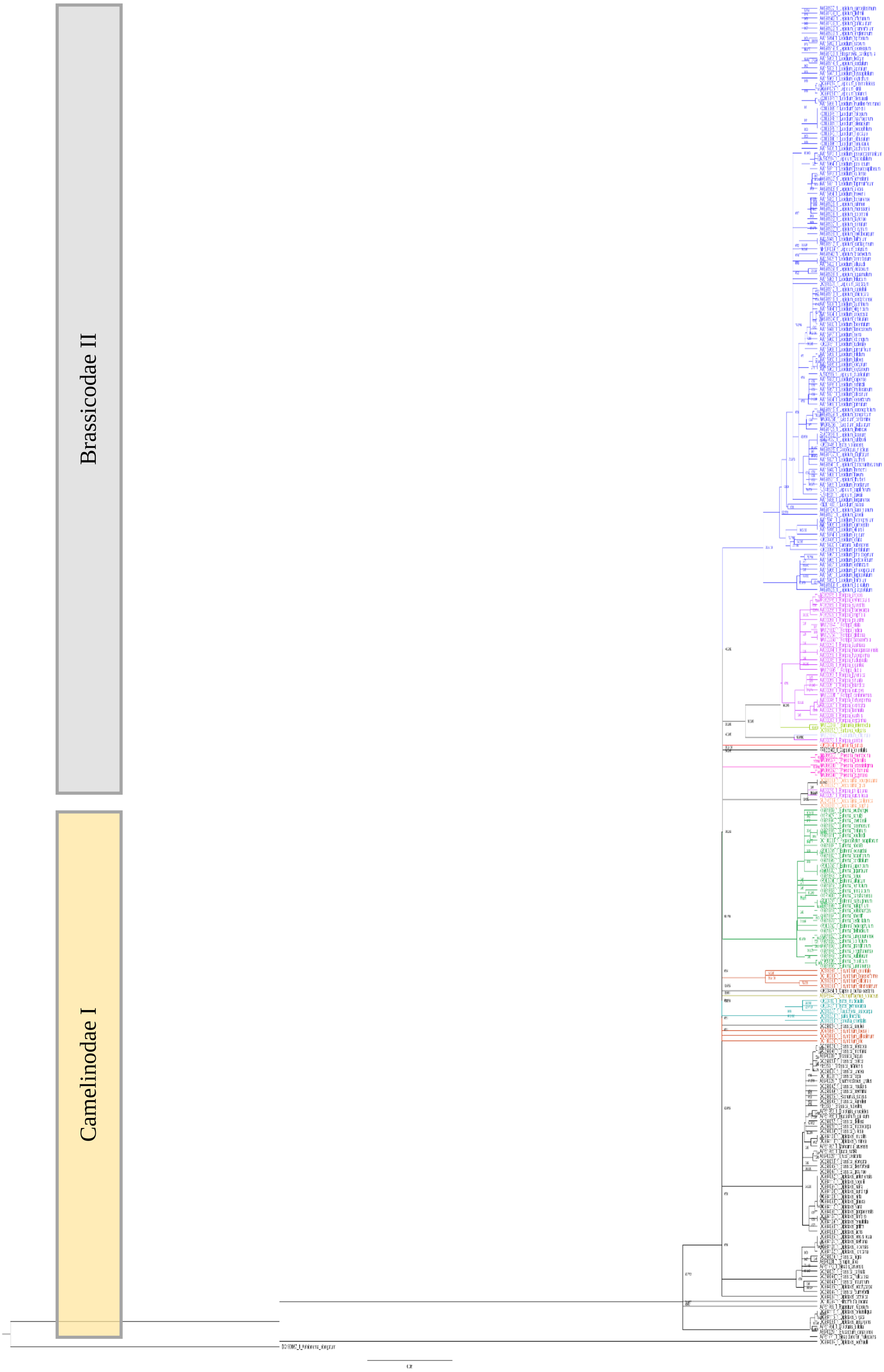

Fig S2. Proportion of number of entries in the NCBI (https://www.ncbi.nlm.nih.gov/, accessed on November 2022) for each marker in the Brassicaceae family

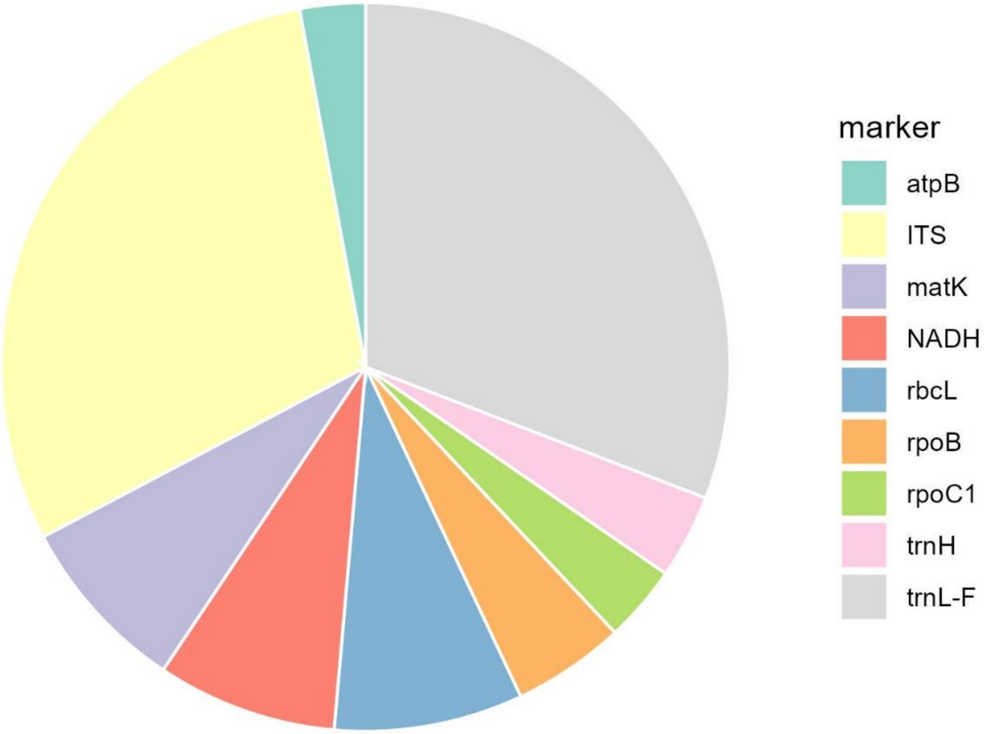

Table S1: Brassicaceae wild species classified as CWRs based on Gene Pool (GP) or Taxon Group (TG) classification. Data obtained for all the CWRs that had information available from USDA GRIN Global (https://npgsweb.ars-grin.gov/gringlobal/taxon/taxonomysearchcwr) and Harlan and De Wet CWR inventory (https://www.cwrdiversity.org/checklist, both accessed on December 2022).

**Scientific name**	**CWRs**	**Classification**
Armoracia rusticana	*A. macrocarpa*	TG4
	A. sisymbriodies	TG4
Barbarea verna<br>*Barbarea vulgaris*	*Barbarea* spp. (28 Accepted species)	TG4
Brassica carinata	*Brassica fruticulosa*	GP3
	Brassica juncea	GP2
	Brassica maurorum	GP3
	Brassica napus	GP3
	Brassica nigra	GP2
	Brassica oleracea	GP3
	Brassica rapa	GP2
	Camelina sativa	GP3
	Diplotaxis assurgens	GP3
	Diplotaxis tenuisiliqua	GP3
	Diplotaxis virgata	GP3
	Enarthrocarpus lyratus	GP3
	Erucastrum abyssinicum	GP3
	Erucastrum gallicum	GP3
	Orychophragmus violaceus	GP3
	Raphanus raphanistrum subsp. *sativus*	GP3
	Sinapis alba	GP3
	Sinapis arvensis	GP3
	Sinapis pubescens	GP3
Brassica juncea	*Brassica carinata*	GP2
	Brassica gravinae	GP3
	Brassica maurorum	GP3
	Brassica napus	GP2
	Brassica nigra	GP2
	Brassica oleracea	GP3
	Brassica oxyrrhina	GP3
	Brassica rapa	GP3
	Brassica spinescens	GP3
	Crambe hispanica subsp. *abyssinica*	GP3
	Diplotaxis berthautii	GP3
	Diplotaxis brevisiliqua	GP3

	Diplotaxis catholica	GP3
	Diplotaxis erucoides	GP3
	Diplotaxis glauca	GP3
	Diplotaxis harra	GP3
	Diplotaxis ibicensis	GP3
	Diplotaxis ilorcitana	GP3
	Diplotaxis muralis	GP3
	Diplotaxis siifolia	GP3
	Diplotaxis tenuifolia	GP3
	Diplotaxis virgata	GP3
	Enarthrocarpus lyratus	GP3
	Eruca pinnatifida	GP3
	Eruca vesicaria	GP3
	Erucastrum abyssinicum	GP3
	Erucastrum gallicum	GP3
	Erucastrum virgatum	GP3
	Moricandia arvensis	GP3
	Orychophragmus violaceus	GP3
	Raphanus raphanistrum	GP3
	Raphanus raphanistrum subsp. *sativus*	GP3
	Sinapis alba	GP3
	Sinapis arvensis	GP3
	Sinapis pubescens	GP3
	Trachystoma ballii	GP3
Brassica napus	*Brassica bourgeaui*	GP3
	Brassica carinata	GP3
	Brassica cretica	GP2
	Brassica deserti	GP3
	Brassica desnottesii	GP3
	Brassica dimorpha	GP3
	Brassica elongata	GP3
	Brassica fruticulosa	GP3
	Brassica fruticulosa subsp. *cossoniana*	GP3
	Brassica fruticulosa subsp. *fruticulosa*	GP3
	Brassica fruticulosa subsp. *numidica*	GP3
	Brassica fruticulosa subsp. *pomeliana*	GP3
	Brassica fruticulosa subsp. radicata	GP3
	Brassica gravinae	GP3
	Brassica hilarionis	GP3
	Brassica incana	GP3
	Brassica insularis	GP3
	Brassica juncea	GP2

	Brassica maurorum	GP3
	Brassica montana	GP3
	Brassica nigra	GP3
	Brassica oleracea	GP3
	Brassica rapa	GP2
	Brassica repanda	GP3
	Brassica souliei	GP3
	Brassica souliei subsp. amplexicaulis	GP3
	Brassica souliei subsp. souliei	GP3
	Capsella bursa-pastoris	GP3
	Crambe hispanica subsp. abyssinica	GP3
	Descurainia sophia	GP3
	Diplotaxis acris	GP3
	Diplotaxis brevisiliqua	GP3
	Diplotaxis catholica	GP3
	Diplotaxis erucoides	GP3
	Diplotaxis harra	GP3
	Diplotaxis ibicensis	GP3
	Diplotaxis muralis	GP3
	Diplotaxis siifolia	GP3
	Diplotaxis tenuifolia	GP3
	Diplotaxis viminea	GP3
	Enarthrocarpus lyratus	GP3
	Eruca pinnatifida	GP3
	Eruca vesicaria	GP3
	Erucastrum abyssinicum	GP3
	Erucastrum gallicum	GP2
	Hirschfeldia incana	GP3
	Moricandia arvensis	GP3
	Moricandia nitens	GP3
	Orychophragmus violaceus	GP3
	Physaria fendleri	GP3
	Raphanus raphanistrum	GP3
	Raphanus raphanistrum subsp. *sativus*	GP3
	Rapistrum rugosum	GP3
	Rorippa indica	GP3
	Rorippa islandica	GP3
	Sinapis alba	GP3
	Sinapis arvensis	GP3
	Sinapis pubescens	GP3
Brassica nigra	*Brassica carinata*	GP2
	Brassica fruticulosa	GP3
	Brassica juncea	GP2

	Brassica maurorum	GP2
	Brassica napus	GP3
	Brassica oleracea	GP2
	Brassica oxyrrhina	GP3
	Brassica procumbens	GP3
	Brassica rapa	GP3
	Brassica spinescens	GP3
	Coincya monensis	GP2
	Diplotaxis brevisiliqua	GP3
	Diplotaxis erucoides	GP3
	Diplotaxis ibicensis	GP3
	Diplotaxis ilorcitana	GP3
	Diplotaxis tenuifolia	GP3
	Erucastrum cardaminoides	GP3
	Hirschfeldia incana	GP3
	Orychophragmus violaceus	GP3
	Raphanus raphanistrum subsp. *sativus*	GP3
	Sinapis alba	GP3
	Sinapis arvensis	GP2
Brassica oleracea	*Armoracia rusticana*	GP3
	Brassica balearica	GP3
	Brassica bourgeaui	GP2
	Brassica carinata	GP3
	Brassica cretica	GP2
	Brassica cretica subsp. *aegaea*	GP2
	Brassica cretica subsp. *cretica*	GP2
	Brassica cretica subsp. *laconica*	GP2
	Brassica elongata	GP2
	Brassica elongata subsp. *elongata*	GP2
	Brassica elongata subsp. *integrifolia*	GP2
	Brassica elongata subsp. *pinnatifida*	GP2
	Brassica elongata subsp. *subscaposa*	GP2
	Brassica gravinae	GP3
	Brassica hilarionis	GP2
	Brassica incana	GP2
	Brassica insularis	GP2
	Brassica juncea	GP3
	Brassica macrocarpa	GP2
	Brassica maurorum	GP3
	Brassica montana	GP2
	Brassica napus	GP3
	Brassica nigra	GP2
	Brassica oxyrrhina	GP3
	Brassica rapa	GP2
	Brassica rupestris	GP2
	Brassica tournefortii	GP2

	Brassica villosa	GP2
	Capsella bursa-pastoris	GP3
	Coincya monensis	GP3
	Diplotaxis brevisiliqua	GP3
	Diplotaxis erucoides	GP3
	Diplotaxis ibicensis	GP3
	Diplotaxis ilorcitana	GP3
	Diplotaxis tenuifolia	GP3
	Enarthrocarpus lyratus	GP3
	Eruca pinnatifida	GP3
	Eruca vesicaria	GP3
	Erucastrum abyssinicum	GP3
	Hirschfeldia incana	GP3
	Moricandia arvensis	GP3
	Moricandia nitens	GP3
	Raphanus raphanistrum subsp. *sativus*	GP3
	Sinapis alba	GP3
	Sinapis arvensis	GP3
Brassica rapa	*Brassica aucheri*	GP3
	Brassica barrelieri	GP2
	Brassica bourgeaui	GP3
	Brassica carinata	GP2
	Brassica cretica	GP3
	Brassica deflexa	GP3
	Brassica elongata	GP2
	Brassica fruticulosa	GP3
	Brassica fruticulosa subsp. *fruticulosa*	GP3
	Brassica fruticulosa subsp. *numidica*	GP3
	Brassica fruticulosa subsp. *pomeliana*	GP3
	Brassica fruticulosa subsp. *radicata*	GP3
	Brassica gravinae	GP3
	Brassica incana	GP3
	Brassica insularis	GP3
	Brassica juncea	GP3
	Brassica macrocarpa	GP3
	Brassica maurorum	GP3
	Brassica montana	GP3
	Brassica napus	GP2
	Brassica nigra	GP3
	Brassica oleracea	GP2
	Brassica oxyrrhina	GP3
	Brassica spinescens	GP3
	Capsella bursa-pastoris	GP3
	Coincya monensis	GP3
	Crambe hispanica subsp. *abyssinica*	GP3

	Diplotaxis brevisiliqua	GP3
	Diplotaxis catholica	GP3
	Diplotaxis erucoides	GP3
	Diplotaxis ibicensis	GP3
	Diplotaxis ilorcitana	GP3
	Diplotaxis muralis	GP3
	Diplotaxis siifolia	GP3
	Diplotaxis tenuifolia	GP3
	Diplotaxis virgata	GP3
	Enarthrocarpus lyratus	GP3
	Eruca pinnatifida	GP3
	Eruca vesicaria	GP3
	Erucastrum canariense	GP3
	Erucastrum cardaminoides	GP3
	Erucastrum gallicum	GP2
	Orychophragmus violaceus	GP3
	Raphanus raphanistrum subsp. *sativus*	GP3
	Sinapis alba	GP3
	Sinapis arvensis	GP3
Crambe hispanica /<br>*C. hispanica subsp. abyssinica*	*Brassica juncea*<br>*Brassica napus*<br>*Brassica rapa*<br>*Crambe filiformis*<br>*Crambe hispanica* subsp. *abyssinica*	GP3<br>GP3<br>GP3<br>GP2<br>GP1
Diplotaxis tenuifolia	*Brassica elongata*<br>*Brassica juncea*<br>*Brassica nigra*<br>*Brassica oleracea*<br>*Brassica rapa*<br>*Erucastrum virgatum*	GP3<br>GP2<br>GP2<br>GP3<br>GP2<br>GP3
Eruca vesicaria	*Brassica juncea*<br>*Brassica napus*<br>*Brassica oleracea*<br>*Brassica rapa*<br>*Brassica repanda*<br>*Diplotaxis simplex*<br>*Diplotaxis tenuifolia*<br>*Eruca pinnatifida*<br>*Raphanus raphanistrum* subsp. *sativus*	GP3<br>GP3<br>GP3<br>GP3<br>GP3<br>GP2<br>GP2<br>GP1<br>GP3
Eutrema japonicum	*Eutrema tenue*<br>*E. yunnanense*	GP1<br>GP2
Isatis tinctoria	*Isatis* spp. (92 accepted species)	TG4
Lepidium meyenii	*L. bipinnatifidum*<br>*L. bonariense*<br>*L. quitense*	GP2<br>GP2<br>GP2

Lepidium sativum	*L. spinosum*	GP1
Nasturtium officinale	No information available	
Raphanus raphanistrum subsp. sativus	*Brassica carinata*<br>*Brassica fruticulosa*<br>*Brassica juncea*<br>*Brassica maurorum*<br>*Brassica napus*<br>*Brassica nigra*<br>*Brassica oleracea*<br>*Brassica oxyrrhina*<br>*Brassica rapa*<br>*Diplotaxis tenuifolia*<br>*Isatis tinctoria*<br>*Moricandia arvensis*<br>*Raphanus raphanistrum*<br>*Raphanus raphanistrum* subsp. *landra*<br>*Raphanus raphanistrum* subsp. *raphanistrum*<br>*Raphanus raphanistrum* subsp. *rostratus*<br>*Sinapis alba*<br>*Sinapis arvensis*<br>*Sinapis pubescens*	GP3<br>GP3<br>GP3<br>GP3<br>GP3<br>GP3<br>GP3<br>GP3<br>GP3<br>GP3<br>GP3<br>GP3<br>GP1<br>GP1<br>GP1<br>GP1<br>GP3<br>GP3<br>GP3
Rorippa indica	*Rorippa* spp. (86 Accepted species)	TG4
Sinapis alba	*Brassica carinata*<br>*Brassica juncea*<br>*Brassica napus*<br>*Brassica nigra*<br>*Brassica oleracea*<br>*Brassica rapa*<br>*Coincya monensis*<br>*Diplotaxis ilorcitana*<br>*Raphanus raphanistrum* subsp. *sativus*<br>*Sinapis alba subsp. alba*<br>*Sinapis alba subsp. dissecta*<br>*Sinapis alba subsp. mairei*<br>*Sinapis flexuosa*	GP3<br>GP3<br>GP3<br>GP3<br>GP3<br>GP3<br>GP2<br>GP3<br>GP3<br>GP1<br>GP1<br>GP1<br>GP1
Sisymbrium officinale	No information available	
