# Supplementary material for: Current status of global conservation and characterisation of wild and cultivated Brassicaceae genetic resources
Source: Gigascience. 2024 Aug 7;13:giae050. doi: 10.1093/gigascience/giae050 (PMC11304946; doi:10.1093/gigascience/giae050)
Supplement: giae050_Response_to_Reviewer_Comments_Original_Submission [file giae050_response_to_reviewer_comments_original_submission.pdf]

Author's Response To Reviewer Comments

GIGA-D-23-00376
Current status of global conservation and characterisation of wild and cultivated Brassicaceae genetic resources: a gap analysis
Elena Castillo-Lorenzo; Elinor Breman; Pablo Gómez Barreiro; Juan Viruel
GigaScience

RESPONSE TO THE REVIEWERS:

Reviewer #1:
This is an interesting manuscript about the use of phylogenetic distances as an alternative to classify Brassicaceae crop wild relatives (CWR) using the most recent genetic markers found in NCBI by the authors. The manuscript has interesting findings such as the 105 CWR probable classification as well as some traits as a complement to the analysis and geographical information. It is a great contribution to Brassicaceae conservation given that provides information about possible genetic closeness for further plant breeding. Nevertheless, there are some points to improve in the manuscript to gain readability below:

1.) The introduction section is really extensive. This seems more like an introduction of a thesis or a review and it is really hard to get the point of all the authors' efforts made in the analysis. I strongly recommend shortening this section and restating the objective part.
Resp: We agree that the introduction may be a bit extensive, but we think it is needed to understand the state-of-the-art and background of the review we have submitted, and it is also necessary to define the different topics the article explores such as the CWR classifications. We appreciate this feedback, and we used it to shorten the introduction to make it more readable.

2.) The authors stated that performed a gap analysis but this can be confusing for readers. The crop wild relatives gap analysis methodology proposed by Ramirez et al.,(2010) and subsequent work from Khoury and Castaneda used species distribution models as well as information from germplasm and herbaria. Thus, I suggest changing the gap analysis in the manuscript to avoid mistakes or confusion. For instance, instead of gap analysis would be like current geographic distribution and ex-situ conservation status
Resp: Thank you for your suggestion, we have replaced 'gap analysis' with 'current conservation status and geographic distribution' of CWRs in Brassicaceae in the title and in the text.

3.) I think most of the methods used in the manuscript are mostly well supported. For instance, the sequences collection, phylogenetic distances, and tree building are coherent but the following points require attention:
- Line 235 Global distribution and conservation gaps of Brassicaceae? or geographical conservation gaps of Brassicaceae
Resp: We have amended the text to Geographical conservation gaps.

- Line 433 and 434, the threshold method is not clear
Resp: We have modified the text on this section, and we hope it is clearer now. The new text is in lines 434-440

- Line 435, What were the criteria to choose those four markers?
Resp: We compiled the number of entries for different molecular markers available for Brassicaceae in NCBI: matK, ITS, rbcL, trnL-F, atpB, NADH, rpoB, rpoC1, trnH. We selected the four markers with the highest number of sequences available (matK, ITS, rbcL and trnL-F). We have included this in the methodology in addition to a Figure in Supplementary material showing the number of entries per marker (Fig S2).

- Line 444: Why GTR+I+G? Did the authors use a program such as JModelTest to obtain that nucleotide substitution model?
Resp: Yes, we used the MFP model, ModelFinder Plus, in IQTREE. We have now included this explanation in

Materials and Methods.

- Line 445, and 446: It is not clear why the longest if the alignment equalizes sequences long
Resp: Sorry, we meant the longest sequence before the alignment. We have amended this in the text.

- Line 448, What method did the authors use for the phylogenetic tree? ML, NJ?
Resp: We used ML as implemented in IQTREE. We have also added this in the text.

- Line 451 and 452. Why if it states about one final tree, the results have two trees?
Resp: Thank you for spotting this typo. We have amended this in the text.

4.) Why do authors use separate markers instead of combining them and making an integrative phylogenetic tree made with a blend of several markers?
Resp: Thanks for the comment We decided to keep the phylogenetic analysis separated for each molecular markers because of three reasons: first, some species had entries for matK but not for ITS and vice versa, and ITS had almost 100 species more than were present for matK sequences; second, there is incongruency between nuclear DNA and plastid DNA in phylogenetic reconstructions; and finally, not knowing if the samples obtained from the GenBank correspond to the same individuals for the two markers (there could be errors of identification that could create artifacts rather than increasing the resolution in the phylogenetic tree and we preferred to show them separately).

5.) Probably a combined approach can obtain better results at obtaining the matK and ITS resolution together.
Resp: Same explanation as above.

6.) I am just curious about Arabidopsis thaliana results (This is an important species for plant research)
Resp: Thank you for the comment. When selecting potential CWRs within the Brassicaceae family, Arabidopsis thaliana did not fit the criteria we followed. It is not a known conventional cross-compatible species with any crop in the family and it is not from any crop genus. In addition, it has been deeply studied and recently included in previous phylogenetic studies, we checked its relationship with our selection of crops, and it was distantly related. Finally, we consider it out of scope for our study that focuses on cross-compatibility with crops but of course A. thaliana is an important species in the family as a model plant.

7.) The trait characterization is interesting as a result and to complement the results in the materials there is no mention of why use it in the results
Resp: Thank you for the observation, we have incorporated text in the section 'Traits for breeding' to describe why we are gathering this information (results) and how we will use it in the future (discussion).

8.) I understand that Genesys was done to plot the areas with taxa worldwide but how was the data from IUCN in the Distribution and conservation data section used?
Resp: Thanks for this feedback, we have amended the text to make it clearer for the reader. POWO was used to plot the distribution of Brassicaceae taxa worldwide as mentioned in lines 474-478. Genesys was used to identify whether the species are conserved ex situ in any seed bank around the globe (Lines 483-484). To start creating a priority list for conservation, in addition, other criteria for the selection of species were included such as the threat status and whether they were an endemism. Being of high priority the ones that are not ex situ conserved elsewhere, they are threatened and / or endemic.

9.) How did authors filter Genesys information? Some of this information needs to be clear to be used.
Resp: We retrieved and filtered information from Genesys using the R package genesysr (reference is included in the text) and matched with our species list of Brassicaceae. We used this to know which species were present in global seed banks and which ones were not.

10.) I think the geographical section is really important and emphasizes the fact of the lack of work done in collecting.
Resp: Thank you very much for your comment, we hope these areas will be the starting point to guide collecting programs.

Reviewer #2:

The manuscript presents valuable insights into the identification of potential CWRs within the Brassicaceae family. The study's contribution to the understanding of conservation priorities and ex situ collection efforts is noteworthy. The focus on phylogenetic approaches to identify potential cross-compatible species is commendable. However, certain aspects require clarification and improvement.

* Line 61 - can you please further define what you mean by close phylogenetic relationship. I acknowledge two citations used, but I think it would be valuable to clarify the authors definition of a CWR. For example, is this the age of a clade? Or how much divergence has occurrence between the species? Etc.
Resp: Thanks for the comment, the phylogenetic tree is not dated, and it is not relative to time. The close relationship between branches is standardized to ‘1.0’ distance to the root in an ultrametic tree, the length of the branches represents the divergence between species, but not time. We have added a more detailed explanation in the methodology (Lines: 433-440).

* Line 75 - missing an "and" between Al-Shehbaz and Beilstein
o Same for line 81, between Hendricks and Kiefer
o And line 103, between Viruel and Kantar
o And line 128, between Kumar and Singh
Resp: Thank you for spotting this format typos. We have fixed them in the new version of the document.

* Line 115 - perhaps "and" makes more sense than "or" here.
Resp: Agreed, we have changed it.

* Line 241 - Which species are missing from ex situ collections in these places? Is there a list that can be used for collection priority? This would be super useful for germplasm curators.
o Same for the places lists on lines 244 and 245.
Resp: Thank you for the suggestion, we have updated the Supplementary Data (D1_GlobDistri_all) for all the species listed.

Table 1- You use the term CWP (crop wild phylorelatives) in your table caption but have not introduced this in your main text.
Resp: Thank you for spotting this, we will keep the table as Crop wild relatives (CWR).

Figure 2- All plots other than CWRs Crop Trust and Taxa go to the max number, why? The placement of a and b is a little hard to understand. Perhaps adding them to the left would be easier.
Resp: Thank you for the comment. We have changed the placement of the letters. Regarding the axis issue, TRY and the other two databases are in different scale on the y axis, since TRY had information on more than double the number of species. Thus, we could plot the CWRs inventory and USDA GRIN Global with the same y axis scale, CWRs inventory had information on fewer species than USDA GRIN Global.
Reviewer #3:

This manuscript investigates the phylogenetic distances between more than thousand Brassicaceae wild species that are potential relatives of crops. The study is following a methodology based on the alignment of existing plastid and nuclear sequences obtained from public databases. This methodology was previously tested more generally by one of the authors and it is here implemented for the specific crop wild relatives of the Brassicaceae family. The methodology has the advantage of providing a quantitative measurement of the phylogenetic distance between various taxa, which is considered a more valid proxy of the various taxa's crossability, than the existing knowledge can offer. The potential and limitations of this methodology are well described and the results offer a new list of potential GP2 CWRs. This gives very useful hints to prioritize, conserve and better study certain taxa that look more promising for crosses and pre-breeding. Compilation of information related to ploidy levels, useful traits, geographic distribution and status of conservation add more weight to the overall analysis of this potential list of CWRs of the Brassicaceae crops and offer many insights to better direct future efforts to improve knowledge and explore the potential for breeding.

The manuscript is therefore valuable and suitable for publication, with a proper methodology and conclusion.

My suggestion to further improve its value is to encourage highlighting the novel potential CWRs with a clearly identified list, and also attempting a wider analysis with more examples of potential newly discovered CWRs that were never considered before. I thought that also the maps of geographic density of the taxa would be more valuable if they could focus on the list of 105 new potential CWRs, that are the main result of this paper.
Resp: Thank you for your comment. We added the completed list of new potential CWRs that we have identified in this study. There are 103 now (after spotting one synonym and another that was previously cross-compatible, and thus not new CWR) and are mentioned in the text as before but represented in bold in Table 1 and the full detail and list in Supplementary Data, D1). Additionally, we have included their geographical distribution as Fig. 4.

The manuscript is clearly written, in a good language, with only very few misspellings or wrong verbal forms, of which I indicated some examples.
Resp: Thank you very much, we have amended the text and details are in the following comments.

Several public databases were consulted to extract data for this analysis. It would be useful to clearly indicate the correct titles of these databases and corresponding functioning URLs, which is not always the case.
Resp: Thanks to the reviewer for pointing out issues with some URLs, these have been amended now and we think all of them are correctly addressed with working URLs in the references.

Botanical names quoted for the first time mostly lack the respective authorities. It would be more precise to add them, depending on the editorial policy
Resp: We have amended the scientific names, including the authorities the first time the species is mentioned in the text, in Table 1 we haven't included the authorities for space reasons, but we have updated the supplementary data (with all species used in the study, and specified in the caption on Table 1) with an extra column of authors to incorporate this suggestion from the reviewer and editor.

I am adding a few relatively minor specific comments, line by line:

120: traits of interesting -->traits of interest
Resp: Thanks for spotting this typo, we have changed the word.

139-140: Brassicaceae publications of successful sexual crosses between crops and CWRs are very limited --> Resp: see comment below on line 431.

180-181: We distinguished successful conventional crosses…., between crosses… --> perhaps you mean "We distinguished successful conventional crosses…., from crosses ?
Resp: Thanks for spotting this typo, we have changed the word

182-183: A summary….included 265 taxa …is listed --> A summary….including 265 taxa …is listed.
Resp: Thanks for spotting this typo, we changed the word.

187: …there are no successful crosses reported. --> A successful conventional cross B. tournefortii x B. oleracea is reported in Warwick's Guide Part III, with reference Mattson (1988).
Resp: Thanks for spotting this, we have rephrased this in the text

202: Not clear why you estimate B. gravinae as likely cross-compatible with B. oleracea and B. rapa, based on phylogenetic distances, but these PDs are not shown in Table 1.
Resp: Thanks for spotting this, we were missing a line in Table 1 and now it has been amended. However, to keep Table 1 to a reasonable length in some cases we added Brassica spp., or Lepidium spp., when there was a long list of species within the same range of phylogenetic distances. We have included the new potential CWRs list in the Supplementary data.

202-203: Brassica oleracea is 2n=18 and only very rare and isolated cases of different polidy levels exist. Therefore, I think it is rather misleading to say that B. gravinae (2n=20) is likely compatible with the B. oleracea cole crops in general, since the cytotype 2n=20 is a real exception. On the other hand, your argument is good for crossing with B. rapa, which is 2n=10, but quoting here all the other very exceptional cases of different ploidy level of B. rapa is not so relevant in this context.
Resp: Thank you for the comment. We removed the rest of chromosome numbers for different cytotypes to make the statement clear and removed B. oleracea suggestion.

227: TRY database first mentioned here with no reference
Resp: We have now added a URL link to the database in the reference.

240-241: The greatest number of taxa missing from ex situ collections occur in Turkey, Kazakhstan and Colorado --> Perhaps worth explaining that you are using TDWG level 3 codes, otherwise it looks bizarre to put Colorado at the same level of national countries.
Resp: Thanks for the suggestion, we have now amended this here and in the methodology in lines 477-478.

291-293: I wonder why you make here the example of B. oleracea and B. carinata. The statement seems not too relevant, considering that you are not showing their Phylogenetic Distance in Table 1.
Resp: We discussed this example because B. oleracea is the progenitor of B. carinata, and crossing both species with successful and fertile hybrids is not easy according to the literature. We argue that the lack of compatibility could be due to differences in their chromosome numbers in addition to the large phylogenetic distance separating them.

332-333: B. oleracea is known to be relatively easy to cross with species of 'Brassica Section Brassica (n=9), not only cretica and incana, but also montana, macrocarpa, rupestris, etc. as demonstrated by Bothmer et al 1995 and several other papers.
Resp: Thank you, we were showing a few examples since in Table 1 there is all the information more detailed (as well as in the Supplementary), however we have added more examples in the text as well.

351: disploidy --> dysploidy
Resp: Thanks for spotting this typo, we changed the word

364: we have identified around 105 new potential CWRs for 18 crops --> I think this is the most valuable result of this study, but it is not included in the results section and it becomes evident with this quantitative detail only in the discussion. The reader wonders where to find these 105 new potential CWRs in the tables. I think that you should better highlight this list (perhaps bolding these new taxa in Table 1?). A few more examples of promising new CWRs that you have identified could also probably be made, although you correctly point out that other various under-investigated factors are implied before confirming their value as CWRs.
Resp: Thank you for your comment. We added the completed list of new potential CWR that we have identified in this study. There are 103 now (after spotting one synonym and other that was previously cross-compatible, and thus not new CWR) and are mentioned in the text as before but represented in bold in Table 1 and the full detail and list in Supplementary Data, D1). Additionally, we have included their geographical distribution as Fig. 4.

398 and following: the Fertile Crescent as it is commonly interpreted does not cover the Eastern Mediterranean area which is the site of hypothesized domestication of Brassica oleracea in Mabry et al 2021(and references therein). In the latter, what is intended is actually the Northeastern Mediterranean, i.e. the Greek speaking area of the first centuries before the current era. I think it is improper to associate B. oleracea to the Fertile Crescent, which is not known to host any of its closest CWRs in a native form.
Resp: Thanks for the suggestion, we changed the sentence to: “Both areas are two of the centers of origin highlighted by Vavilov and are a hotspot for plant biodiversity[48, 89]. For example, Brassica oleracea is thought to have been domesticated in Eastern Mediterranean[90]” in Lines 403-405.

431: 'We only used species with successful conventional crosses' --> I think an important reference is what you quoted in different context as [55], but not specifically for its Part III, which is relevant here: Warwick

SI, Francis A, Gugel RK. 2009. Guide to wild germplasm of Brassica and allied crops (tribe Brassiceae, Brassicaceae). 3rd ed. Part III - Interspecific and Intergeneric Hybridization in the Tribe Brassiceae (Cruciferae).
Resp: We added the reference of Warwick et al., 2009 in the methodology and in lines 124-125, as suggested by the reviewer in addition to the other literature we checked to identify cross-compatible CWRs (listed on Table 1 under (Y)).

435: NCBI --> there is no reference/URL given about this non spelled acronym
Resp: We have now added a URL link to the database and the definition of the acronym.

436-37: "The Wild Germplasm of Brassica [37]" does not seem to correspond to reference 37: Warwick et al. 2009. You say this was "accessed on November 2022". What is the URL of this web site?
Resp: Thanks for spotting this mistake. The correct reference is [55 in the previous version, now is ref 43] Warwick et al., 2009 Guide to wild germplasm of Brassica and allied crops (tribe Brassiceae, Brassicaceae). Part II: Chromosome number. Which was previously published in a CD-ROM as a database (http://www.brassica.info) but it was migrated in late 2023. We have changed the correct reference now.

437: Brassibase [9] (accessed on November 2022)--> the URL is missing.
Resp: We have added the URL in the references.

438: Plant DNA C-values database [93] (accessed on November 2022) --> the URL is missing
Resp: We have added the URL in the references.

438-39: plant CCDB database [95] (accessed on November 2022) --> the URL is missing and in case you mean http://ccdb.tau.ac.il/, please note that this site is not working, which is rather frustrating. Hopefully you can point at a functional web site.
Resp: We have added the URL in the references. In our case, the following address is working and functional: https://taux.evolseq.net/CCDB_web

455: "USDA GRIN Global [94]" --> GRIN Global is a database platform, i.e. a software that can be adopted by genebanks around the world. It is not a searchable database per se. You are possibly confusing it with the USDA GRIN Taxonomy database. To eliminate ambiguities, you need to indicate the URL and the correct title of what you have browsed.
Resp: We have added the URL to avoid confusion: https://npgsweb.ars-grin.gov/gringlobal/taxon/taxonomysearchcwr and we did search on the 'Query Crop Relatives in GRIN-Global' section and downloaded a file for the Brassicaceae family.

456: "inventory of the Global Diversity Trust" --> Surely this inventory has a more specific name. Possibly you are referring to the "Harlan and De Wet CWR inventory", which is however no longer maintained. You should use the proper name and indicate the URL of the web site that you accessed to avoid ambiguity.
Resp: We have added the URL and changed the name of the inventory to 'Harlan and De Wet CWR' here and elsewhere in the text, as the reviewer correctly pointed.

458: "TRY database" --> It would be useful to include the URL and date of access.
Resp: We have now added the URL as you and other reviewers suggested.

462 and 466: Every time that you indicate that you accessed a web site, you should include the URL, that is for World Online and BGCI.
Resp: We have now added the URL as you and other reviewers suggested.

722: Reference 94: the URL of the web site should be added.
Resp: We have added the URL to avoid confusion in the references: https://npgsweb.ars-grin.gov/gringlobal/taxon/taxonomysearchcwr

779 and following: The cross between Brassica oleracea and Brassica cretica has been successful, as you say in line 332. Therefore, you should indicate (Y) after B. cretica in this cross.

Resp: Thank you for spotting this missed (Y), we amended on the table.

Figure 3: These maps are very interesting. However, you have built them based on the starting list of 1,242 species. It is understandable, since many of these still need a lot of investigation and offer potential. However, after your study, your main result is a new list of CWR with a high potential to hybridize with crops. This becomes a priority list from a utilitarian point of view. Thus, it would be very interesting to also see maps dedicated to the new priority list. This would be a great help for prioritization of action.
Resp: Thank you for your comment. We added the completed list of new potential CWR that we have identified in this study. There are 103 now (after spotting one synonym and other that was previously cross-compatible, and thus not new CWR) and are mentioned in the text as before but represented in bold in Table 1 and the full detail and list in Supplementary Data, D1). Additionally, we have included their geographical distribution as Fig. 4.

Table S1 legenda: it is not clear from where you got the data of table S1. When you say "Global Crop Trust Diversity", you probably intend 'Global Crop Diversity Trust' instead, but this is not a well identified web site, it is just the name of an organization. And by 'USDA Global-GRIN', you probably mean 'USDA GRIN-Global', which is not an identified web site.
Resp: We have amended and added the URL every time we mention a database, so this has been modified on Table S1 as well. And we have changed the concept to USDA GRIN-Global.
