# Supplementary material for: Current status of global conservation and characterisation of wild and cultivated Brassicaceae genetic resources
Source: Gigascience. 2024 Aug 7;13:giae050. doi: 10.1093/gigascience/giae050 (PMC11304946; doi:10.1093/gigascience/giae050)
Supplement: giae050_Response_to_Reviewer_Comments_Revision_1 [file giae050_response_to_reviewer_comments_revision_1.pdf]

Author's Response To Reviewer Comments

Dear Editor,
I have submitted the last version of the manuscript in Word. With the following changes
1- I have added the citations and text for the Supplemetary data in GigaDB and Figshare.
2- I have removed the url links through the text and placed them only on the references
3- I included the ORCID number in the tittle page.
4- The abstract is now structured as Background, Results and Conclusions
5- I have re-order the last sections after the discussion section and placed the references at the very end
6- I removed the highlighted text
7- the uploaded document is in Word (.doc) format
8- I think all the references and style is according to the instructions to authors stated on the website.
I have added a sentence to the Acknowledgments section, there is a project of crop wild relatives that will help paying for the open access fees of this manuscript and I have acknowledged them. I hope this is okay.

Thank you very much!
Best,
Elena
