# Supplementary material for: Current status of global conservation and characterisation of wild and cultivated Brassicaceae genetic resources
Source: Gigascience. 2024 Aug 7;13:giae050. doi: 10.1093/gigascience/giae050 (PMC11304946; doi:10.1093/gigascience/giae050)
Supplement: giae050_Reviewer_1_Report_Original_Submission — Chrystian C. Sosa -- 2/19/2024 Reviewed [file giae050_reviewer_1_report_original_submission.pdf]

Reviewer Report

Title: Current status of global conservation and characterisation of wild and cultivated Brassicaceae genetic resources

Version: Original Submission **Date:** 2/19/2024

Reviewer name: Chrystian C. Sosa

Reviewer Comments to Author:

This is an interesting manuscript about the use of phylogenetic distances as an alternative to classify Brassicaceae crop wild relatives (CWR) using the most recent genetic markers found in NCBI by the authors. The manuscript has interesting findings such as the 105 CWR probable classification as well as some traits as a complement to the analysis and geographical information. It is a great contribution to Brassicaceae conservation given that provides information about possible genetic closeness for further plant breeding. Nevertheless, there are some points to improve in the manuscript to gain readability below:

1.) The introduction section is really extensive. This seems more like an introduction of a thesis or a review and it is really hard to get the point of all the authors' efforts made in the analysis. I strongly recommend shortening this section and restating the objective part.

2.) The authors stated that performed a gap analysis but this can be confusing for readers. The crop wild relatives gap analysis methodology proposed by Ramirez et al.,(2010) and subsequent work from Khoury and Castaneda used species distribution models as well as information from germplasm and herbaria. Thus, I suggest changing the gap analysis in the manuscript to avoid mistakes or confusion. For instance, instead of gap analysis would be like current geographic distribution and ex-situ conservation status

3.) I think most of the methods used in the manuscript are mostly well supported. For instance, the sequences collection, phylogenetic distances, and tree building are coherent but the following points require attention:

- Line 235 Global distribution and conservation gaps of Brassicaceae? or geographical conservation gaps of Brassicaceae
- Line 433 and 434, the threshold method is not clear
- Line 435, What were the criteria to choose those four markers?
- Line 444: Why GTR+I+G? Did the authors use a program such as JModelTest to obtain that nucleotide substitution model?
- Line 445, and 446: It is not clear why the longest if the alignment equalizes sequences long
- Line 448, What method did the authors use for the phylogenetic tree? ML, NJ?
- Line 451 and 452. Why if it states about one final tree, the results have two trees?

4.) Why do authors use separate markers instead of combining them and making an integrative phylogenetic tree made with a blend of several markers?

5.) Probably a combined approach can obtain better results at obtaining the matK and ITS resolution together.

6.) I am just curious about Arabidopsis thaliana results (This is an important species for plant research)

7.) The trait characterization is interesting as a result and to complement the results in the materials there is no mention of why use it in the results
8.) I understand that Genesys was done to plot the areas with taxa worldwide but how was the data from IUCN in the Distribution and conservation data section used?
9.) How did authors filter Genesys information? Some of this information needs to be clear to be used.
10.) I think the geographical section is really important and emphasizes the fact of the lack of work done in collecting.

Methods

Are the methods appropriate to the aims of the study, are they well described, and are necessary controls included? Choose an item.

Conclusions

Are the conclusions adequately supported by the data shown? Choose an item.

Reporting Standards

Does the manuscript adhere to the journal's guidelines on [minimum standards of reporting?]() Choose an item.

Choose an item.

Statistics

Are you able to assess all statistics in the manuscript, including the appropriateness of statistical tests used? Choose an item.

Quality of Written English

Please indicate the quality of language in the manuscript: Choose an item.

Declaration of Competing Interests

Please complete a declaration of competing interests, considering the following questions:

- Have you in the past five years received reimbursements, fees, funding, or salary from an organisation that may in any way gain or lose financially from the publication of this manuscript, either now or in the future?
- Do you hold any stocks or shares in an organisation that may in any way gain or lose financially from the publication of this manuscript, either now or in the future?
- Do you hold or are you currently applying for any patents relating to the content of the manuscript?

- Have you received reimbursements, fees, funding, or salary from an organization that holds or has applied for patents relating to the content of the manuscript?
- Do you have any other financial competing interests?
- Do you have any non-financial competing interests in relation to this paper?

If you can answer no to all of the above, write 'I declare that I have no competing interests' below. If your reply is yes to any, please give details below.

I declare that I have no competing interest

I agree to the open peer review policy of the journal. I understand that my name will be included on my report to the authors and, if the manuscript is accepted for publication, my named report including any attachments I upload will be posted on the website along with the authors' responses. I agree for my report to be made available under an Open Access Creative Commons CC-BY license (http://creativecommons.org/licenses/by/4.0/). I understand that any comments which I do not wish to be included in my named report can be included as confidential comments to the editors, which will not be published.

Choose an item.

To further support our reviewers, we have joined with Publons, where you can gain additional credit to further highlight your hard work (see: https://publons.com/journal/530/gigascience). On publication of this paper, your review will be automatically added to Publons, you can then choose whether or not to claim your Publons credit. I understand this statement.

Yes Choose an item.
