# Supplementary material for: Current status of global conservation and characterisation of wild and cultivated Brassicaceae genetic resources
Source: Gigascience. 2024 Aug 7;13:giae050. doi: 10.1093/gigascience/giae050 (PMC11304946; doi:10.1093/gigascience/giae050)
Supplement: giae050_Reviewer_2_Report_Original_Submission — MAKENZIE Mabry -- 2/23/2024 Reviewed [file giae050_reviewer_2_report_original_submission.pdf]

Reviewer Report

Title: Current status of global conservation and characterisation of wild and cultivated Brassicaceae genetic resources

Version: Original Submission **Date:** 2/23/2024

Reviewer name: MAKENZIE Mabry

Reviewer Comments to Author:

The manuscript presents valuable insights into the identification of potential CWRs within the Brassicaceae family. The study's contribution to the understanding of conservation priorities and ex situ collection efforts is noteworthy. The focus on phylogenetic approaches to identify potential cross-compatible species is commendable. However, certain aspects require clarification and improvement.

* Line 61 - can you please further define what you mean by close phylogenetic relationship. I acknowledge two citations used, but I think it would be valuable to clarify the authors definition of a CWR. For example, is this the age of a clade? Or how much divergence has occurrence between the species? etc.
* Line 75 - missing an "and" between Al-Shehbaz and Beilstein
o Same for line 81, between Hendricks and Kiefer
o And line 103, between Viruel and Kantar
o And line 128, between Kumar and Singh
* Line 115 - perhaps "and" makes more sense than "or" here.
* Line 241 - Which species are missing from ex situ collections in these places? Is there a list that can be used for collection priority? This would be super useful for germplasm curators.
o Same for the places lists on lines 244 and 245.

Table 1- You use the term CWP (crop wild phylorelatives) in your table caption but have not introduced this in your main text.

Figure 2- All plots other than CWRs Crop Trust and Taxa go to the max number, why? The placement of a and b is a little hard to understand. Perhaps adding them to the left would be easier.

Methods

Are the methods appropriate to the aims of the study, are they well described, and are necessary controls included? Choose an item.

Conclusions

Are the conclusions adequately supported by the data shown? Choose an item.

Reporting Standards

Does the manuscript adhere to the journal's guidelines on minimum standards of reporting? Choose an item.

Choose an item.

Statistics

Are you able to assess all statistics in the manuscript, including the appropriateness of statistical tests used? Choose an item.

Quality of Written English

Please indicate the quality of language in the manuscript: Choose an item.

Declaration of Competing Interests

Please complete a declaration of competing interests, considering the following questions:

- Have you in the past five years received reimbursements, fees, funding, or salary from an organisation that may in any way gain or lose financially from the publication of this manuscript, either now or in the future?
- Do you hold any stocks or shares in an organisation that may in any way gain or lose financially from the publication of this manuscript, either now or in the future?
- Do you hold or are you currently applying for any patents relating to the content of the manuscript?
- Have you received reimbursements, fees, funding, or salary from an organization that holds or has applied for patents relating to the content of the manuscript?
- Do you have any other financial competing interests?
- Do you have any non-financial competing interests in relation to this paper?

If you can answer no to all of the above, write 'I declare that I have no competing interests' below. If your reply is yes to any, please give details below.

I declare that I have no competing interests

I agree to the open peer review policy of the journal. I understand that my name will be included on my report to the authors and, if the manuscript is accepted for publication, my named report including any attachments I upload will be posted on the website along with the authors' responses. I agree for my report to be made available under an Open Access Creative Commons CC-BY license (http://creativecommons.org/licenses/by/4.0/). I understand that any comments which I do not wish to be included in my named report can be included as confidential comments to the editors, which will not be published.

Choose an item.

To further support our reviewers, we have joined with Publons, where you can gain additional credit to further highlight your hard work (see: https://publons.com/journal/530/gigascience). On publication of this paper, your review will be automatically added to Publons, you can then choose whether or not to claim your Publons credit. I understand this statement.

Yes Choose an item.
