# Supplementary material for: Current status of global conservation and characterisation of wild and cultivated Brassicaceae genetic resources
Source: Gigascience. 2024 Aug 7;13:giae050. doi: 10.1093/gigascience/giae050 (PMC11304946; doi:10.1093/gigascience/giae050)
Supplement: giae050_Reviewer_3_Report_Original_Submission — Lorenzo Maggioni -- 2/28/2024 Reviewed [file giae050_reviewer_3_report_original_submission.pdf]

Reviewer Report

Title: Current status of global conservation and characterisation of wild and cultivated Brassicaceae genetic resources

Version: Original Submission **Date:** 2/28/2024

Reviewer name: Lorenzo Maggioni

Reviewer Comments to Author:

This manuscript investigates the phylogenetic distances between more than thousand Brassicaceae wild species that are potential relatives of crops. The study is following a methodology based on the alignment of existing plastid and nuclear sequences obtained from public databases. This methodology was previously tested more generally by one of the authors and it is here implemented for the specific crop wild relatives of the Brassicaceae family. The methodology has the advantage of providing a quantitative measurement of the phylogenetic distance between various taxa, which is considered a more valid proxy of the various taxa's crossability, than the existing knowledge can offer. The potential and limitations of this methodology are well described and the results offer a new list of potential GP2 CWRs. This gives very useful hints to prioritize, conserve and better study certain taxa that look more promising for crosses and pre-breeding.
Compilation of information related to ploidy levels, useful traits, geographic distribution and status of conservation add more weight to the overall analysis of this potential list of CWRs of the Brassicaceae crops and offer many insights to better direct future efforts to improve knowledge and explore the potential for breeding.
The manuscript is therefore valuable and suitable for publication, with a proper methodology and conclusion.
My suggestion to further improve its value is to encourage highlighting the novel potential CWRs with a clearly identified list, and also attempting a wider analysis with more examples of potential newly discovered CWRs that were never considered before. I thought that also the maps of geographic density of the taxa would be more valuable if they could focus on the list of 105 new potential CWRs, that are the main result of this paper.
The manuscript is clearly written, in a good language, with only very few misspellings or wrong verbal forms, of which I indicated some examples.
Several public databases were consulted to extract data for this analysis. It would be useful to clearly indicate the correct titles of these databases and corresponding functioning URLs, which is not always the case.
Botanical names quoted for the first time mostly lack the respective authorities. It would be more precise to add them, depending on the editorial policy
I am adding a few relatively minor specific comments, line by line:
120: traits of interesting -->traits of interest
139-140: Brassicaceae publications of successful sexual crosses between crops and CWRs are very limited --> see comment below on line 431.

180-181: We distinguished successful conventional crosses…., between crosses… --> perhaps you mean "We distinguished successful conventional crosses…., from crosses ?

182-183: A summary….included 265 taxa …is listed --> A summary….including 265 taxa …is listed.

187: …there are no successful crosses reported. --> A successful conventional cross B. tournefortii x B. oleracea is reported in Warwick's Guide Part III, with reference Mattson (1988).

202: Not clear why you estimate B. gravinae as likely cross-compatible with B. oleracea and B. rapa, based on phylogenetic distances, but these PDs are not shown in Table 1.

202-203: Brassica oleracea is 2n=18 and only very rare and isolated cases of different polidy levels exist. Therefore, I think it is rather misleading to say that B. gravinae (2n=20) is likely compatible with the B. oleracea cole crops in general, since the cytotype 2n=20 is a real exception. On the other hand, your argument is good for crossing with B. rapa, which is 2n=10, but quoting here all the other very exceptional cases of different ploidy level of B. rapa is not so relevant in this context.

227: TRY database first mentioned here with no reference

240-241: The greatest number of taxa missing from ex situ collections occur in Turkey, Kazakhstan and Colorado --> Perhaps worth explaining that you are using TDWG level 3 codes, otherwise it looks bizarre to put Colorado at the same level of national countries.

291-293: I wonder why you make here the example of B. oleracea and B. carinata. The statement seems not too relevant, considering that you are not showing their Phylogenetic Distance in Table 1.

332-333: B. oleracea is known to be relatively easy to cross with species of 'Brassica Section Brassica (n=9), not only cretica and incana, but also montana, macrocarpa, rupestris, etc. as demonstrated by Bothmer et al 1995 and several other papers.

351: disploidy --> dysploidy

364: we have identified around 105 new potential CWRs for 18 crops --> I think this is the most valuable result of this study, but it is not included in the results section and it becomes evident with this quantitative detail only in the discussion. The reader wonders where to find these 105 new potential CWRs in the tables. I think that you should better highlight this list (perhaps bolding these new taxa in Table 1?). A few more examples of promising new CWRs that you have identified could also probably be made, although you correctly point out that other various under-investigated factors are implied before confirming their value as CWRs.

398 and following: the Fertile Crescent as it is commonly interpreted does not cover the Eastern Mediterranean area which is the site of hypothesized domestication of Brassica oleracea in Mabry et al 2021(and references therein). In the latter, what is intended is actually the Northeastern Mediterranean, i.e. the Greek speaking area of the first centuries before the current era. I think it is improper to associate B. oleracea to the Fertile Crescent, which is not known to host any of its closest CWRs in a native form.

431: 'We only used species with successful conventional crosses' --> I think an important reference is what you quoted in different context as [55], but not specifically for its Part III, which is relevant here: Warwick SI, Francis A, Gugel RK. 2009. Guide to wild germplasm of Brassica and allied crops (tribe Brassiceae, Brassicaceae). 3rd ed. Part III - Interspecific and Intergeneric Hybridization in the Tribe Brassiceae (Cruciferae).

435: NCBI --> there is no reference/URL given about this non spelled acronym

436-37: "The Wild Germplasm of Brassica [37]" does not seem to correspond to reference 37: Warwick

et al. 2009. You say this was "accessed on November 2022". What is the URL of this web site?
437: Brassibase [9] (accessed on November 2022)--> the URL is missing.
438: Plant DNA C-values database [93] (accessed on November 2022) --> the URL is missing
438-39: plant CCDB database [95] (accessed on November 2022) --> the URL is missing and in case you mean http://ccdb.tau.ac.il/, please note that this site is not working, which is rather frustrating. Hopefully you can point at a functional web site.
455: "USDA GRIN Global [94]" --> GRIN Global is a database platform, i.e. a software that can be adopted by genebanks around the world. It is not a searchable database per se. You are possibly confusing it with the USDA GRIN Taxonomy database. To eliminate ambiguities, you need to indicate the URL and the correct title of what you have browsed.
456: "inventory of the Global Diversity Trust" --> Surely this inventory has a more specific name. Possibly you are referring to the "Harlan and De Wet CWR inventory", which is however no longer maintained. You should use the proper name and indicate the URL of the web site that you accessed to avoid ambiguity.
458: "TRY database" --> It would be useful to include the URL and date of access.
462 and 466: Every time that you indicate that you accessed a web site, you should include the URL, that is for World Online and BGCI.
722: Reference 94: the URL of the web site should be added.
779 and following: The cross between Brassica oleracea and Brassica cretica has been successful, as you say in line 332. Therefore, you should indicate (Y) after B. cretica in this cross.
Figure 3: These maps are very interesting. However, you have built them based on the starting list of 1,242 species. It is understandable, since many of these still need a lot of investigation and offer potential. However, after your study, your main result is a new list of CWR with a high potential to hybridize with crops. This becomes a priority list from a utilitarian point of view. Thus, it would be very interesting to also see maps dedicated to the new priority list. This would be a great help for prioritization of action.
Table S1 legenda: it is not clear from where you got the data of table S1. When you say "Global Crop Trust Diversity", you probably intend 'Global Crop Diversity Trust' instead, but this is not a well identified web site, it is just the name of an organization. And by 'USDA Global-GRIN', you probably mean 'USDA GRIN-Global', which is not an identified web site.

Methods

Are the methods appropriate to the aims of the study, are they well described, and are necessary controls included? Choose an item.

Conclusions

Are the conclusions adequately supported by the data shown? Choose an item.

Reporting Standards

Does the manuscript adhere to the journal's guidelines on [minimum standards of reporting?] Choose an item.

Choose an item.

Statistics

Are you able to assess all statistics in the manuscript, including the appropriateness of statistical tests used? Choose an item.

Quality of Written English

Please indicate the quality of language in the manuscript: Choose an item.

Declaration of Competing Interests

Please complete a declaration of competing interests, considering the following questions:

- Have you in the past five years received reimbursements, fees, funding, or salary from an organisation that may in any way gain or lose financially from the publication of this manuscript, either now or in the future?
- Do you hold any stocks or shares in an organisation that may in any way gain or lose financially from the publication of this manuscript, either now or in the future?
- Do you hold or are you currently applying for any patents relating to the content of the manuscript?
- Have you received reimbursements, fees, funding, or salary from an organization that holds or has applied for patents relating to the content of the manuscript?
- Do you have any other financial competing interests?
- Do you have any non-financial competing interests in relation to this paper?

If you can answer no to all of the above, write 'I declare that I have no competing interests' below. If your reply is yes to any, please give details below.

I declare that I have no competing interests

I agree to the open peer review policy of the journal. I understand that my name will be included on my report to the authors and, if the manuscript is accepted for publication, my named report including any attachments I upload will be posted on the website along with the authors' responses. I agree for my report to be made available under an Open Access Creative Commons CC-BY license (http://creativecommons.org/licenses/by/4.0/). I understand that any comments which I do not wish to be included in my named report can be included as confidential comments to the editors, which will not be published.

Choose an item.

To further support our reviewers, we have joined with Publons, where you can gain additional credit to further highlight your hard work (see: https://publons.com/journal/530/gigascience). On publication of this paper, your review will be automatically added to Publons, you can then choose whether or not to claim your Publons credit. I understand this statement.

Yes Choose an item.
